# Synthetic Studies on Amphidinolide F: Exploration of Macrocycle Construction by Intramolecular Stille Coupling

**DOI:** 10.1021/acs.orglett.2c03045

**Published:** 2022-10-12

**Authors:** Ludovic Decultot, J. Stephen Clark

**Affiliations:** School of Chemistry, University of Glasgow, Joseph Black Building, University Avenue, Glasgow G12 8QQ, U.K.

## Abstract

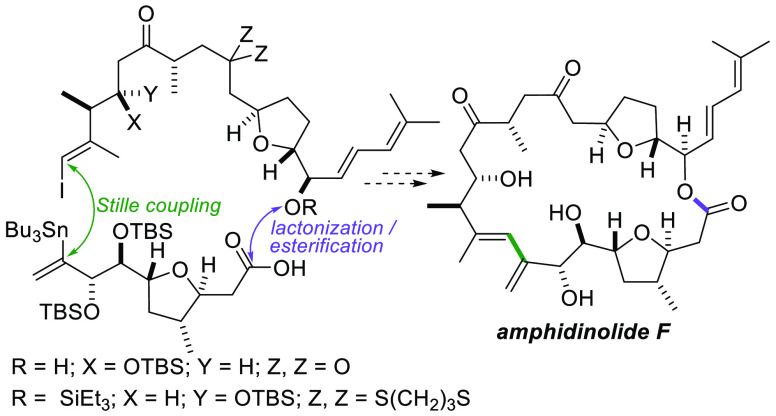

Exploration of an ambitious new strategy for the total synthesis of the cytotoxic marine natural product amphidinolide F is described, which features fabrication of the core structure from four readily accessible fragments and macrocycle construction through C9–C10 bond formation by intramolecular Stille coupling between an alkenyl iodide and alkenyl stannane. Efficient stereoselective synthesis of each of the four building-blocks and subsequent coupling of them to produce the requisite cyclization precursor has been accomplished, but suitable conditions for high-yielding palladium-mediated closure of the macrocycle to produce the fully protected amphidinolide F ring system have yet to be identified.

Amphidinolide F is a structurally complex cytotoxic marine natural product produced by a dinoflagellate of the genus *Amphidinium* (Figure 1). The isolation of amphidinolide F from cultures of the dinoflagellate and its subsequent characterization were reported by the group of Kobayashi in 1991.^1^ The complete structure of amphidinolide F and both the relative and absolute configurations of the 11 stereogenic centers embedded in it were assigned by comparison of NMR data with the data for amphidinolide C,^2^ a closely related natural product that had been isolated and characterized by Kobayashi and co-workers prior to the isolation of amphidinolide F. This work established that the macrolactone cores of amphidinolides F and C are identical; the structure of the latter was determined by comparison of NMR data with those of key subunits prepared by de novo synthesis.^3^

**Figure 1:**
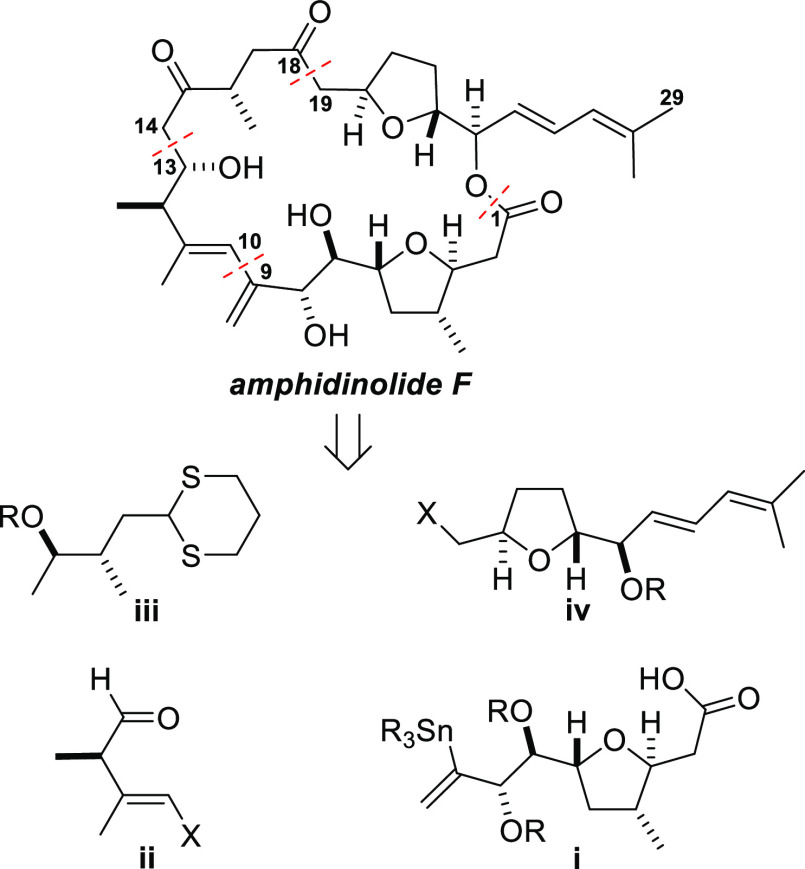
Amphidinolide F and its disconnection.

Amphidinolide F and related amphidinolides are alluring synthetic targets because of their structural complexity and reported biological activities. Myriad synthetic strategies for the stereoselective construction of key fragments of amphidinolide F have been explored in recent years, and many of them are also directly applicable to the synthesis of members of the amphidinolide C series because of the structural similarity of the compounds.^4−14^ This work has resulted in the total syntheses of amphidinolide F by the groups of Fürstner,^15^ Carter,^16^ and Ferrié;^17^ syntheses of amphidinolides C and C2 have also been completed by these research groups.

We have already reported the synthesis of the C1–C17 and the C18–C29/C18–C34 fragments of amphidinolides F, C, C2, and C3.^18^ More recently, we have constructed the entire C1–C29 framework of amphidinolide F by a convergent route in which fragments corresponding to C1–C9, C10–C17, and C18–C29 were coupled.^19^ Although the latter approach delivered the required linear C1–C29 precursor required for formation of the lactone by direct cyclization, problems were encountered when the C1–C17 segment was coupled to the C18–C29 fragment at a late stage in the synthesis, and so the alternative synthetic strategy described herein was explored.

The new strategy evolved from a retrosynthetic analysis of amphidinolide F in which the core structure is disconnected to produce four fragments (**i**–**iv**) of variable size and complexity (Figure 1). The two most complex fragments (**i** and **iv**) each contain a single tetrahydrofuran and are similar in structure to intermediates used in our recently published study. The C19–C29 fragment, which corresponds to fragment **iv** in the retrosynthetic analysis, was prepared as shown in Scheme 1. The route commenced with the known 2,5-disubstituted tetrahydrofuran **1**, which was prepared directly from an open chain γ-hydroxyalkene by use of a modified version of Mukaiyama’s cobalt-catalyzed oxidative cyclization reaction, in the manner described by Pagenkopf and co-workers.^20,21^ The alcohol **1** was subjected to oxidation, and the resulting aldehyde was reacted with a Grignard reagent generated from trimethylsilylacetylene. Removal of the trimethylsilyl group then delivered the alcohol **2** as a mixture of diastereomers at the propargylic stereogenic center. A palladium-mediated Sonogashira coupling reaction between the alkyne **2** and 1-bromo-2-methyl-1-propene afforded the enyne **3**, and subsequent alkyne reduction with Red-Al produced the corresponding diene with excellent Z-selectivity.^22,23^ Dess–Martin oxidation of the diastereomeric mixture of allylic alcohols (**5a** and **5b**) afforded the ketone **4**, and diastereoselective reduction of the carbonyl group under Luche conditions yielded the alcohol **5a** (8:1, **5a**:**5b**). Stereochemical assignment at the hydroxy-bearing stereogenic center (C24) was made based on literature precedent and the outcome of Luche reduction reactions of closely related ketones in our own previous work,^24,18b^ and the subsequent use of the reaction for the reduction of analogous substrates during the synthesis of amphidinolide F by Ferrié and co-workers.^17^ Protection of the free secondary hydroxyl group as a *tert*-butyldimethylsilyl (TBS) ether and deprotection of the primary hydroxyl group produced the alcohol **6**, which corresponds to fragment **iv** in the retrosynthetic analysis (Figure 1).

**Scheme 1:**
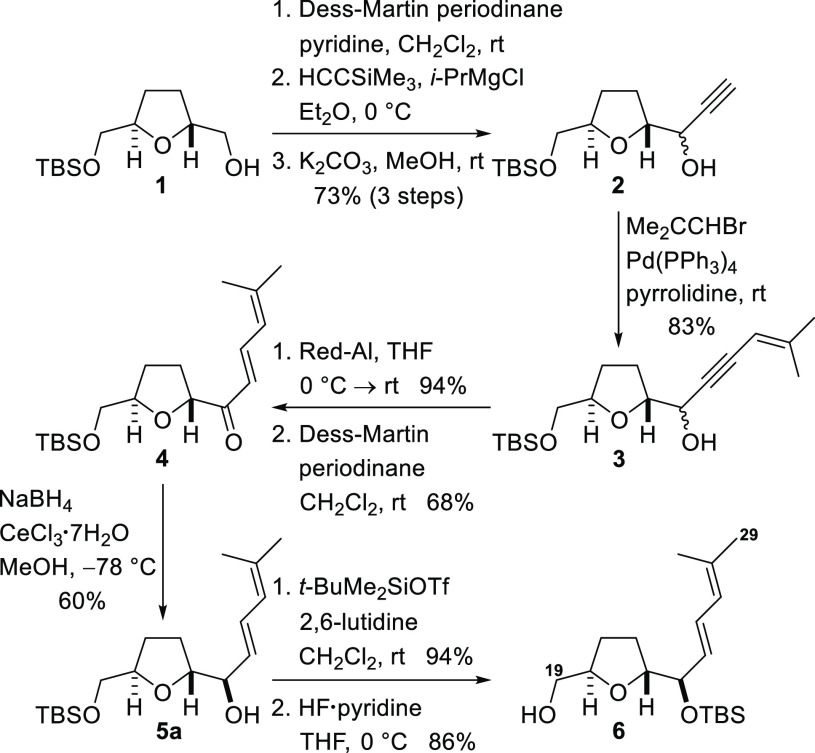
Preparation of the C19–C29 Fragment

Synthesis of the C14–C18 fragment that corresponds to fragment **iii** in the retrosynthetic analysis (Figure 1) commenced with the known β-hydroxy ester **7**, which was prepared by Fráter–Seebach alkylation of commercially available methyl (*R*)-3-hydroxybutyrate (Scheme 2).^25^ The hydroxyl group of the β-hydroxy ester **7** was first protected as the 1-ethoxyethyl ether and the ester group was reduced with lithium aluminum hydride to provide the primary alcohol **8**. The alcohol was converted into the corresponding iodide, and subsequent nucleophilic displacement with lithiated 1,3-dithiane afforded the C14–C18 fragment **9** suitable for attachment to the C19–C29 fragment.

**Scheme 2:**
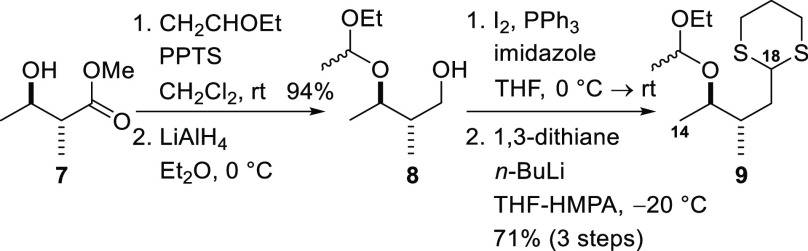
Preparation of the C14–C18 Fragment

The starting compound for synthesis of the C10–C13 fragment was the known alkyne **10**, which was prepared from commercially available methyl (*S*)-3-hydroxy-2-methyl-butyrate by a five-step sequence, analogous to that described by Lee and co-workers (Scheme 3).^26^ The alkyne **10** was converted into the alkenyl iodide **11** by zirconium-mediated carboalumination followed by quenching with iodine according to Negishi’s protocol,^27^ as performed by Maier and co-workers on an analogous alkyne.^28^ Subsequent cleavage of the silyl ether delivered the alcohol **12**. Treatment with Dess–Martin periodinane produced the aldehyde **13**, which corresponds to fragment **ii** in the retrosynthetic analysis (Figure 1).

**Scheme 3:**
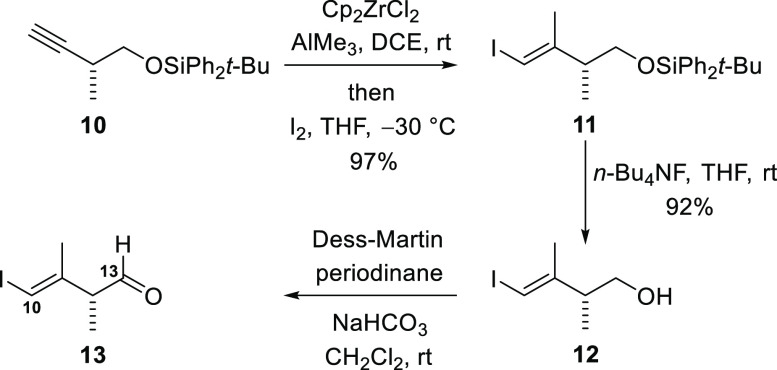
Preparation of the C10–C13 Fragment

The final fragment—C1–C9—required for the synthesis was obtained by functionalization of the ester **14**, a compound we had used in our previously published work on the synthesis of amphidinolide F (Scheme 4).^19^ Thus, reductive cleavage of the pivaloyl group from the ester **14** afforded the alcohol **15**. Dess–Martin oxidation of the alcohol **15** to give the aldehyde **16** and subsequent Pinnick oxidation delivered the carboxylic acid **17** (fragment **i** in Figure 1).^15,17^

**Scheme 4:**
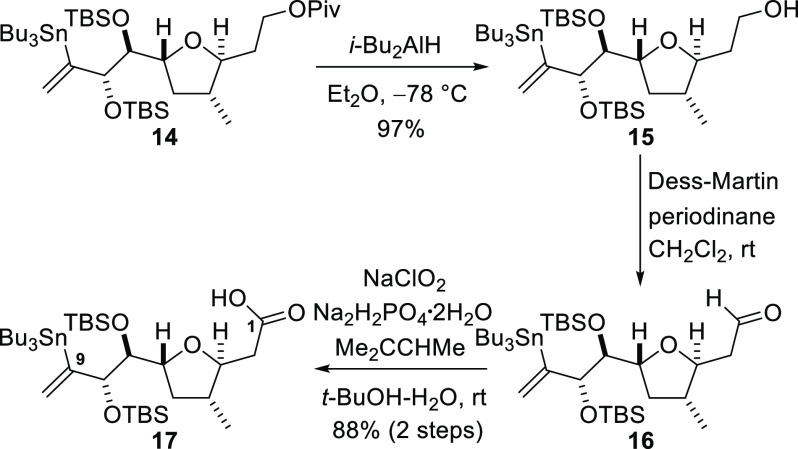
Functionalization of the C1–C9 Fragment

Completion of the syntheses of the C1–C9, C10–C13, C14–C18, and C19–C29 fragments allowed construction of the complete framework of amphidinolide F to be explored. Coupling commenced with attachment of the C14–C18 fragment to the C19–C29 fragment (Scheme 5). The alcohol **6** was first converted into the corresponding iodide by treatment with iodine and triphenylphosphine. Subsequent fragment coupling was accomplished by nucleophilic attack of the iodide with the anion generated by deprotonation of the dithiane **9** with *tert*-butyllithium. Removal of the ethoxyethyl protecting group from the coupled product **18** under acidic conditions delivered the alcohol **19** in 44% yield over three steps. Parikh–Doering oxidation of the alcohol produced the ketone **20** and subsequent removal of the TBS protecting group revealed the alcohol **21**, which was immediately reprotected as the more labile triethylsilyl (TES) ether to give the ketone **22**.

**Scheme 5:**
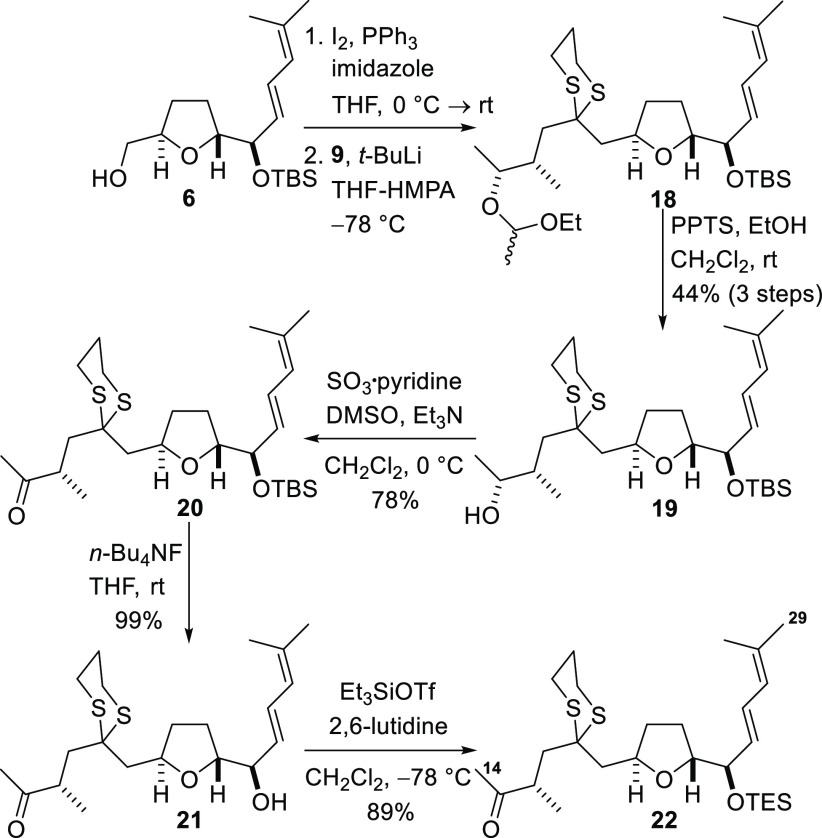
Fragment Coupling to Produce the C14–C29 Segment

Ketone **22** corresponds to the C14–C29 segment of the natural product and possesses the requisite functionality for attachment of the C10–C13 fragment by an aldol condensation reaction (Scheme 6). Generation of a boron enolate by treatment of the methyl ketone **22** with dicyclohexylboron chloride and triethylamine followed by addition of the aldehyde **13** at −78 °C afforded the diastereomeric alcohols **23a** and **23b** (2.2:1). The configuration at the newly created hydroxyl-bearing stereogenic center was made by conversion of the alcohol **23a** into diastereomeric Mosher esters and subsequent ^1^H NMR analysis according to the protocol of Hoye and co-workers (see the Supporting Information).^29^ Chromatographic separation of the alcohols was challenging, but samples of each diastereomer were isolated and then protected as TBS ethers to give the ketones **24a** and **24b**.

**Scheme 6:**
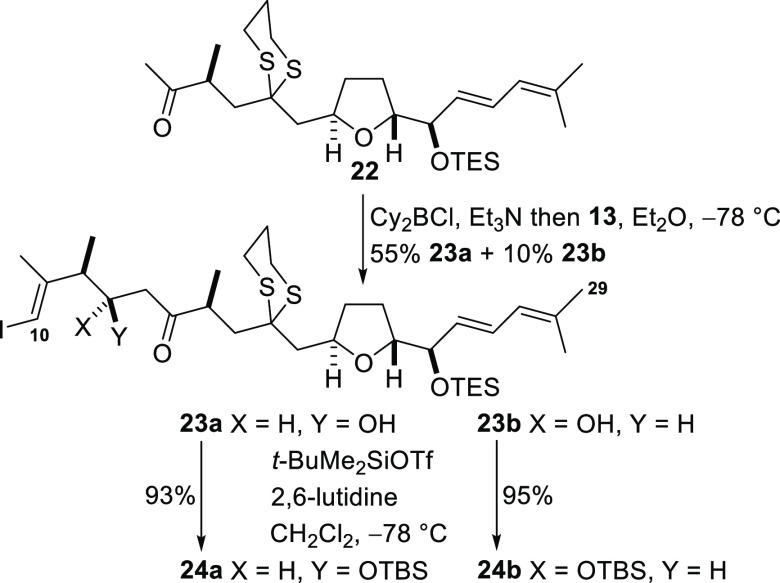
Construction of the C10–C29 Segment

Construction of the C10–C29 segment meant that coupling to the C1–C9 fragment to produce the entire C1–C29 framework of amphidinolide F could be explored. The first approach that was investigated involved direct intermolecular Stille coupling of the vinylic stannanes **15** and **17**, corresponding to the C1–C9 fragment, to the C10–C29 iodide **24b** (Scheme 7). In recent studies performed by us, Stille coupling had been used to attach the vinylic stannane **14** (Scheme 4) to a truncated C10–C17 fragment.^19^ This reaction had proceeded in high yield, and so the proposed coupling reaction was not expected to be problematic. However, when the reagents and conditions used previously were employed perform Stille coupling between the alkenyl iodide **24b** and either vinylic stannane **15** or **17**, neither of the expected coupled products **25** or **26** was obtained. The failure of the coupling reaction was both unexpected given that Ferrié and co-workers were able to couple the vinylic stannane **17** to a very closely related analogue of the C10–C29 segment **24b** under similar reaction conditions during their recent synthesis of amphidinolide F.^17^ Alternative Stille reaction conditions are clearly required to accommodate the bulky alkenyl iodide **24b** and/or the acidic coupling partners **15** and **17**.

**Scheme 7:**
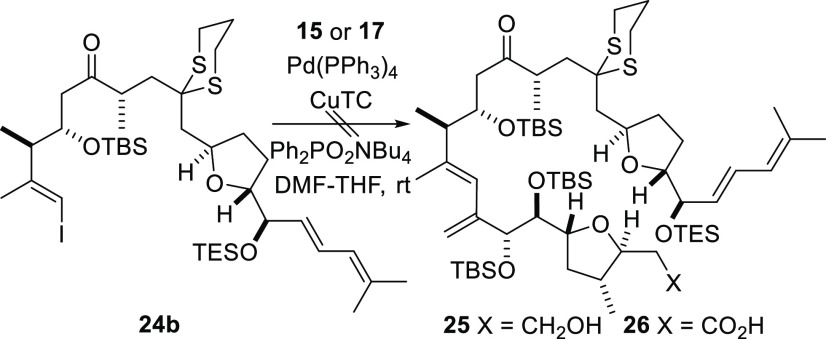
Attempted Intermolecular Stille Coupling of the C1–C9 Fragment to the C10–C29 Segment

The failure of the direct intermolecular Stille coupling reaction to deliver either of the expected coupled products (**25** or **26**) corresponding to the C1–C29 framework of amphidinolide F meant that a new endgame strategy was required. The decision was made to investigate an alternative route in which the reactions used to assemble the complete carbon framework and construct the macrocycle were reordered. We opted for an approach in which an ambitious intramolecular Stille coupling reaction would be employed to accomplish simultaneous formation of the complete carbon framework and the macrolactone in a single operation (Scheme 8).^30^ To investigate this approach, the C18 carbonyl group and the C24 hydroxyl group in the C10–C29 segment **24a** (Scheme 6) were unmasked by hydrolysis of the dithiane group under standard conditions with concomitant cleavage of the TES ether. The resulting alcohol **27** was then subjected to esterification with the carboxylic acid **17** under standard Yamaguchi conditions^31^ to produce the ester **28** in good yield. Intramolecular Stille coupling to produce the macrolactone **29** was then explored. Global deprotection of the lactone **29** would deliver 13-*epi*-amphidinolide F, and it was anticipated that the diastereomeric compound **24b** would be subjected to a parallel sequence of reactions to give amphidinolide F. Attempted intramolecular Stille coupling reaction of the ester **28** to give the lactone **29** produced a complex mixture of products, and so we attempted to isolate 13-*epi*-amphidinolide F by immediate deprotection of the crude material. However, the required product was not isolated after complete silyl ether cleavage to reveal the free hydroxyl groups at C7, C8, and C13.

**Scheme 8:**
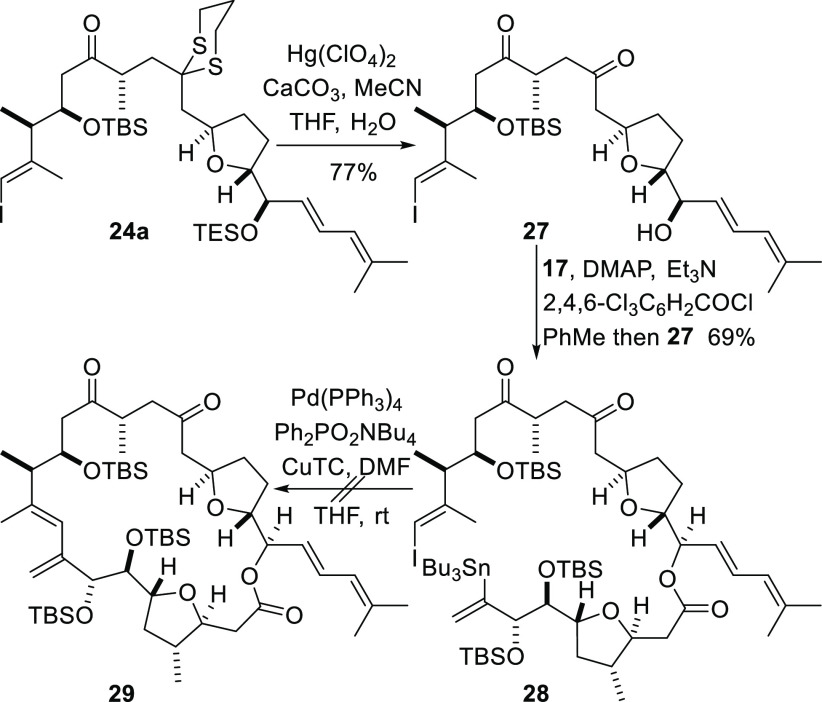
Attempted Simultaneous Construction of the C1–C29 Framework and the Macrolactone

In summary, an innovative new strategy for the total synthesis of the amphidinolide F has been investigated in which macrocycle formation was to be accomplished by an intramolecular Stille coupling reaction. Fragments that correspond to C1–C9, C10–C13, C14–C18, and C19–C29 units were prepared from readily available starting materials in an efficient and stereoselective manner, and then coupled to provide the substrate required for the proposed macrocyclization reaction. A limited number of reaction conditions have been explored for the intramolecular Stille coupling reaction to give fully protected amphidinolide F. However, further studies are required to identify the appropriate palladium catalyst and reaction conditions necessary to effect high-yielding macrocyclization.

## Supporting Information Available

Experimental procedures for preparation of all new compounds along with characterization data and copies of ^1^H and ^13^C NMR spectra (DOCX)
