# Supplementary material for: Synthetic Studies on Amphidinolide F: Exploration of Macrocycle Construction by Intramolecular Stille Coupling
Source: Org Lett. 2022 Oct 12;24(41):7600–4. doi: 10.1021/acs.orglett.2c03045 (PMC9594353; doi:10.1021/acs.orglett.2c03045)

Synthetic Studies on Amphidinolide F: Exploration of Macrocycle Construction by Intramolecular Stille Coupling

Ludovic Decultot and J. Stephen Clark*

School of Chemistry, University of Glasgow, Joseph Black Building,

University Avenue, Glasgow G12 8QQ, United Kingdom

stephen.clark@glasgow.ac.uk

# *Supporting Information*

**Table of Contents**

General Experimental SI-2

Experimental Procedures and Characterization SI-3

References SI-24

^1^H and ^13^C NMR Spectra for New Compounds SI-25

**General Experimental**

Reagents were purchased from commercial suppliers and were used without purification, unless otherwise stated. Air and moisture sensitive reactions were performed under an atmosphere of argon in flame dried apparatus. Tetrahydrofuran, toluene, acetonitrile, dichloromethane and diethyl ether were purified using a Pure-SolvTM 500 Solvent Purification System. Petroleum ether used for chromatography was the 40–60 °C fraction. All reactions were monitored by thin layer chromatography (TLC) using Merck silica gel 60 coated aluminium backed plates F254. TLC plates were visualized under UV light and stained using potassium permanganate solution, acidic ethanolic anisaldehyde solution or phosphomolybdic acid solution. Flash column chromatography was performed with silica gel (Fluorochem LC60A 35−70 µm, or Geduran Si 60 35−70 µm) as solid support. IR spectra were recorded using a Shimadzu FT IR-8400S ATR instrument. The IR spectrum of each compound (solid or liquid) was acquired directly on a thin layer of the compound at ambient temperature. ^1^H NMR spectra were recorded at ambient temperature on Bruker Avance III 400 MHz and 500 MHz spectrometers. ^13^C NMR spectra were recorded at ambient temperature on Bruker Avance III 400 MHz and 500 MHz spectrometers at 101 MHz and 126 MHz respectively. Optical rotation data were recorded using an Autopol V polarimeter. High- and low-resolution mass spectra (HRMS) were performed by use of positive or negative ion electrospray techniques on a Bruker micrOTOF-Q instrument.

**Experimental Procedures and Characterisation**

**(*R*)-1-{(2*R*,5*R*)-5-[(*tert*-Butyldimethylsilyloxy)methyl]tetrahydrofuran-2-yl}prop-2-yn-1-ol and (*S*)-1-{(2*R*,5*R*)-5-[(*tert*-butyldimethylsilyloxy)methyl]tetrahydrofuran-2-yl}prop-2-yn-1-ol (2).**

To a solution of alcohol **1**^1^ (3.20 g, 13.0 mmol) in dichloromethane (87 mL) at rt, was added pyridine (4.1 mL, 51 mmol), followed by a portion-wise addition of Dess-Martin periodinane (7.16 g, 16.9 mmol). The resulting mixture was stirred at rt for 2.5 h, before the addition of a mixture (1:1) of saturated aqueous sodium sulfite solution and saturated aqueous sodium bicarbonate solution (180 mL). The biphasic mixture was stirred vigorously for 10 min and the phases were separated. The aqueous phase was extracted with ether (3 × 150 mL) and the combined organic extracts were washed with brine (400 mL), dried over magnesium sulfate, filtered and concentrated. Residual material was used directly in the subsequent reaction without purification.

To a solution of trimethylsilylacetylene (8.6 mL, 60 mmol) in ether (62 mL) at 0 °C, was added isopropylmagnesium chloride (33 mL of a 1.6 M solution in THF, 53 mmol) dropwise from an addition funnel. The brown suspension was stirred at 0 °C for 1 h and transferred by cannula to a solution of aldehyde ether (130 mL) at 0 °C. The resulting yellow suspension was stirred at rt for 2.5 h and cooled to 0 °C. The reaction was quenched by the slow addition of a mixture (3:1) of saturated aqueous ammonium chloride solution and water (400 mL). The phases were separated, and the aqueous phase was extracted with ether (3 × 250 mL). The combined organic extracts were washed with brine (700 mL), dried over magnesium sulfate, filtered and concentrated. The unpurified product was used directly in the subsequent reaction.

To a solution of the propargylic alcohol in wet methanol (87 mL) at rt, was added potassium carbonate (3.6 g, 26 mmol) in one portion. The resulting suspension was stirred at rt for 2 h, after which volatiles were removed *in vacuo*, and the residue partitioned between dichloromethane (80 mL) and saturated aqueous ammonium chloride solution (80 mL). The phases were separated, and the aqueous phase was extracted with ether (3 × 80 mL). The combined organic extracts were washed with brine (240 mL), dried over magnesium sulfate, filtered and concentrated. Residual material was purified by silica gel chromatography (pet. ether / ethyl acetate, 85:15) to give a diastereomeric mixture (1.3:1) of the propargylic alcohol **2** (2.58 g, 73% over three steps) as a yellow oil. R*_f_* = 0.28 (pet. ether / ethyl acetate, 85:15); ν_max._ 3414, 3312, 2955, 2928, 2897, 2885, 2857, 629 cm^−1^; ^1^H NMR (400 MHz, CDCl_3_) δ 4.43 (1H minor, ddd, *J* = 5.9, 3.6, 2.3 Hz), 4.20 (1H major, ddd, *J* = 6.8, 4.7, 2.2 Hz), 4.21−4.07 (2H major + 2H minor, m), 3.66−3.59 (2H major + 2H minor, m), 2.50 (1H major, d, *J* = 4.7 Hz), 2.44 (1H major, d, *J* = 2.2 Hz), 2.43 (1H minor, d, *J* = 2.3 Hz), 2.39 (1H minor, d, *J* = 5.9 Hz), 2.16−1.95 (6H, m), 1.88−1.75 (4H, m), 0.89 (9H minor, s), 0.89 (9H major, s), 0.06 (6H major + 6H minor, s); ^13^C NMR (101 MHz, CDCl_3_) δ 82.4, 82.2, 82.0, 81.7, 81.3, 80.5, 74.1, 73.8, 65.9, 65.7, 65.3, 64.6, 28.1, 28.04, 28.03, 26.6, 26.08, 26.07, 18.5, 18.5, −5.1, −5.1, −5.2, −5.2; HRMS (ESI+) *m*/*z*: [M+Na]^+^ calcd for C_14_H_26_O_3_SiNa 293.1543, found 293.1532.

**(*R*)-1-{(2*R*,5*R*)-5-[(*tert*-Butyldimethylsilyloxy)methyl]tetrahydrofuran-2-yl}-5-methylhex-4-en-2-yn-1-ol and (*S*)-1-{(2*R*,5*R*)-5-[(*tert*-butyldimethylsilyloxy)methyl]tetrahydrofuran-2-yl}-5-methylhex-4-en-2-yn-1-ol (3).**

To a suspension of tetrakis(triphenylphosphine)palladium(0) (220 mg, 0.190 mmol) in pyrrolidine (4.5 mL) at rt, was added 1-bromo-2-methyl-1-propene (1.54 g, 11.4 mmol). The resulting suspension was stirred at rt for 5 min, before the addition of a solution of alkyne **2** (1.03 g, 3.81 mmol) in pyrrolidine (4.5 mL). The reaction mixture was heated to 50 °C (oil bath) stirred for 15 h and allowed to cool to rt. The solution was diluted with ether (40 mL) and saturated aqueous ammonium chloride solution (50 mL) was slowly added. The layers were separated, and the aqueous phase was extracted with ether (3 × 40 mL). The combined organic extracts were washed with brine (120 mL), dried over magnesium sulfate, filtered and concentrated. Residual material was purified by silica gel chromatography (pet. ether / ethyl acetate, 90:10) to deliver a diastereomeric mixture (1.3:1) of enyne **3** (1.03 g, 83%) as a yellow oil. R*_f_* = 0.26 (pet. ether / ethyl acetate, 90:10); ν_max._ 3418, 2953, 2928, 2857, 837, 777 cm^−1^; ^1^H NMR (400 MHz, CDCl_3_) δ 5.29−5.25 (1H major + 1H minor, m), 4.60 (1H minor, ddd, *J* = 5.6, 3.3, 1.9 Hz), 4.34 (1H major, ddd, *J* = 7.0, 4.1, 1.7 Hz), 4.22−4.15 (2H minor, m), 4.13−4.06 (2H major, m), 3.66−3.58 (2H major + 2H minor, m), 2.48 (1H major, d, *J* = 4.1 Hz), 2.33 (1H minor, d, *J* = 5.6 Hz), 2.16−1.96 (4H major, m), 1.88 (3H major + 3H minor, br s), 1.86−1.75 (4H minor, m), 1.80 (3H major + 3H minor, br s), 0.89 (9H minor, s), 0.89 (9H major, s), 0.06 (6H major + 6H minor, s); ^13^C NMR (101 MHz, CDCl_3_) δ 149.59, 149.58, 104.8, 104.7, 89.1, 89.0, 84.0, 83.8, 82.8, 82.1, 81.2, 80.4, 66.2, 66.0, 65.8, 65.3, 28.3, 28.2, 28.1, 26.6, 26.10, 26.08, 24.9, 21.20, 21.18, 18.53, 18.51, −5.1, −5.1, −5.2, −5.2; HRMS (ESI+) *m*/*z*: [M+Na]^+^ calcd for C_18_H_32_O_3_SiNa 347.2013, found 347.2005.

**(*R*,*E*)-1-{(2*R*,5*R*)-5-[(*tert*-Butyldimethylsilyloxy)methyl]tetrahydrofuran-2-yl)-5-methylhexa-2,4-dien-1-ol (5a) and (*S*,*E*)-1-{(2*R*,5*R*)-5-[(*tert*-Butyldimethylsilyloxy)methyl]tetrahydrofuran-2-yl)-5-methylhexa-2,4-dien-1-ol (5b).**

To a solution of enyne **3** (2.15 g, 6.62 mmol) in THF (66 mL) at 0 °C, was added sodium bis(2-methoxyethoxy)aluminium hydride (8.6 mL of ≥ 60 wt % in toluene, 27 mmol) dropwise. The resulting cloudy mixture was stirred at rt for 2 h and cooled to 0 °C, before the dropwise addition of saturated aqueous potassium sodium tartrate solution (100 mL). The phases were separated, and the aqueous phase was extracted with ether (3 × 90 mL). The combined organic extracts were washed with brine (250 mL), dried over magnesium sulfate, filtered and concentrated. Residual material was purified by silica gel chromatography (pet. ether / ethyl acetate, 90:10) to deliver a diastereomeric mixture (1.25:1) of the dienes **5a**,**b** (2.04 g, 94%) as a colorless oil. ν_max._ 3435, 2955, 2928, 2907, 2897, 2886, 2857, 1661, 988, 961, 939, 814, 777 cm^−1^; ^1^H NMR (500 MHz, CDCl_3_) δ 6.52 (1H major, ddd, *J* = 15.2, 11.0, 1.1 Hz), 6.50 (1H minor, ddd, *J* = 15.3, 11.0, 1.1 Hz), 5.82 (1H major + 1H minor, br d, *J* = 11.0 Hz), 5.48 (1H minor, dd, *J* = 15.3, 6.6 Hz), 5.47 (1H major, dd, *J* = 15.2, 6.9 Hz), 4.34 (1H minor, dddd, *J* = 6.6, 3.5, 3.1, 1.1 Hz), 4.10 (1H major, app tt, *J* = 6.8, 4.9 Hz), 4.07 (1H major, app tdd, *J* = 6.9, 5.1, 4.7 Hz), 4.01 (1H minor, ddd, *J* = 7.8, 7.1, 3.5 Hz), 3.94 (1H major, app tdd, *J* = 6.9, 2.9, 1.1 Hz), 3.87 (1H major, app q, *J* = 6.9 Hz), 3.64 (1H major, dd, *J* = 10.7, 4.7 Hz), 3.62 (1H minor, dd, *J* = 10.7, 4.9 Hz), 3.59 (1H major, dd, *J* = 10.7, 5.0 Hz), 3.59 (1H minor, dd, *J* = 10.7, 4.9 Hz), 2.55 (1H major, d, *J* = 2.9 Hz), 2.15 (1H minor, d, *J* = 3.1 Hz), 2.02−1.91 (2H major, m), 1.88−1.82 (2H minor, m), 1.77 (3H major + 3H minor, br s), 1.76 (3H major + 3H minor, br s), 1.78−1.62 (2H major + 2H minor, m), 0.89 (9H minor, s), 0.89 (9H major, s), 0.06 (6H major, s), 0.06 (6H minor, s); ^13^C NMR (126 MHz, CDCl_3_) δ 136.5, 136.3, 129.3, 128.8, 128.6, 128.2, 124.61, 124.58, 82.9, 82.5, 80.7, 79.9, 75.6, 73.5, 66.1, 65.9, 28.4, 28.3, 28.0, 26.2, 26.11, 26.08, 25.6, 18.6, 18.55, 18.52, 18.51, −5.11, −5.12, −5.13; HRMS (ESI+) *m*/*z*: [M+Na]^+^ calcd for C_18_H_34_O_3_SiNa 349.2169, found 349.2153.

**(*E*)-1-{(2*R*,5*R*)-5-[(*tert*-Butyldimethylsilyloxy)methyl]tetrahydrofuran-2-yl}-5-methylhexa-2,4-dien-1-one (4).**

To a solution of allylic alcohols **5a**,**b** (494 mg, 1.51 mmol) in dichloromethane (10 mL) at rt, was added Dess-Martin periodinane (834 mg, 1.97 mmol) in one portion. The resulting mixture was stirred at rt for 1 h, followed by the addition of a mixture (1:1) of saturated aqueous sodium sulfite solution and saturated aqueous sodium bicarbonate solution (20 mL). The phases were separated and the aqueous phase was extracted with ether (3 × 20 mL). The combined organic extracts were washed with brine (60 mL), dried over magnesium sulfate, filtered and concentrated. Residual material was purified by silica gel chromatography (pet. ether / ethyl acetate, 95:5) to deliver the enone **4** (333 mg, 68%) as a colorless oil. R*_f_* = 0.34 (pet. ether / ethyl acetate, 90:10); ^1^H NMR (500 MHz, CDCl_3_) δ 7.62 (1H, dd, *J* = 15.2, 11.7 Hz), 6.42 (1H, d, *J* = 15.2 Hz), 6.03 (1H, br d, *J* = 11.7 Hz), 4.56 (1H, dd, *J* = 7.9, 6.2 Hz), 4.22 (1H, app tt, *J* = 6.7, 4.5 Hz), 3.67 (2H, d, *J* = 4.5 Hz), 2.29−2.22 (1H, m), 1.99−1.92 (2H, m), 1.92 (3H, br s), 1.89 (3H, br s), 1.86−1.79 (1H, m), 0.90 (9H, s), 0.07 (3H, s), 0.07 (3H, s).

**(*R*,*E*)-1-{(2*R*,5*R*)-5-[(*tert*-Butyldimethylsilyloxy)methyl]tetrahydrofuran-2-yl}-5-methylhexa-2,4-dien-1-ol (5a).**

To a solution of enone **4** (333 mg, 1.03 mmol) in methanol (10 mL) at −78 °C, were added cerium(III) chloride heptahydrate (535 mg, 1.44 mmol) and sodium borohydride (54 mg, 1.4 mmol) sequentially. The resulting mixture was stirred at −78 °C for 45 min and concentrated. The residue was partitioned between ether (30 mL) and saturated aqueous ammonium chloride solution (30 mL). The phases were separated, and the aqueous phase was extracted with ether (3 × 30 mL). The combined organic extracts were washed with brine (100 mL), dried over magnesium sulfate, filtered and concentrated. Residual material (~8:1 mixture of diastereomers) was purified by silica gel chromatography (pet. ether / ethyl acetate, 95:5) to deliver allylic alcohol **5a** (200 mg, 0.61 mmol, 60%) as a colorless oil. R*_f_* = 0.33 (pet. ether / ethyl acetate, 90:10); ${[\alpha]}_{D}^{21}$ +13.6 (*c* = 1.29, CHCl_3_); ν_max._ 3457, 2955, 2928, 2901, 2883, 2857, 1661, 988, 961, 835, 814, 777 cm^−1^; ^1^H NMR (500 MHz, CDCl_3_) δ 6.52 (1H, ddd, *J* = 15.1, 11.0, 1.1 Hz), 5.82 (1H, br d, *J* = 11.0 Hz), 5.47 (1H, dd, *J* = 15.1, 6.9 Hz), 4.07 (1H, app tdd, *J* = 6.9, 5.0, 4.7 Hz), 3.94 (1H, app tdd, *J* = 6.9, 2.9, 1.1 Hz), 3.87 (1H, app q, *J* = 6.9 Hz), 3.64 (1H, dd, *J* = 10.6, 4.7 Hz), 3.59 (1H, dd, *J* = 10.6, 5.0 Hz), 2.54 (1H, d, *J* = 2.9 Hz), 2.02−1.91 (2H, m), 1.77 (3H, br s), 1.76 (3H, br s), 1.80−1.72 (1H, m), 1.70−1.62 (1H, m), 0.89 (9H, s), 0.06 (6H, s); ^13^C NMR (126 MHz, CDCl_3_) δ 136.5, 129.3, 128.6, 124.6, 82.9, 79.9, 75.6, 65.9, 28.4, 28.0, 26.2, 26.1, 18.52, 18.51, −5.11, −5.13; HRMS (ESI+) *m*/*z*: [M+Na]^+^ calcd for C_18_H_34_O_3_SiNa 349.2169, found 349.2176.

***tert*-Butyl({(2R,5R)-5-[(*R*,*E*)-1-(*tert*-butyldimethylsilyloxy)-5-methylhexa-2,4-dien-1-yl]tetra-hydrofuran-2-yl}methoxy)dimethylsilane (S1).**

To a solution of alcohol **5a** (589 mg, 1.80 mmol) in dichloromethane (18 mL) at −78 °C, were added 2,6-lutidine (0.54 mL, 4.7 mmol) and *tert*-butyldimethylsilyl trifluoromethansulfonate (0.54 mL, 2.4 mmol) sequentially. The resulting solution was stirred at −78 °C for 1 h, before the addition of water (18 mL) and the biphasic mixture was allowed to warm to rt. The phases were separated, and the aqueous phase was extracted with ether (3 × 15 mL). The combined organic extracts were washed with brine (50 mL), dried over magnesium sulfate, filtered and concentrated. Residual material was purified by silica gel chromatography (pet. ether / ethyl acetate, 98:2) to deliver silyl ether **S1** (746 mg, 94%) as a colorless oil. R*_f_* = 0.38 (pet. ether / ethyl acetate, 98:2); ${[\alpha]}_{D}^{25}$ +30.8 (*c* = 1.19, CHCl_3_); ν_max._ 2955, 2928, 2897, 2886, 2857, 988, 961, 814, 775 cm^−1^; ^1^H NMR (500 MHz, CDCl_3_) δ 6.46 (1H, ddd, *J* = 15.2, 11.0, 1.5 Hz), 5.83 (1H, br d, *J* = 11.0 Hz), 5.56 (1H, dd, *J* = 15.2, 5.4 Hz), 4.23 (1H, app td, *J* = 5.4, 1.5 Hz), 4.02−3.95 (2H, m), 3.62 (1H, dd, *J* = 10.5, 4.7 Hz), 3.54 (1H, dd, *J* = 10.5, 5.4 Hz), 1.92−1.83 (2H, m), 1.77 (3H, br s), 1.75 (3H, br s), 1.74−1.61 (2H, m), 0.90 (9H, s), 0.89 (9H, s), 0.06 (3H, s), 0.05 (6H, s), 0.04 (3H, s); ^13^C NMR (126 MHz, CDCl_3_) δ 134.9, 130.0, 127.4, 124.9, 82.9, 80.1, 75.1, 66.1, 28.5, 26.8, 26.14, 26.10, 26.0, 18.52, 18.45, 18.4, −4.5, −4.6, −5.13, −5.14; HRMS (ESI+) *m*/*z*: [M+Na]^+^ calcd for C_24_H_48_O_3_Si_2_Na 463.3034, found 463.3019.

**{(2*R*,5*R*)-5-[(*R*,*E*)-1-(*tert*-Butyldimethylsilyloxy)-5-methylhexa-2,4-dien-1-yl]tetrahydrofuran-2-yl}methanol (6).**

A stock solution of HF-pyridine was prepared by mixing HF-pyridine (1.0 mL of a 70% solution of HF in pyridine), pyridine (2.0 mL) and THF (5.0 mL). To a solution of bis-silyl ether **S1** (746 mg, 1.69 mmol) in THF (169 mL) at 0 °C, was added the stock solution of HF-pyridine (19 mL) and the resulting mixture was stirred for 24 h. Further HF-pyridine (19 mL) was added, and the mixture stirred for another 36 h. The reaction was quenched by the slow addition of saturated aqueous sodium carbonate solution (60 mL) followed by the slow addition of saturated aqueous sodium bicarbonate solution (540 mL) until gas evolution ceased. The biphasic mixture was allowed to warm to rt and the phases were separated. The aqueous phase was extracted with ether (3 × 400 mL) and the combined organic extracts were washed with brine (800 mL), dried over magnesium sulfate, filtered and concentrated. Residual material was purified by silica gel chromatography (pet. ether / ethyl acetate, 85:15) to afford alcohol **6** (474 mg, 86%) as a colorless oil. R*_f_* = 0.23 (pet. ether / ethyl acetate, 85:15); ${[\alpha]}_{D}^{26}$ +18.6 (*c* = 0.765, CHCl_3_); ν_max._ 3449, 2955, 2928, 2884, 2857, 1659, 988, 961, 858, 816, 775 cm^−1^; ^1^H NMR (400 MHz, CDCl_3_) δ 6.47 (1H, ddd, *J* = 15.2, 11.0, 1.4 Hz), 5.83 (1H, br d, *J* = 11.0 Hz), 5.55 (1H, dd, *J* = 15.2, 5.7 Hz), 4.20 (1H, app td, *J* = 5.7, 1.4 Hz), 4.07 (1H, app dtd, *J* = 8.0, 6.0, 3.3 Hz), 4.00−3.93 (1H, m), 3.64 (1H, ddd, *J* = 11.6, 6.9, 3.3 Hz), 3.46 (1H, app dt, *J* = 11.6, 6.0 Hz), 1.94−1.85 (3H, m), 1.77 (3H, br s), 1.81−1.71 (1H, m), 1.75 (3H, br s), 1.69−1.60 (1H, m), 0.91 (9H, s), 0.07 (3H, s), 0.05 (3H, s); ^13^C NMR (126 MHz, CDCl_3_) δ 135.4, 129.8, 127.7, 124.8, 82.9, 79.8, 75.5, 65.1, 27.6, 27.5, 26.2, 26.0, 18.5, 18.4, −4.4, −4.6; HRMS (ESI+) *m*/*z*: [M+Na]^+^ calcd for C_18_H_34_O_3_SiNa 349.2169, found 349.2161.

**Methyl (2*R*,3*R*)-3-{[1(*R*/*S*)-ethoxy]ethoxy}-2-methylbutanoate (S2).**

To a solution of β-hydroxy ester **7**^2^ (2.00 g, 15.1 mmol) in dichloromethane (150 mL) at 0 °C, were added ethyl vinyl ether (4.4 mL, 44 mmol) and pyridinium *p*-toluenesulfonate (380 mg, 1.51 mmol) sequentially. The resulting mixture was stirred at 0 °C for 5 min and at rt for 1.5 h, then diluted with ether (450 mL) and washed with brine (350 mL). The phases were separated, and the organic phase was dried over magnesium sulfate, filtered and concentrated. Residual material was purified by silica gel chromatography (pet. ether / ethyl acetate, 95:5) to afford a diastereomeric mixture of acetals **S2** (2.90 g, 94%) as a colorless oil. R*_f_* = 0.38 (pet. ether / ethyl acetate, 90:10); ν_max._ 2978, 2938, 2884, 1738 cm^−1^; ^1^H NMR (500 MHz, CDCl_3_) δ 4.75 (1H minor, q, *J* = 5.4 Hz), 4.69 (1H major, q, *J* = 5.3 Hz), 3.96 (1H minor, dq, *J* = 7.3, 6.3 Hz), 3.87 (1H major, dq, *J* = 7.3, 6.3 Hz), 3.67 (3H major + 3H minor, s), 3.61 (1H minor, dq, *J* = 9.2, 7.1 Hz), 3.61 (1H major, dq, *J* = 9.2, 7.1 Hz), 3.48 (1H major, dq, *J* = 9.2, 7.1 Hz), 3.41 (1H minor, dq, *J* = 9.2, 7.1 Hz), 2.64 (1H minor, app p, *J* = 7.3 Hz), 2.60 (1H major, app p, *J* = 7.3 Hz), 1.29 (3H minor, d, *J* = 5.4 Hz), 1.24 (3H major, d, *J* = 5.3 Hz), 1.21−1.16 (6H major + 3H minor, m), 1.13 (3H minor, d, *J* = 7.3 Hz), 1.12 (3H minor, d, *J* = 6.3 Hz), 1.10 (3H major, d, *J* = 7.3 Hz); ^13^C NMR (126 MHz, CDCl_3_) δ 175.6, 175.5, 100.5, 98.3, 75.2, 72.5, 60.3, 60.1, 51.7, 46.02, 46.01, 20.8, 20.6, 18.2, 17.1, 15.5, 15.4, 12.59, 12.55; HRMS (ESI+) *m*/*z*: [M+Na]^+^ calcd for C_10_H_20_O_4_Na 227.1254, found 227.1245.

**(2*S*,3*R*)-3-{[1(*R*/*S*)-Ethoxy]ethoxy}-2-methylbutan-1-ol (8).**

To a suspension of lithium aluminium hydride (622 mg, 16.4 mmol) in ether (66 mL) at 0 °C, was slowly added a solution of methyl ester **S2** (1.34 g, 6.56 mmol) in ether (11 mL). The resulting mixture was stirred at 0 °C for 30 min and then the reaction was quenched by dropwise addition of water (0.62 mL) followed by 15% aqueous sodium hydroxide solution (0.62 mL) and more water (1.9 mL) at 0 °C. The mixture was stirred vigorously at rt for 30 min and then filtered through a cotton plug to remove the white solids. The filtrate was concentrated, and the crude alcohol **8** was used directly in the next step without purification. R*_f_* = 0.46 (pet. ether / ethyl acetate, 60:40); ν_max._ 3437, 2976, 2934, 2880 cm^−1^; ^1^H NMR (500 MHz, CDCl_3_) δ 4.71 (1H major, q, *J* = 5.3 Hz), 4.69 (1H minor, q, *J* = 5.2 Hz), 3.84 (1H minor, ddd, *J* = 11.2, 6.3, 3.3 Hz), 3.70−3.61 (2H major + 1H minor, m), 3.61−3.44 (3H major + 3H minor, m), 3.15 (1H minor, app t, *J* = 6.3 Hz), 2.57−2.54 (1H major, m), 1.73 (1H major, app hd, *J* = 6.9, 3.9 Hz), 1.69−1.59 (1H minor, m), 1.33 (3H major, d, *J* = 5.3 Hz), 1.31 (3H minor, d, *J* = 5.2 Hz), 1.24 (3H major, d, *J* = 6.2 Hz), 1.22 (3H minor, t, *J* = 7.0 Hz), 1.20 (3H major, t, *J* = 7.1 Hz), 1.17 (3H minor, d, *J* = 6.1 Hz), 0.94 (3H minor, d, *J* = 7.0 Hz), 0.93 (3H major, d, *J* = 6.9 Hz); ^13^C NMR (126 MHz, CDCl_3_) δ 100.3, 98.4, 78.1, 75.8, 66.4, 66.0, 61.3, 60.6, 41.5, 41.2, 20.8, 20.6, 19.1, 18.4, 15.42, 15.41, 14.6, 14.0; HRMS (ESI+) *m*/*z*: [M+Na]^+^ calcd for C_9_H_20_O_3_Na 199.1305, found 199.1304.

**2-((2*S*,3*R*)-3-{[1(*R*/*S*)-Ethoxy]ethoxy}-2-methylbutyl)-1,3-dithiane (9).**

*Note: The alkyl iodide intermediate is light-sensitive and all manipulations were performed under low-light conditions.*

To a solution of crude alcohol **8** in THF (42 mL) at 0 °C, were added triphenylphosphine (3.30 g, 12.6 mmol) and imidazole (1.72 g, 25.3 mmol). The resulting mixture was stirred at 0 °C for 10 min, before the portion-wise addition of iodine (3.20 g, 12.6 mmol). The brown solution was stirred at 0 °C for 10 min and at rt for 1 h. The reaction was quenched by the addition of saturated aqueous sodium sulfite solution (35 mL) and the phases were separated. The aqueous phase was extracted with ether (3 × 35 mL) and the combined organic extracts were washed with brine (100 mL), dried over magnesium sulfate, filtered and concentrated. The residue was filtered rapidly through a short pad of silica gel (pet. ether / ethyl acetate, 95:5) to give the crude alkyl iodide which was used directly in the next step without further purification.

To a solution of 1,3-dithiane (1.48 g, 12.3 mmol) in a mixture (5:1) of THF and HMPA (35 mL) at −20 °C, was added *n*-butyllithium (5.0 mL of a 2.35 M solution in hexanes, 12 mmol) dropwise. The resulting dark yellow solution was stirred at −20 °C for 1 h, before the slow addition of a solution of the crude iodide (dried by azeotropic distillation with benzene four times) in THF (8 mL). The reaction mixture was stirred at −20 °C for 1.5 h, before the slow addition of saturated aqueous ammonium chloride solution (45 mL) and the biphasic mixture was allowed to warm to rt. The phases were separated, and the aqueous phase was extracted with ether (3 × 45 mL). The combined organic extracts were washed with brine (120 mL), dried over magnesium sulfate, filtered and concentrated. Residual material was purified by silica gel chromatography (pet. ether / ethyl acetate, 90:10) to deliver a diastereomeric mixture of the dithiane **9** (1.29 g, 71% over three steps) as a colorless oil. R*_f_* = 0.45 (pet. ether / ethyl acetate, 90:10); ${[\alpha]}_{D}^{24}$ −6.34 (*c* = 1.96, CHCl_3_); ν_max._ 2975, 2933, 2898 cm^−1^; ^1^H NMR (500 MHz, CDCl_3_) δ 4.74 (1H minor, q, *J* = 5.3 Hz), 4.71 (1H major, q, *J* = 5.3 Hz), 4.08 (1H minor, dd, *J* = 9.4, 5.6 Hz), 4.07 (1H major, dd, *J* = 9.3, 5.6 Hz), 3.67−3.55 (2H major + 2H minor, m), 3.49 (1H major, qd, *J* = 7.1, 4.0 Hz), 3.47 (1H minor, qd, *J* = 7.1, 4.0 Hz), 2.94−2.78 (4H major + 4H minor, m), 2.16−2.09 (1H major + 1H minor, m), 2.05−1.96 (1H major + 1H minor, m), 1.92−1.82 (2H minor + 1H major, m), 1.79 (1H major, ddd, *J* = 14.1, 9.3, 4.8 Hz), 1.53 (1H major, ddd, *J* = 14.1, 9.2, 5.6 Hz), 1.52 (1H minor, ddd, *J* = 14.2, 9.2, 5.6 Hz), 1.30 (3H major, d, *J* = 5.3 Hz), 1.29 (3H minor, d, *J* = 5.3 Hz), 1.20 (3H major, t, *J* = 7.1 Hz), 1.19 (3H minor, t, *J* = 7.1 Hz), 1.11 (3H major, d, *J* = 6.4 Hz), 1.06 (3H minor, d, *J* = 6.3 Hz), 0.95 (3H minor, d, *J* = 6.8 Hz), 0.92 (3H major, d, *J* = 6.8 Hz); ^13^C NMR (126 MHz, CDCl_3_) δ 99.6, 97.9, 76.2, 74.3, 60.10, 60.07, 45.9, 45.8, 38.6, 38.5, 35.4, 34.6, 30.7, 30.40, 30.39, 26.3, 26.2, 20.84, 20.82, 16.8, 16.1, 15.5, 15.0, 14.4; HRMS (ESI+) *m*/*z*: [M+Na]^+^ calcd for C_13_H_26_O_2_S_2_Na 301.1266, found 301.1258.

**(*R*)-*tert*-Butyl[(2-methylbut-3-yn-1-yl)oxy]diphenylsilane (11).**

*Note: Vinylic iodides are generally light-sensitive and so all manipulations were performed under low-light conditions.*

To a solution of zirconocene dichloride (1.01 g, 3.46 mmol) in 1,2-dichloroethane (13 mL) at rt, was added trimethylaluminium (4.6 mL of 2.0 M solution in hexanes, 9.2 mmol) dropwise. The resulting yellow solution was stirred at rt for 30 min, before the dropwise addition of a solution of alkyne **10**^3^ (740 mg, 2.29 mmol) in 1,2-dichloroethane (6 mL). The reaction mixture was stirred at rt for 24 h and then cooled to −30 °C. A solution of iodine (1.17 g, 4.61 mmol) in THF (10 mL) was added dropwise, until the red-brown color remained. The solution was stirred at −30 °C for 30 min, before the dropwise addition of saturated aqueous potassium sodium tartrate solution (50 mL) and dichloromethane (25 mL). The biphasic mixture was stirred vigorously at rt for 1 h and the phases were separated. The aqueous phase was extracted with ether (3 × 75 mL) and the combined organic extracts were washed with brine (250 mL), dried over magnesium sulfate, filtered and concentrated. Residual material was purified by silica gel chromatography (pet. ether / ether, 100:1) to afford the vinyl iodide **11** (1.03 g, 97%) as a colorless oil. R*_f_* = 0.32 (pet. ether); ${[\alpha]}_{D}^{25}$ +9.3 (*c* = 0.90, CHCl_3_); ν_max._ 3071, 3050, 2961, 2930, 2895, 2859, 939, 824, 739, 700 cm^−1^; ^1^H NMR (400 MHz, CDCl_3_) δ 7.68−7.61 (4H, m), 7.46−7.35 (6H, m), 5.96 (1H, app p, *J* = 1.1 Hz), 3.56 (1H, dd, *J* = 10.0, 7.0 Hz), 3.53 (1H, dd, *J* = 10.0, 6.2 Hz), 2.65−2.54 (1H, m), 1.70 (3H, d, *J* = 1.1 Hz), 1.04 (9H, s), 1.01 (3H, d, *J* = 6.9 Hz); ^13^C NMR (126 MHz, CDCl_3_) δ 149.7, 135.79, 135.76, 133.9, 133.8, 129.77, 129.76, 127.82, 127.81, 76.6, 66.8, 45.7, 27.0, 21.7, 19.4, 15.7; HRMS (ESI+) *m*/*z*: [M+Na]^+^ calcd for C_22_H_29_IOSiNa 487.0925, found 487.0911.

**(*R*,*E*)-4-Iodo-2,3-dimethylbut-3-en-1-ol (12).**

To a solution of the silyl ether **11** (1.03 g, 2.22 mmol) in THF (22 mL) at 0 °C, was added tetra-*n*-butylammonium fluoride (3.3 mL of a 1.0 M solution in THF, 3.3 mmol) dropwise. The resulting solution was stirred at rt for 1 h, before the addition of a mixture (1:1) of saturated aqueous ammonium chloride solution and water (20 mL). The phases were separated, and the aqueous phase was extracted with ethyl acetate (3 × 20 mL). The combined organic extracts were washed with brine (60 mL), dried over magnesium sulfate, filtered and concentrated. Residual material was purified by silica gel chromatography (pet. ether / ethyl acetate, 80:20) to afford alcohol **12** (462 mg, 92%) as a colorless oil. R*_f_* = 0.34 (pet. ether / ethyl acetate, 80:20); ${[\alpha]}_{D}^{24}$ +12.0 (*c* = 5.38, CHCl_3_); ν_max._ 3329, 2963, 2930, 2874, 1613, 972, 897, 775, 681 cm^−1^; ^1^H NMR (500 MHz, CDCl_3_) δ 6.08−6.05 (1H, m), 3.57−3.48 (2H, m), 2.66−2.58 (1H, m), 1.81 (3H, d, *J* = 1.1 Hz), 1.35−1.27 (1H, m), 1.04 (3H, d, *J* = 6.9 Hz); ^13^C NMR (126 MHz, CDCl_3_) δ 149.1, 77.1, 65.5, 46.1, 21.2, 15.6; HRMS (ESI+) *m*/*z*: [M+Na]^+^ calcd for C_6_H_11_IONa 248.9747, found 248.9755.

**(*R*,*E*)-4-Iodo-2,3-dimethylbut-3-enal (13).**

*Note: Aldehyde* ***13*** *was prone to decomposition and was therefore used in the next step immediately.*

To a solution of alcohol **12** (70 mg, 0.31 mmol) in dichloromethane (3 mL) at rt, were added sodium bicarbonate (166 mg, 1.98 mmol) and Dess-Martin periodinane (210 mg, 0.495 mmol) sequentially. The resulting mixture was stirred at rt for 1.5 h, before the addition of a mixture (1:1) of saturated aqueous sodium sulfite solution and saturated aqueous sodium bicarbonate solution (6 mL). The biphasic mixture was stirred vigorously for 10 min and the phases were separated. The aqueous phase was extracted with ether (3 × 6 mL) and the combined organic extracts were dried over magnesium sulfate, filtered and concentrated. Residual material was used directly in the next step without further purification.

**2-((2*S*,3*R*,5*R*)-3-Methyl-5-{(5*S*,6*S*)-2,2,3,3,8,8,9,9-octamethyl-6-[1-(tri-*n*-butylstannyl)vinyl]-4,7-dioxa-3,8-disiladecan-5-yl}tetrahydrofuran-2-yl)ethanol (15).**

To a solution of pivalate **14**^4^ (23 mg, 28 μmol) in ether (2.3 mL) at −78 °C, was added diisobutyaluminium hydride (0.11 mL of a 1.0 M solution in hexanes, 0.11 mmol) dropwise. The resulting solution was stirred at −78 °C for 30 min. The reaction was quenched by the dropwise addition of saturated aqueous potassium sodium tartrate solution (3 mL) and the biphasic mixture was stirred vigorously at rt for 30 min. The phases were separated, and the aqueous phase was extracted with ether (3 × 3 mL). The combined organic extracts were dried over sodium sulfate, filtered and concentrated. Residual material was purified by silica gel chromatography (CH_2_Cl_2_ / ethyl acetate, 99:1) to give alcohol **15** (20 mg, 97%) as a colorless oil. R*_f_ =* 0.36 (hexane / ethyl acetate, 90:10); ${[\alpha]}_{D}^{21}$ −16.4 (*c* = 0.950, CHCl_3_); ν_max._ 3393, 2955, 2928, 2897, 2857, 991, 959, 928, 885, 833, 814, 777 cm^−1^; ^1^H NMR (500 MHz, CDCl_3_) δ 5.96 (1H, dd, *J* = 2.9, 1.9 Hz, ^3^*J*_SnH_ = 132.3 Hz), 5.25 (1H, dd, *J* = 2.9, 1.9 Hz, ^3^*J*_SnH_ = 64.0 Hz), 4.33 (1H, app dt, *J* = 2.6, 1.9 Hz, ^3^*J*_SnH_ = 29.7 Hz), 4.16 (1H, ddd, *J* = 7.3 Hz), 3.96 (1H, ddd, *J* = 10.5, 5.3, 3.0 Hz), 3.82−3.72 (2H, m), 3.54 (1H, dd, *J* = 7.2, 2.6 Hz), 2.65 (1H, dd, *J* = 7.8, 3.3 Hz), 2.21−2.11 (1H, m), 1.82 (1H, ddd, *J* = 12.7, 8.1, 7.3 Hz), 1.70 (1H, dddd, *J* = 13.7, 10.5, 8.3, 5.1 Hz), 1.58−1.40 (8H, m), 1.32 (6H, m), 0.91 (9H, s), 0.90 (9H, s), 0.96−0.86 (18H, m), 0.11 (3H, s), 0.09 (3H, s), 0.08 (3H, s), 0.00 (3H, s); ^13^C NMR (126 MHz, CDCl_3_) δ 153.9, 125.9, 82.7, 80.8, 78.9, 78.7, 62.4, 37.2, 36.7, 33.0, 29.3 (^1^*J*^119^_SnC_ = 19 Hz, ^1^*J*^117^_SnC_ = 19 Hz), 27.7 (^1^*J*^119^_SnC_ = 61 Hz, ^1^*J*^117^_SnC_ = 59 Hz), 26.4, 26.3, 18.64, 18.62, 14.2, 13.9, 10.3 (^1^*J*^119^_SnC_ = 336 Hz, ^1^*J*^117^_SnC_ = 321 Hz), −3.6, −3.9, −4.1, −4.3; HRMS (ESI+) *m*/*z*: [M+Na]^+^ calcd for C_35_H_74_O_4_Si_2_SnNa 757.4040, found 757.4018.

**2-((2*S*,3*R*,5*R*)-3-Methyl-5-{(5*S*,6*S*)-2,2,3,3,8,8,9,9-octamethyl-6-[1-(tri-*n*-butylstannyl)vinyl]-4,7-dioxa-3,8-disiladecan-5-yl}tetrahydrofuran-2-yl)acetaldehyde (16).**

To a solution of alcohol **15** (19 mg, 26 μmol) in dichloromethane (1.7 mL) at rt, was added Dess-Martin periodinane (22 mg, 52 μmol). The resulting solution was stirred at rt for 2 h. A further portion Dess-Martin periodinane (22 mg, 52 μmol) was added and the reaction mixture was stirred for another 2 h. The reaction was quenched by the addition of a mixture (1:1) of saturated aqueous sodium sulfite solution and saturated aqueous bicarbonate solution (3 mL). The biphasic mixture was stirred vigorously until the phases became clear (20 min). The phases were separated, and the aqueous phase was extracted with dichloromethane (3 × 3 mL). The combined organic extracts were dried over sodium sulfate, filtered and concentrated. Residual material was purified by silica gel chromatography (hexane / ethyl acetate, 95:5) to deliver aldehyde **16** (19 mg, quant.) as a colorless oil. R*_f_ =* 0.45 (hexane / ethyl acetate, 95:5); ^1^H NMR (500 MHz, CDCl_3_) δ 9.81 (1H, dd, *J* = 2.5, 2.0 Hz), 5.94 (1H, dd, *J* = 2.8, 1.9 Hz, ^3^*J*_SnH_ = 131.7 Hz), 5.24 (1H, dd, *J* = 2.8, 1.9 Hz, ^3^*J*_SnH_ = 63.7 Hz), 4.32 (1H, app dt, *J* = 2.4, 1.9 Hz, ^3^*J*_SnH_ = 29.0 Hz), 4.25 (1H, app dt, *J* = 8.8, 4.9 Hz), 4.13 (1H, ddd, *J* = 9.1, 7.3, 6.6 Hz), 3.54 (1H, dd, *J* = 7.3, 2.4 Hz), 2.59 (1H, ddd, *J* = 16.0, 8.8, 2.5 Hz), 2.41 (1H, ddd, *J* = 16.0, 4.9, 2.0 Hz), 2.31−2.23 (1H, m), 1.78 (1H, ddd, *J* = 12.9, 9.1, 6.9 Hz), 1.56 (1H, ddd, *J* = 12.9, 6.6, 2.4 Hz), 1.53−1.42 (6H, m), 1.32 (6H, h, *J* = 7.3 Hz), 0.96−0.85 (36H, m), 0.08 (3H, s), 0.07 (3H, s), 0.06 (3H, s), −0.01 (3H, s).

**2-((2*S*,3*R*,5*R*)-3-Methyl-5-{(5*S*,6*S*)-2,2,3,3,8,8,9,9-octamethyl-6-[1-(tri-*n*-butylstannyl)vinyl]-4,7-dioxa-3,8-disiladecan-5-yl}tetrahydrofuran-2-yl)acetic acid (17).**^5^

To a vigorously stirred solution of aldehyde **16** (19 mg, 26 µmol) in a mixture (2:1) of *t*-butanol and water (3.7 mL) at 0 °C, were added 2-methyl-2-butene (0.14 mL, 1.3 mmol), sodium dihydrogen phosphate dihydrate (40 mg, 0.26 mmol) and sodium chlorite (12 mg, 0.13 mmol) sequentially. The resulting mixture was stirred at 0 °C for 15 min and at rt for 1 h. The reaction mixture was diluted with dichloromethane (4 mL) and water (4 mL) was added. The phases were separated, and the aqueous phase was extracted with dichloromethane (3 × 4 mL). The combined organic extracts were dried over sodium sulfate, filtered and concentrated. Residual material was purified by silica gel chromatography (hexane / ethyl acetate, 90:10) to give acid **17** (17 mg, 88% over two steps) as a colorless oil. R*_f_* = 0.21 (hexane / ethyl acetate, 90:10); ${[\alpha]}_{D}^{19}$ −18.4 (*c* = 0.800, CHCl_3_) {lit.^5^ ${[\alpha]}_{D}^{20}$ −12.8 (*c* = 1.32, CHCl_3_)}; ν_max._ 2957, 2926, 2855, 1713, 959, 928, 878, 831, 802, 775 cm^−1^; ^1^H NMR (500 MHz, CDCl_3_) δ 10.62 (1H, br s), 5.96 (1H, dd, *J* = 2.5, 1.5 Hz, ^3^*J*_SnH_ = 131.6 Hz), 5.26 (1H, dd, *J* = 2.5, 1.5 Hz, ^3^*J*_SnH_ = 63.7 Hz), 4.35−4.33 (1H, m, ^3^*J*_SnH_ = 28.6 Hz), 4.24−4.14 (2H, m), 3.55 (1H, dd, *J* = 7.1, 2.2 Hz), 2.52 (1H, dd, *J* = 15.9, 8.1 Hz), 2.45 (1H, dd, *J* = 15.9, 5.4 Hz), 2.34−2.25 (1H, m), 1.87−1.80 (1H, m), 1.57 (1H, ddd, J = 13.0, 6.8, 3.2 Hz), 1.53−1.42 (6H, m), 1.32 (6H, h, *J* = 7.2 Hz), 0.96−0.87 (36H, m), 0.08 (3H, s), 0.08 (3H, s), 0.07 (3H, s), 0.00 (3H, s); ^13^C NMR (126 MHz, CDCl_3_) δ 174.7, 153.9, 126.0, 82.6 (^1^*J*^119^_SnC_ = 57 Hz, ^1^*J*^117^_SnC_ = 56 Hz), 79.1, 78.5, 76.6, 37.3, 36.2, 29.3 (^1^*J*^119^_SnC_ = 19 Hz, ^1^*J*^117^_SnC_ = 19 Hz), 27.6 (^1^*J*^119^_SnC_ = 61 Hz, ^1^*J*^117^_SnC_ = 59 Hz), 26.5, 26.3, 18.7, 18.6, 14.0, 13.8, 10.3 (^1^*J*^119^_SnC_ = 336 Hz, ^1^*J*^117^_SnC_ = 321 Hz), −3.7, −3.9, −4.2, −4.3; HRMS (ESI+) *m*/*z*: [M+Na]^+^ calcd for C_35_H_72_O_5_Si_2_SnNa 771.3832, found 771.3808.

***tert*-Butyl({(*R*,*E*)-1-[(2*R*,5*R*)-5-(iodomethyl)tetrahydrofuran-2-yl]-5-methylhexa-2,4-dien-1-yl}-oxy)dimethylsilane (S3)**

*Note: The alkyl iodide product is light-sensitive and all manipulations were performed under low-light conditions.*

To a solution of alcohol **6** (247 mg, 0.756 mmol) in THF (7.6 mL) at 0 °C, were added triphenylphosphine (337 mg, 1.29 mmol) and imidazole (154 mg, 2.27 mmol) sequentially. The resulting mixture was stirred at 0 °C for 10 min, before the portion-wise addition of iodine (288 mg, 1.13 mmol). The brown solution was stirred at 0 °C for 10 min and at rt for 2 h. The reaction was quenched by the addition of saturated aqueous sodium sulfite solution (8 mL) and the phases were separated. The aqueous phase was extracted with ether (3 × 8 mL) and the combined organic extracts were washed with brine (30 mL), dried over magnesium sulfate, filtered and concentrated. The residue was filtered rapidly through a short pad of silica gel (pet. ether / ethyl acetate, 95:5) to give the crude alkyl iodide **S3** which was used directly in the next step without further purification. R*_f_ =* 0.32 (pet. ether / ethyl acetate, 98:2); ^1^H NMR (500 MHz, CDCl_3_) δ 6.47 (1H, ddd, *J* = 15.2, 11.1, 1.4 Hz), 5.83 (1H, br d, *J* = 11.1 Hz), 5.53 (1H, dd, *J* = 15.2, 5.6 Hz), 4.22 (1H, app td, *J* = 5.6, 1.4 Hz), 4.08 (1H, app td, *J* = 7.1, 5.6 Hz), 4.03 (1H, app tdd, *J* = 7.3, 6.2, 4.8 Hz), 3.25 (1H, dd, *J* = 9.8, 4.8 Hz), 3.16 (1H, dd, *J* = 9.8, 7.3 Hz), 2.11 (1H, dddd, *J* = 12.3, 8.5, 6.2, 4.1 Hz), 1.95 (1H, dddd, *J* = 12.6, 8.5, 7.1, 4.1 Hz), 1.80 (1H, app dtd, *J* = 12.6, 8.5, 7.1 Hz), 1.78 (3H, br s), 1.75 (3H, br s), 1.62 (1H, app dtd, *J* = 12.3, 8.5, 7.3 Hz), 0.90 (9H, s), 0.07 (3H, s), 0.05 (3H, s).

***tert*-Butyl[((*R*,*E*)-1-{(2*R*,5*R*)-5-[(2-{(2*S*,3*R*)-3-[(1(*R*/*S*)-ethoxy)ethoxy]-2-methylbutyl}-1,3-dithian-2-yl)methyl]tetrahydrofuran-2-yl}-5-methylhexa-2,4-dien-1-yl)oxy]dimethylsilane (18).**

To a solution of dithiane **9** (dried by azeotropic distillation with benzene four times, 421 mg, 1.51 mmol) in a mixture of THF and HMPA (9:1, 15 mL) at −78 °C, was added *tert*-butyllithium (820 μL of a 1.85 M solution in pentane, 1.51 mmol) dropwise. The resulting dark orange solution was stirred at −78 °C for 10 min, before the slow addition of a precooled solution of the crude alkyl iodide **S3** (dried by azeotropic distillation with benzene four times) in THF (2.5 mL). The dark green / black reaction mixture was stirred at −78 °C for 45 min, before the slow addition of saturated aqueous ammonium chloride solution (18 mL) and the biphasic mixture was allowed to warm to rt. The phases were separated, and the aqueous phase was extracted with ether (3 × 20 mL). The combined organic extracts were washed with brine (60 mL), dried over magnesium sulfate, filtered and concentrated. The residue was filtered through a short pad of silica gel (pet. ether / ethyl acetate, 9:1) to deliver the crude product (450 mg), which consisted of a mixture (~1:2) of the acetal **18** and excess dithiane **9** as a colorless oil. An analytical sample of the desired product was obtained by treatment with tetra-*n*-butylammonium fluoride in THF at rt and purification of the resulting alcohol by silica-gel chromatography to remove dithiane **9**, followed by a TBS reprotection. However, the crude mixture was generally used directly in the next step without further purification. R*_f_ =* 0.42 (pet. ether / ethyl acetate, 9:1); ν_max._ 2957, 2928, 2909, 2857, 1659, 961, 910, 860, 835, 810, 775 cm^−1^; ^1^H NMR (500 MHz, CDCl_3_) δ 6.45 (1H, ddd, *J* = 15.1, 11.0, 1.5 Hz), 6.44 (1H, ddd,*J* = 15.1, 11.1, 1.4 Hz), 5.83 (2H, br d, *J* = 11.0 Hz), 5.57 (2H, dd, *J* = 15.1, 5.7 Hz),4.79 (1H, q, *J* = 5.3 Hz), 4.75 (1H, q, *J* = 5.3 Hz), 4.23−4.18 (2H, m), 4.17−4.10 (2H, m), 4.00−3.92 (2H, m), 3.75−3.62 (4H, m), 3.54−3.44 (2H, m), 2.94−2.69 (8H, m), 2.29−2.25 (1H, m), 2.24 (1H, dd, *J* = 8.3, 5.5 Hz), 2.20−2.07 (6H, m), 2.02 (1H, dd, *J* = 15.1, 3.5 Hz), 2.02−1.91 (2H, m), 1.92−1.83 (5H, m), 1.77 (6H, br s), 1.75 (6H, br s), 1.76−1.70 (2H, m), 1.67 (2H, dd, *J* = 15.1, 5.8 Hz), 1.55−1.46 (2H, m), 1.31 (3H, d, *J* = 7.4 Hz), 1.30 (3H, d, *J* = 7.5 Hz), 1.20 (3H, t, *J* = 7.0 Hz), 1.20 (3H, t, *J* = 7.1 Hz), 1.11 (3H, d, *J* = 6.4 Hz), 1.06 (3H, d, *J* = 6.2 Hz), 1.05 (3H, d, *J*= 6.7 Hz), 1.02 (3H, d, *J* = 6.9 Hz), 0.90 (18H, s), 0.06 (6H, s), 0.04 (6H, s); ^13^C NMR (126 MHz, CDCl_3_) δ 134.91, 134.86, 130.2, 127.49, 127.47, 124.94, 124.92, 99.1, 98.3, 81.83, 81.77, 76.78, 76.77, 75.53, 75.47, 75.4, 60.7, 60.5, 53.5, 53.3, 45.0, 42.8, 34.5, 34.23, 34.19, 33.4, 29.9, 27.3, 27.2, 26.5, 26.49, 26.48, 26.13, 26.05, 25.2, 21.2, 21.0, 18.4, 16.9, 16.0, 15.8, 15.6, 15.55, 15.51, −4.3, −4.3, −4.6; HRMS (ESI+) *m*/*z*: [M+Na]^+^ calcd for C_31_H_58_O_4_S_2_SiNa 609.3438, found 609.3413.

**(2*R*,3*S*)-4-(2-{(2*R*,5*R*)-5-[(*R*,*E*)-1-(*tert*-Butyldimethylsilyloxy)-5-methylhexa-2,4-dien-1-yl]-tetrahydrofuran-2-yl}methyl-1,3-dithian-2-yl)-3-methylbutan-2-ol (19).**

To a solution of the crude **18** in a mixture of ethanol and dichloromethane (3:1, 59 mL) at rt, was added PPTS (45 mg, 0.18 mmol). The resulting solution was stirred at rt for 9 h, before the addition of triethylamine (75 μL, 0.54 mmol) and the mixture was concentrated. Residual material was purified by silica gel chromatography (pet. ether / ethyl acetate, 85:15) to deliver alcohol **19** (171 mg, 44% over three steps) as a colorless oil. R*_f_ =* 0.20 (pet. ether / ethyl acetate, 85:15); ${[\alpha]}_{D}^{19}$ +16.3 (*c* = 0.490, CHCl_3_); ν_max._ 3435, 2955, 2928, 2907, 2857, 1661, 988, 961, 939, 908, 862, 835, 812, 775 cm^−1^; ^1^H NMR (500 MHz, CDCl_3_) δ 6.45 (1H, ddd, *J* = 15.2, 11.0, 1.4 Hz), 5.83 (1H, br d, *J* = 11.0 Hz), 5.56 (1H, dd, *J* = 15.2, 5.6 Hz), 4.20 (1H, app td, *J* = 5.6, 1.4 Hz), 4.16 (1H, app dq, *J* = 9.0, 5.3 Hz), 3.97 (1H, app td, *J* = 7.3, 5.6 Hz), 3.67 (1H, app pd *J* = 6.1, 5.5 Hz), 2.91−2.71 (4H, m), 2.24 (1H, dd, *J* = 15.0, 5.3 Hz), 2.18 (1H, dd, *J* = 15.0, 5.3 Hz), 2.17 (1H, dd, *J* = 15.0, 2.6 Hz), 2.10 (1H, dddd, *J* = 11.8, 7.9, 5.3, 2.6 Hz), 2.00−1.82 (4H, m), 1.77 (3H, br s), 1.75 (3H, br s), 1.76−1.69 (1H, m), 1.67 (1H, dd, *J* = 15.0, 6.2 Hz), 1.55 (1H, d, *J* = 5.5 Hz), 1.56−1.47 (1H, m), 1.15 (3H, d, *J* = 6.1 Hz), 1.05 (3H, d, *J* = 6.9 Hz), 0.90 (9H, s), 0.06 (3H, s), 0.04 (3H, s); ^13^C NMR (126 MHz, CDCl_3_) δ 135.0, 130.2, 127.5, 124.9, 81.9, 76.8, 75.5, 72.5, 53.2, 45.4, 43.0, 37.0, 34.3, 27.3, 26.5, 26.4, 26.1, 26.0, 25.1, 19.7, 18.4, 17.9, −4.3, −4.6; HRMS (ESI+) *m*/*z*: [M+Na]^+^ calcd for C_27_H_50_O_3_S_2_SiNa 537.2863, found 537.2838.

**(*S*)-4-(2-{(2*R*,5*R*)-5-[(*R*,*E*)-1-(*tert*-Butyldimethylsilyloxy)-5-methylhexa-2,4-dien-1-yl]tetra-hydrofuran-2-yl}methyl-1,3-dithian-2-yl)-3-methylbutan-2-one (20).**

To a solution of alcohol **19** (171 mg, 0.332 mmol) in dichloromethane (3.3 mL) at 0 °C, were added DMSO (0.33 mL, 4.6 mmol), triethylamine (0.32 mL, 2.3 mmol) and SO_3_-pyridine complex (291 mg, 1.83 mmol) sequentially. The resulting mixture was stirred at 0 °C for 24 h, after which more DMSO (0.17 mL, 2.4 mmol), triethylamine (0.16 mL, 1.1 mmol) and SO_3_-pyridine complex (145 mg, 0.911 mmol) were added. The reaction mixture was stirred at 0 °C for another 24 h, before the addition of a saturated aqueous ammonium chloride solution (4 mL) and the layers were separated. The aqueous phase was extracted with ether (3 × 5 mL) and the combined organic extracts were washed with brine (15 mL), dried over sodium sulphate, filtered and concentrated. Residual material was purified by silica gel chromatography (pet. ether / ethyl acetate, 95:5) to deliver ketone **20** (133 mg, 78%) as a colorless oil. R*_f_ =* 0.23 (pet. ether / ethyl acetate, 95:5); ${[\alpha]}_{D}^{25}$ +26.1 (*c* = 1.52, CHCl_3_); ν_max._ 2955, 2928, 2909, 2857, 1711, 1661, 988, 961, 939, 909, 858, 835, 812, 775 cm^−1^; ^1^H NMR (500 MHz, CDCl_3_) δ 6.43 (1H, ddd, *J* = 15.2, 11.0, 1.4 Hz), 5.82 (1H, br d, *J* = 11.0 Hz), 5.55 (1H, dd, *J* = 15.2, 5.7 Hz), 4.24−4.17 (2H, m), 3.99 (1H, app td, *J* = 7.3, 4.9 Hz), 3.03 (1H, ddd, *J* = 14.5, 9.1, 0.7 Hz), 3.00 (1H, ddd, *J* = 14.3, 11.2, 2.7 Hz), 2.84 (1H, dqd, *J* = 9.1, 7.2, 1.4 Hz), 2.77 (1H, ddd, *J* = 14.3, 11.2, 2.7 Hz), 2.66 (1H, dddd, *J* = 14.3, 5.8, 3.1, 1.0 Hz), 2.48 (1H, dddd, *J* = 14.3, 5.8, 3.1, 1.0 Hz), 2.25 (3H, s), 2.08 (1H, dd, *J* = 15.0, 7.4 Hz), 2.05−1.97 (2H, m), 1.98 (1H, ddd, *J* = 15.0, 3.1, 0.7 Hz), 1.89 (1H, dd, *J* = 14.5, 1.4 Hz), 1.89−1.69 (3H, m), 1.77 (3H, br s), 1.75 (3H, br s), 1.48 (1H, app ddt, *J* = 12.0, 10.0, 8.5 Hz), 1.08 (3H, d, *J* = 7.2 Hz,), 0.90 (9H, s), 0.05 (3H, s), 0.04 (3H, s); ^13^C NMR (126 MHz, CDCl_3_) δ 212.6, 135.0, 130.1, 127.6, 124.8, 82.2, 75.3, 75.1, 51.9, 45.6, 43.6, 40.7, 34.0, 30.1, 26.9, 26.7, 26.13, 26.11, 26.0, 25.1, 18.9, 18.41, 18.40, −4.3, −4.6; HRMS (ESI+) *m*/*z*: [M+Na]^+^ calcd for C_27_H_48_O_3_S_2_SiNa 535.2706, found 535.2680.

**(*S*)-4-(2-{(2*R*,5*R*)-5-[(*R*,*E*)-1-Hydroxy-5-methylhexa-2,4-dien-1-yl]tetrahydrofuran-2-yl}methyl-1,3-dithian-2-yl)-3-methylbutan-2-one (21).**

To a solution of TBS ether **20** (133 mg, 0.259 mmol) in THF (6.5 mL) at 0 °C, was added tetra-*n*-butylammonium fluoride (0.52 mL of a 1.0 M solution in THF, 0.52 mmol) dropwise. The resulting solution was stirred at rt for 5 h. The reaction was quenched by the addition of water (7 mL) and the layers were separated. The aqueous phase was extracted with ethyl acetate (3 × 7 mL) and the combined organic extracts were washed with brine (25 mL), dried over sodium sulfate, filtered and concentrated. Residual material was purified by silica gel chromatography (pet. ether / ethyl acetate, 70:30) to give alcohol **21** (103 mg, 99%) as a colorless oil. R*_f_ =* 0.44 (pet. ether / ethyl acetate, 70:30); ${[\alpha]}_{D}^{26}$ +13.2 (*c* = 1.32, CHCl_3_); ν_max._ 3451, 2963, 2924, 2872, 2857, 1709, 1661, 988, 961, 922, 909, 872, 801 cm^−1^; ^1^H NMR (500 MHz, CDCl_3_) δ 6.52 (1H, ddd, *J* = 15.2, 11.0, 1.2 Hz), 5.81 (1H, d, *J* = 11.0 Hz), 5.45 (1H, dd, *J* = 15.2, 6.8 Hz), 4.29−4.22 (1H, m), 3.97 (1H, dddd, *J* = 7.1, 6.8, 2.9, 1.2 Hz), 3.89 (1H, app q, *J* = 7.1 Hz), 3.09 (1H, dd, *J* = 14.6, 9.4 Hz), 2.98 (1H, ddd, *J* = 14.4, 10.7, 2.8 Hz), 2.88 (1H, dqd, *J* = 9.4, 7.2, 1.5 Hz), 2.77 (1H, ddd, *J* = 14.4, 10.7, 2.8 Hz), 2.68 (1H, dddd, *J* = 14.4, 6.2, 3.1, 0.7 Hz), 2.57 (1H, d, *J* = 2.9 Hz), 2.54 (1H, dddd, *J* = 14.4, 6.2, 3.1, 0.7 Hz), 2.26 (3H, s), 2.14−2.07 (1H, m), 2.10 (1H, dd, *J* = 15.3, 7.7 Hz), 2.03 (1H, dd, *J* = 15.3, 3.0 Hz), 2.04−1.91 (2H, m), 1.88−1.78 (1H, m), 1.80 (1H, dd, *J* = 14.6, 1.5 Hz), 1.77 (3H, br s), 1.76 (3H, br s), 1.70−1.60 (1H, m), 1.61−1.52 (1H, m), 1.08 (3H, d, *J* = 7.2 Hz); ^13^C NMR (126 MHz, CDCl_3_) δ 212.2, 136.6, 129.4, 128.2, 124.5, 82.5, 75.5, 75.0, 52.0, 45.3, 43.5, 41.3, 34.1, 29.9, 27.8, 26.9, 26.2, 26.1, 25.0, 19.1, 18.5; HRMS (ESI+) *m*/*z*: [M+Na]^+^ calcd for C_21_H_34_O_3_S_2_Na 421.1842, found 421.1826.

**(*S*)-3-Methyl-4-(2-{(2*R*,5*R*)-5-[(*R*,*E*)-5-methyl-1-(triethylsilyloxy)hexa-2,4-dien-1-yl]tetrahydro-furan-2-yl}methyl-1,3-dithian-2-yl)butan-2-one (22).**

To a solution of alcohol **21** (103 mg, 0.258 mmol) in dichloromethane (6.5 mL) at −78 °C, was added 2,6-lutidine (90 μL, 0.78 mmol) and, after 5 min, triethylsilyl trifluoromethanesulfonate (88 μL, 0.39 mmol). The resulting solution was stirred at −78 °C for 45 min, before the addition of water (7 mL) and the biphasic mixture was allowed to warm to rt. The phases were separated, and the aqueous phase was extracted with ether (3 × 7 mL). The combined organic extracts were washed with brine (25 mL), dried over sodium sulfate, filtered and concentrated. Residual material was purified by silica gel chromatography (pet. ether / ethyl acetate, 95:5) to deliver triethylsilyl ether **22** (118 mg, 89%) as a colorless oil. R*_f_ =* 0.28 (pet. ether / ethyl acetate, 95:5); ${[\alpha]}_{D}^{25}$ +26.8 (*c* = 1.00, CHCl_3_); ν_max._ 2957, 2932, 2911, 2876, 1711, 1659, 986, 961, 909, 874, 843, 816, 799, 741, 727 cm^−1^; ^1^H NMR (500 MHz, CDCl_3_) δ 6.43 (1H, ddd, *J* = 15.2, 11.0, 1.3 Hz), 5.82 (1H, br d, *J* = 11.0 Hz), 5.53 (1H, dd, *J* = 15.2, 6.1 Hz), 4.23−4.17 (2H, m), 3.98 (1H, ddd, *J* = 7.6, 7.4, 5.3 Hz), 3.03 (1H, ddd, *J* = 14.5, 9.0, 1.0 Hz), 3.01 (1H, ddd, *J* = 14.2, 11.0, 2.7 Hz), 2.84 (1H, dqd, *J* = 9.0, 7.1, 1.3 Hz), 2.77 (1H, ddd, *J* = 14.2, 11.3, 2.8 Hz), 2.66 (1H, dddd, *J* = 14.2, 5.4, 3.1, 0.9 Hz), 2.47 (1H, dddd, *J* = 14.2, 5.8, 3.1, 0.9 Hz), 2.25 (3H, s), 2.09 (1H, dd, *J* = 15.0, 7.4 Hz), 2.06−1.98 (2H, m), 1.97 (1H, ddd, *J* = 15.0, 3.3, 1.0 Hz), 1.90 (1H, dd, *J* = 14.5, 1.3 Hz), 1.88−1.78 (2H, m), 1.77 (3H, br s), 1.75 (3H, br s), 1.70 (1H, dddd, *J* = 12.6, 10.1, 7.9, 7.6 Hz), 1.48 (1H, app ddt, *J* = 12.0, 10.1, 8.4 Hz), 1.09 (3H, d, *J* = 7.1 Hz), 0.95 (9H, t, *J* = 7.9 Hz), 0.59 (6H, q, *J* = 7.9 Hz); ^13^C NMR (126 MHz, CDCl_3_) δ 212.6, 135.2, 130.1, 127.8, 124.8, 82.4, 75.6, 75.0, 51.9, 45.6, 43.7, 40.6, 34.1, 30.1, 27.1, 26.9, 26.14, 26.11, 25.1, 18.9, 18.4, 7.1, 5.2; HRMS (ESI+) *m*/*z*: [M+Na]^+^ calcd for C_27_H_48_O_3_S_2_SiNa 535.2706, found 535.2690.

**(2*S*,5*R*,6*R*,*E*)-5-Hydroxy-8-iodo-2,6,7-trimethyl-1-(2-{(2*R*,5*R*)-5-[(*R*,*E*)-5-methyl-1-(triethylsilyl-oxy)hexa-2,4-dien-1-yl]tetrahydrofuran-2-yl}methyl-1,3-dithian-2-yl)oct-7-en-3-one (23a) and (2*S*,5*S*,6*R*,*E*)-5-Hydroxy-8-iodo-2,6,7-trimethyl-1-(2-{(2*R*,5*R*)-5-[(*R*,*E*)-5-methyl-1-(triethylsilyl-oxy)hexa-2,4-dien-1-yl]tetrahydrofuran-2-yl}methyl-1,3-dithian-2-yl)oct-7-en-3-one (23b).**

*Note: Reactions were performed on three equivalent batches and the crude products were combined for purification.*

To a solution of ketone **22** (dried by azeotropic distillation with benzene four times, 46 mg, 90 µmol) in ether (900 µL) at 0 °C, were added triethylamine (20 µL, 0.14 mmol) and chloro(dicyclohexyl)borane (29 µL, 0.14 mmol) sequentially. The resulting mixture was stirred at 0 °C for 1 h (a white precipitate formed) and then cooled to −78 °C. A solution of crude aldehyde **13** (dried by azeotropic distillation with benzene four times) in ether (450 µL) was added and the mixture was stirred at −78 °C for 1.5 h. Aqueous pH 7 buffer (3 mL) was added and the resulting biphasic mixture was stirred at 0 °C for 30 min. The phases were separated, and the aqueous phase was extracted with ether (3 × 4 mL). The combined organic extracts were dried over sodium sulfate, filtered and concentrated. A diastereomeric mixture of the aldol products **23a** and **23b** (2.2:1) was observed by ^1^H NMR analysis of the crude product mixture.

Three batches of the material (form a combined total of 138 mg of ketone **22**) were combined and purified by silica gel chromatography (pet. ether / CH_2_Cl_2_ / Et_2_O, 49:49:2) to afford the β-hydroxyketone **23a** (110 mg, 55%) along with the less polar diastereomer **23b** (20 mg, 10%) as colorless oils. The configuration at the hydroxyl-bearing stereogenic centre in the products was determined by esterification of the alcohol **23a** with both enantiomers of Mosher’s acid and ^1^H NMR analysis of the diastereomeric esters (**S4** and **S5**) according to the procedure of Hoye and co-workers.^6^ **23a**: R*_f_* = 0.24 (pet. ether / CH_2_Cl_2_ / Et_2_O, 49:49:2, eluted twice); ${[\alpha]}_{D}^{30}$ +24.5 (*c* = 2.00, CHCl_3_); ν_max._ 3499, 2957, 2928, 2913, 2874, 1703, 1693, 1659, 988, 963, 909, 873, 839, 800, 743, 727 cm^−1^; ^1^H NMR (500 MHz, CDCl_3_) δ 6.42 (1H, ddd, *J* = 15.3, 11.0, 1.4 Hz), 6.03 (1H, br s), 5.81 (1H, br d, *J* = 11.0 Hz), 5.52 (1H, dd, *J* = 15.3, 6.1 Hz), 4.22−4.16 (2H, m), 4.01−3.96 (1H, m), 3.86 (1H, dddd, *J* = 9.0, 8.1, 3.2, 2.2 Hz), 3.37 (1H, d, *J* = 3.2 Hz), 3.04−2.95 (2H, m), 2.86 (1H, dd, *J* = 18.1, 2.2 Hz), 2.83−2.77 (1H, m), 2.76−2.63 (2H, m), 2.49 (1H, dd, *J* = 18.1, 9.0 Hz), 2.49−2.42 (2H, m), 2.05 (1H, dd, *J* = 15.1, 7.5 Hz), 2.05−1.97 (3H, m), 1.95 (1H, dd, *J* = 14.6, 1.2 Hz), 1.90−1.78 (2H, m), 1.77 (3H, br s), 1.75 (3H, br s), 1.75 (3H, br s), 1.70 (1H, m), 1.48 (1H, app ddt, *J* = 12.0, 10.2, 8.6 Hz), 1.13 (3H, d, *J* = 6.8 Hz), 1.06 (3H, d, *J* = 7.1 Hz), 0.95 (9H, t, *J* = 8.0 Hz), 0.59 (6H, q, *J* = 8.0 Hz); ^13^C NMR (126 MHz, CDCl_3_) δ 216.2, 149.9, 135.3, 130.0, 127.8, 124.8, 82.5, 76.9, 75.6, 74.9, 70.1, 51.8, 49.0, 47.3, 45.6, 43.6, 41.1, 34.0, 27.03, 26.99, 26.2, 26.1, 25.0, 21.5, 18.9, 18.4, 15.5, 7.1, 5.2; HRMS (ESI+) *m*/*z*: [M+Na]^+^ calcd for C_33_H_57_IO_4_S_2_SiNa 759.2404, found 759.2383. **23b**: R*_f_* = 0.29 (pet. ether / CH_2_Cl_2_ / ether, 49:49:2, eluted twice); ν_max._ 3501, 2959, 2930, 2909, 2874, 1697, 1659, 1613, 988, 963, 909, 874, 839, 816, 797, 741, 727 cm^−1^; ^1^H NMR (500 MHz, CDCl_3_) δ 6.42 (1H, ddd, *J* = 15.2, 11.0, 1.4 Hz), 6.01 (1H, br s), 5.81 (1H, br d, *J* = 11.0 Hz), 5.52 (1H, dd, *J* = 15.2, 6.2 Hz), 4.23−4.15 (2H, m), 4.01−3.93 (2H, m), 3.15 (1H, d, *J* = 3.1 Hz), 3.03 (1H, dd, *J* = 14.5, 9.5 Hz), 2.98 (1H, ddd, *J* = 14.0, 10.9, 2.7 Hz), 2.86−2.79 (1H, m), 2.76−2.69 (3H, m), 2.66 (1H, dddd, *J* = 14.0, 5.4, 2.8, 0.7 Hz), 2.51 (1H, app p, *J* = 7.0 Hz), 2.50−2.44 (1H, m), 2.06 (1H, dd, *J* = 15.0, 7.6 Hz), 2.03−1.96 (3H, m), 1.94 (1H, dd, *J* = 14.5, 1.0 Hz), 1.90−1.78 (2H, m), 1.84 (3H, d, *J* = 1.1 Hz), 1.77 (3H, br s), 1.75 (3H, br s), 1.70 (1H, app ddt, *J* = 12.7, 10.1, 7.8 Hz), 1.47 (1H, app ddt, *J* = 12.1, 10.1, 8.5 Hz), 1.09 (3H, d, *J* = 7.2 Hz), 1.03 (3H, d, *J* = 7.0 Hz), 0.94 (9H, t, *J* = 7.9 Hz), 0.59 (6H, q, *J* = 7.9 Hz); ^13^C NMR (126 MHz, CDCl_3_) δ 215.5, 149.8, 135.3, 130.0, 127.8, 124.8, 82.5, 76.9, 75.6, 74.9, 69.8, 51.9, 48.4, 46.0, 45.5, 44.0, 40.4, 34.0, 27.0, 26.8, 26.2, 26.1, 25.0, 21.4, 19.0, 18.4, 15.6, 7.1, 5.2; HRMS (ESI+) *m*/*z*: [M+Na]^+^ calcd for C_33_H_57_IO_4_S_2_SiNa 759.2404, found 759.2369.

| Assignment | **23a** | **S4** | **S5** | Δ (δ**S4** – δ**S5**) |
| --- | --- | --- | --- | --- |
| *1* | 6.03 | 5.940 | 6.017 | –0.077 |
| *2* | 1.75 | 1.778 | 1.873 | –0.095 |
| *3* | 1.13 | 0.990 | 1.048 | –0.058 |
| *4a* | 2.49 | 2.844 | 2.854 | –0.010 |
| *4b* | 2.86 | 3.083 | 3.036 | +0.047 |
| *5* | 1.06 | 0.998 | 0.956 | +0.042 |
| *6* | 1.95 | 1.945 | 1.912 | +0.033 |
| *7* | 2.05 | 2.047 | 2.038 | +0.009 |
| *8* | 3.98 | 3.977 | 3.970 | +0.007 |

**(2*S*,5*R*,6*R*,*E*)-5-(*tert*-Butyldimethylsilyloxy)-8-iodo-2,6,7-trimethyl-1-(2-{(2*R*,5*R*)-5-[(*R*,*E*)-5-methyl-1-(triethylsilyloxy)hexa-2,4-dien-1-yl]tetrahydrofuran-2-yl}methyl-1,3-dithian-2-yl)-oct-7-en-3-one (24a).**

To a solution of β-hydroxy ketone **23a** (43 mg, 58 µmol) in dichloromethane (2.7 mL) at −78 °C, were added 2,6-lutidine (40 µL, 0.35 mmol) and *tert*-butyldimethylsilyl trifluoromethanesulfonate (40 µL, 0.17 mmol) sequentially. The resulting solution was stirred at −78 °C for 5 h, before the addition of water (3 mL) and the biphasic mixture was allowed to warm to rt. The layers were separated, and the aqueous phase was extracted with ether (3 × 3 mL). The combined organic extracts were dried over sodium sulphate, filtered and concentrated. The crude product was purified by silica gel chromatography (hexane / ethyl acetate, 95:5) to deliver the ketone **24a** (46 mg, 93%) as a colorless oil. R*_f_* = 0.36 (hexane / ethyl acetate, 95:5); ${[\alpha]}_{D}^{23}$ +27.6 (*c* = 0.600, CHCl_3_); ^1^H NMR (500 MHz, CDCl_3_) δ 6.42 (1H, ddd, *J* = 15.2, 11.0, 1.4 Hz), 5.93−5.89 (1H, m), 5.81 (1H, br d, *J* = 11.0 Hz,), 5.52 (1H, dd, *J* = 15.2, 6.1 Hz), 4.22 (1H, ddd, *J* = 6.3, 4.8, 4.2 Hz), 4.22−4.15 (2H, m), 3.98 (1H, app td, *J* = 7.6, 5.4 Hz), 3.08−2.98 (2H, m), 2.97 (1H, dd, *J* = 18.8, 6.3 Hz), 2.76−2.64 (3H, m), 2.65 (1H, dd, *J* = 18.8, 4.8 Hz), 2.49−2.36 (2H, m), 2.07 (1H, dd, *J* = 14.9, 7.3 Hz), 2.04−1.97 (2H, m), 1.96 (1H, dd, *J* = 14.9, 3.0 Hz), 1.90 (1H, dd, *J* = 14.3, 1.0 Hz), 1.90−1.78 (2H, m), 1.84 (3H, d, *J* = 1.1 Hz), 1.77 (3H, d, *J* = 1.3 Hz), 1.75 (3H, s), 1.70 (1H, app ddt, *J* = 12.8, 10.2, 7.6 Hz), 1.48 (1H, app ddt, *J* = 11.9, 10.2, 8.5 Hz), 1.06 (3H, d, *J* = 7.2 Hz), 1.00 (3H, d, *J* = 7.0 Hz), 0.95 (9H, t, *J* = 7.9 Hz), 0.85 (9H, s), 0.59 (6H, q, *J* = 7.9 Hz), 0.02 (3H, s), −0.03 (3H, s); ^13^C NMR (126 MHz, CDCl_3_) δ 212.1, 150.3, 135.2, 130.0, 127.8, 124.8, 82.4, 77.5, 75.6, 74.9, 68.5, 51.9, 48.7, 47.6, 45.6, 43.3, 40.4, 34.0, 27.0, 26.84, 26.2, 26.15, 26.10, 25.1, 24.0, 18.9, 18.4, 18.2, 13.3, 7.1, 5.2, −4.3, −4.6; HRMS (ESI+) *m*/*z*: [M+Na]^+^ calcd for C_39_H_71_IO_4_S_2_Si_2_Na 873.3269, found 873.3230.

**(2*S*,5*S*,6*R*,*E*)-5-(*tert*-Butyldimethylsilyloxy)-8-iodo-2,6,7-trimethyl-1-(2-{(2*R*,5*R*)-5-[(*R*,*E*)-5-methyl-1-(triethylsilyloxy)hexa-2,4-dien-1-yl]tetrahydrofuran-2-yl}methyl-1,3-dithian-2-yl)-oct-7-en-3-one (24b).**

To a solution of β-hydroxy ketone **23b** (20 mg, 27 µmol) in dichloromethane (1.6 mL) at −78 °C, were added 2,6-lutidine (25 µL, 0.22 mmol) and *tert*-butyldimethylsilyl trifluoromethanesulfonate (25 µL, 0.11 mmol) sequentially. The resulting solution was stirred at −78 °C for 5 h, before the addition of water (2 mL) and the biphasic mixture was allowed to warm to rt. The layers were separated, and the aqueous phase was extracted with ether (3 × 2 mL). The combined organic extracts were dried over sodium sulfate, filtered and concentrated. The crude material was purified by silica gel chromatography (hexane / ethyl acetate, 95:5) to deliver ketone **24b** (22 mg, 26 µmol, 95%) as a colorless oil. R*_f_* = 0.34 (pet. ether / ethyl acetate, 95:5); ${[\alpha]}_{D}^{28}$ +23.8 (*c* = 0.650, CHCl_3_); ^1^H NMR (500 MHz, CDCl_3_) δ 6.43 (1H, ddd, *J* = 15.2, 11.0, 1.4 Hz), 5.83 (1H, br s), 5.82 (1H, br d, *J* = 11.0 Hz), 5.52 (1H, dd, *J* = 15.2, 6.1 Hz), 4.22−4.15 (2H, m), 4.07 (1H, app dt, *J* = 9.1, 3.3 Hz), 3.98 (1H, ddd, *J* = 7.8, 7.3, 5.3 Hz), 3.04−2.93 (2H, m), 2.86 (1H, dd, *J* = 18.9, 3.3 Hz), 2.78 (1H, dd, *J* = 18.9, 9.1 Hz), 2.76−2.69 (3H, m), 2.66 (1H, dddd, *J* = 14.3, 5.7, 2.9, 0.9 Hz), 2.49−2.41 (1H, m), 2.06 (1H, dd, *J* = 15.0, 7.4 Hz), 2.07−1.95 (2H, m), 1.97 (1H, dd, *J* = 15.0, 3.2 Hz), 1.88 (1H, dd, *J* = 14.6, 1.0 Hz), 1.90−1.78 (2H, m), 1.82 (3H, d, *J* = 1.1 Hz), 1.77 (3H, s), 1.75 (3H, s), 1.70 (1H, app ddt, *J* = 12.6, 10.1, 7.8 Hz), 1.47 (1H, app ddt, *J* = 11.8, 10.1, 8.4 Hz), 1.07 (3H, d, *J* = 7.1 Hz), 1.02 (3H, d, *J* = 7.1 Hz), 0.95 (9H, t, *J* = 7.9 Hz), 0.88 (9H, s), 0.59 (6H, q, *J* = 7.9 Hz), 0.03 (3H, s), 0.01 (3H, s); ^13^C NMR (126 MHz, CDCl_3_) δ 212.4, 150.1, 135.2, 130.0, 127.8, 124.8, 82.4, 77.6, 75.5, 75.0, 70.2, 51.9, 48.5, 47.4, 45.6, 43.2, 40.4, 34.1, 27.1, 27.0, 26.2, 26.1, 26.0, 25.1, 22.4, 19.2, 18.5, 18.1, 16.2, 7.1, 5.2, −4.4, −4.8; HRMS (ESI+) *m*/*z*: [M+Na]^+^ calcd for C_39_H_71_IO_4_S_2_Si_2_Na 873.3269, found 873.3227.

**(4*S*,7*R*,8*R*,*E*)-7-(*tert*-Butyldimethylsilyloxy)-1-{(2*R*,5*R*)-5-[(*R*,*E*)-1-hydroxy-5-methylhexa-2,4-dien-1-yl]tetrahydrofuran-2-yl}-10-iodo-4,8,9-trimethyldec-9-ene-2,5-dione (27).**

To a solution of dithiane **24a** (6 mg, 7 µmol) in a mixture (10:4:1) of acetonitrile, THF and water (400 µL) at 0 °C, were added calcium carbonate (7 mg, 0.07 mmol) and mercury(II) perchlorate tetrahydrate (6 mg, 0.01 mmol) sequentially. The resulting mixture was stirred at 0 °C for 15 min during which time a white precipitate formed. Saturated aqueous sodium bicarbonate solution (600 µL) and ether (600 µL) were added and the phases were separated. The aqueous phase was extracted with ether (3 × 1 mL) and the combined organic extracts were dried over sodium sulfate, filtered and concentrated. A mixture (9:1 ratio) of the 1,4-diketone **27** and TES protected compound was observed by ^1^H NMR analysis of the crude mixture. The crude product was purified by silica gel chromatography (hexane/ethyl acetate, 70:30) to afford 1,4-diketone **27** (3 mg, 5 µmol, 77%) as a colorless oil. R*_f_* = 0.36 (hexane / ethyl acetate, 70:30); ${[\alpha]}_{D}^{24}$ +15 (*c* = 0.38, CHCl_3_); ν_max._ 3426, 2957, 2926, 2855, 1709, 1663, 1634, 963, 937, 874, 835, 800, 775, 721, 681 cm^−1^; ^1^H NMR (500 MHz, CDCl_3_) δ 6.53 (1H, ddd, *J* = 15.1, 10.9, 1.2 Hz), 5.95 (1H, br s), 5.82 (1H, br d, *J* = 10.9 Hz), 5.45 (1H, dd, *J* = 15.1, 6.9 Hz), 4.28 (1H, app ddt, *J* = 8.5, 7.1, 5.8 Hz), 4.21 (1H, ddd, *J* = 5.9, 5.4, 4.9 Hz), 3.95 (1H, ddd, *J* = 7.2, 6.9, 1.2 Hz), 3.87 (1H, app q, *J* = 7.2 Hz), 3.04 (1H, dd, *J* = 17.8, 9.0 Hz), 3.00−2.92 (1H, m), 2.75 (1H, dd, *J* = 17.8, 5.9 Hz), 2.74 (1H, dd, *J* = 15.1, 7.1 Hz), 2.72−2.69 (1H, m), 2.63 (1H, dd, *J* = 17.8, 5.4 Hz), 2.50 (1H, dd, *J* = 15.1, 5.8 Hz), 2.46 (1H, qd, *J* = 7.0, 4.9 Hz), 2.39 (1H, dd, *J* = 17.8, 4.2 Hz), 2.13 (1H, dddd, *J* = 12.0, 8.3, 5.8, 3.2 Hz), 1.95 (1H, dddd, *J* = 12.5, 8.5, 7.2, 3.2 Hz), 1.83 (3H, d, *J* = 0.6 Hz), 1.78 (3H, s), 1.76 (3H, s), 1.66 (1H, dddd, *J* = 12.5, 9.8, 8.3, 7.2 Hz), 1.53 (1H, app ddt, *J* = 12.0, 9.8, 8.5 Hz), 1.07 (3H, d, *J* = 7.0 Hz), 1.06 (3H, d, *J* = 7.1 Hz), 0.85 (9H, s), 0.03 (3H, s), −0.04 (3H, s); ^13^C NMR (126 MHz, CDCl_3_) δ 211.1, 207.7, 150.0, 136.7, 129.5, 128.1, 124.6, 82.4, 77.8, 75.7, 75.4, 69.3, 48.6, 48.4, 47.6, 46.5, 41.6, 32.5, 28.1, 26.2, 26.1, 23.8, 18.5, 18.2, 16.4, 14.0, −4.3, −4.5; HRMS (ESI+) *m*/*z*: [M+Na]^+^ calcd for C_30_H_51_IO_5_SiNa 669.2443, found 669.2412.

**(*R*,*E*)-1-{(2*R*,5*R*)-5-[(4*S*,7*R*,8*R*,*E*)-7-(*tert*-Butyldimethylsilyloxy)-10-iodo-4,8,9-trimethyl-2,5-dioxodec-9-en-1-yl]tetrahydrofuran-2-yl}-5-methylhexa-2,4-dien1-yl 2-((2*S*,3*R*,5*R*)-3-methyl-5-{(5*S*,6*S*)-2,2,3,3,8,8,9,9-octamethyl-6-[1-(tributylstannyl)vinyl]-4,7-dioxa-3,8-disiladecan-5-yl}-tetrahydrofuran-2-yl)acetate (28).**

To a solution of acid **17** (dried by azeotropic distillation with benzene twice, 9.4 mg, 13 µmol) in toluene (220 µL) at rt, were added triethylamine (15 µL, 0.11 mmol) in toluene (100 µL) and 2,4,6-trichlorobenzoyl chloride (4 µL, 0.03 mmol) in toluene (100 µL) sequentially. The resulting mixture was stirred at rt for 1 h to give the mixed anhydride.

To a separate vial containing alcohol **27** (dried by azeotropic distillation with benzene twice, 7.5 mg, 12 µmol) was added 4-dimethylaminopyridine (5 mg, 0.04 mmol) in toluene (100 µL). The resulting solution was added to the vial containing the mixed anhydride and the vial was rinsed with toluene (3 ×100 µL). The cloudy mixture was stirred at rt for 3 h and diluted with toluene (1.6 mL). The reaction was quenched by the addition of saturated aqueous sodium bicarbonate solution (1 mL) and the phases were separated. The aqueous phase was diluted with water (3 mL) and extracted with toluene (3 × 3 mL). The combined organic extracts were washed with brine (10 mL), dried over sodium sulfate, filtered and concentrated. The crude product was purified by silica gel chromatography (hexane / ethyl acetate, 90:10) to afford the ester **28** (11 mg, 69%) as a colorless oil. R*_f_* = 0.33 (hexane / ethyl acetate, 90:10); ${[\alpha]}_{D}^{22}$ −2.9 (*c* = 0.55, CHCl_3_); ^1^H NMR (500 MHz, CDCl_3_) δ 6.49 (1H, ddd, *J* = 15.1, 11.1, 0.8 Hz), 5.95−5.94 (1H, m), 5.93 (1H, dd, *J* = 2.7, 1.9 Hz, ^3^*J*_SnH_ = 132.0 Hz), 5.78 (1H, br d, *J* = 11.1 Hz), 5.42 (1H, dd, *J* = 15.1, 7.8 Hz), 5.28 (1H, ddd, *J* = 7.8, 6.2, 0.8 Hz), 5.23 (1H, dd, *J* = 2.7, 1.8 Hz, ^3^*J*_SnH_ = 63.7 Hz), 4.35−4.27 (2H, m), 4.24−4.16 (2H, m), 4.10−4.03 (2H, m), 3.51 (1H, dd, *J* = 7.4, 2.5 Hz), 2.97−2.89 (2H, m), 2.74 (1H, dd, *J* = 17.8, 6.0 Hz), 2.70 (1H, dd, *J* = 15.5, 6.2 Hz), 2.62 (1H, dd, *J* = 17.8, 5.4 Hz), 2.59 (1H, dd, *J* = 15.8, 6.6 Hz), 2.51 (1H, dd, *J* = 15.5, 6.8 Hz), 2.48−2.38 (3H, m), 2.31−2.22 (1H, m), 2.12 (1H, dddd, *J* = 12.0, 8.0, 5.7, 3.6 Hz), 1.94 (1H, dddd, *J* = 12.4, 8.3, 7.0, 3.6 Hz), 1.83 (3H, d, *J* = 1.1 Hz), 1.77 (3H, s), 1.74 (3H, s), 1.78−1.64 (2H, m), 1.54−1.41 (8H, m), 1.32 (6H, h, *J* = 7.3 Hz), 1.06 (3H, d, *J* = 6.7 Hz), 1.06 (3H, d, *J* = 7.0 Hz), 0.91 (9H, s), 0.88 (9H, s), 0.85 (9H, s), 0.96−0.83 (18H, m), 0.08 (3H, s), 0.07 (3H, s), 0.06 (3H, s), 0.03 (3H, s), −0.01 (3H, s), −0.04 (3H, s); ^13^C NMR (126 MHz, CDCl_3_) δ 211.1, 207.4, 170.7, 154.2, 150.0, 137.6, 131.4, 125.8, 124.5, 124.4, 82.7, 79.7, 79.3, 78.8, 77.7, 76.54, 76.45, 75.4, 69.3, 48.9, 48.4, 47.5, 45.8, 41.6, 37.6, 36.7, 36.0, 32.1, 29.3, 28.0, 27.6, 26.6, 26.3, 26.2, 26.1, 23.9, 18.8, 18.7, 18.6, 18.2, 16.4, 14.2, 13.89, 13.86, 10.3, −3.6, −3.9, −4.2, −4.3, −4.3, −4.5; HRMS (ESI+) *m*/*z*: [M+Na]^+^ calcd for C_65_H_121_IO_9_Si_3_SnNa 1399.6277, found 1399.6253.

**References**

1. Palmer, C.; Morra, N. A.; Stevens, A. C.; Bajtos, B.; Machin, B. P.; Pagenkopf, B. L. Increased Yields and Simplified Purification with a Second-Generation Cobalt Catalyst for the Oxidative Formation of *trans*-THF Rings. *Org. Lett.* **2009**, *11*, 5614–5617.

2. Hoffmann, R. W.; Weidmann, U. *Chem. Ber.* **1985**, *118*, 3966–3979.

3. Kim, C. H.; An, H. J.; Shin, W. K.; Yu, W.; Woo, S. K.; Jung, S. K.; Lee, E. Total Synthesis of (−)-Amphidinolide E. *Angew. Chem. Int. Ed.* **2006**, *45*, 8019–8021.

4. Romiti, F.; Decultot, L.; Clark, J. S. Convergent Synthesis of the C1–C29 Framework of Amphidinolide F. *J. Org. Chem.* **2022**, *87*, 8126–8141.

5. Ferrié, L.; Fenneteau, J.; Figadère, B. Total Synthesis of the Marine Macrolide Amphidinolide F. *Org. Lett.* **2018**, *20*, 3192–3196.

6. Hoye, T. R.; Jeffrey, C. S.; Shao, F. Mosher Ester Analysis for the Determination of Absolute Configuration of Stereogenic (Chiral) Carbinol Carbons. *Nat. Protoc.* **2007**, *2*, 2451–2458.

**^1^H and ^13^C NMR Spectra for New Compounds**

Page

^1^H NMR Spectrum of **2** (400 MHz, CDCl_3_) SI-27

^13^C{^1^H} NMR Spectrum of **2** (101 MHz, CDCl_3_) SI-28

^1^H NMR Spectrum of **3** (400 MHz, CDCl_3_) SI-29

^13^C{^1^H} NMR Spectrum of **3** (101 MHz, CDCl_3_) SI-30

^1^H NMR Spectrum of **5a,b** (500 MHz, CDCl_3_) SI-31

^13^C{^1^H} NMR Spectrum of **5a,b** (126 MHz, CDCl_3_) SI-32

^1^H NMR Spectrum of **4** (500 MHz, CDCl_3_) SI-33

^1^H NMR Spectrum of **5a** (500 MHz, CDCl_3_) SI-34

^13^C{^1^H} NMR Spectrum of **5a** (126 MHz, CDCl_3_) SI-35

^1^H NMR Spectrum of **S1** (500 MHz, CDCl_3_) SI-36

^13^C{^1^H} NMR Spectrum of **S1** (126 MHz, CDCl_3_) SI-37

^1^H NMR Spectrum of **6** (400 MHz, CDCl_3_) SI-38

^13^C{^1^H} NMR Spectrum of **6** (126 MHz, CDCl_3_) SI-39

^1^H NMR Spectrum of **S2** (500 MHz, CDCl_3_) SI-40

^13^C{^1^H} NMR Spectrum of **S2** (126 MHz, CDCl_3_) SI-41

^1^H NMR Spectrum of **8** (500 MHz, CDCl_3_) SI-42

^13^C{^1^H} NMR Spectrum of **8** (126 MHz, CDCl_3_) SI-43

^1^H NMR Spectrum of **9** (500 MHz, CDCl_3_) SI-44

^13^C{^1^H} NMR Spectrum of **9** (126 MHz, CDCl_3_) SI-45

^1^H NMR Spectrum of **11** (400 MHz, CDCl_3_) SI-46

^13^C{^1^H} NMR Spectrum of **11** (126 MHz, CDCl_3_) SI-47

^1^H NMR Spectrum of **12** (500 MHz, CDCl_3_) SI-48

^13^C{^1^H} NMR Spectrum of **12** (126 MHz, CDCl_3_) SI-49

^1^H NMR Spectrum of **15** (500 MHz, CDCl_3_) SI-50

^13^C{^1^H} NMR Spectrum of **15** (126 MHz, CDCl_3_) SI-51

^1^H NMR Spectrum of **16** (500 MHz, CDCl_3_) SI-52

^1^H NMR Spectrum of **17** (500 MHz, CDCl_3_) SI-53

^13^C{^1^H} NMR Spectrum of **17** (126 MHz, CDCl_3_) SI-54

^1^H NMR Spectrum of **S3** (500 MHz, CDCl_3_) SI-55

^1^H NMR Spectrum of **18** (500 MHz, CDCl_3_) SI-56

^13^C{^1^H} NMR Spectrum of **18** (126 MHz, CDCl_3_) SI-57

^1^H NMR Spectrum of **19** (500 MHz, CDCl_3_) SI-58

^13^C{^1^H} NMR Spectrum of **19** (126 MHz, CDCl_3_) SI-59

^1^H NMR Spectrum of **20** (500 MHz, CDCl_3_) SI-60

^13^C{^1^H} NMR Spectrum of **20** (126 MHz, CDCl_3_) SI-61

^1^H NMR Spectrum of **21** (500 MHz, CDCl_3_) SI-62

^13^C{^1^H} NMR Spectrum of **21** (126 MHz, CDCl_3_) SI-63

^1^H NMR Spectrum of **22** (500 MHz, CDCl_3_) SI-64

^13^C{^1^H} NMR Spectrum of **22** (126 MHz, CDCl_3_) SI-65

^1^H NMR Spectrum of **23a** (500 MHz, CDCl_3_) SI-66

^13^C{^1^H} NMR Spectrum of **23a** (126 MHz, CDCl_3_) SI-67

^1^H NMR Spectrum of **23b** (500 MHz, CDCl_3_) SI-68

^13^C{^1^H} NMR Spectrum of **23b** (126 MHz, CDCl_3_) SI-69

^1^H NMR Spectrum of **S4** (500 MHz, CDCl_3_) SI-70

^1^H NMR Spectrum of **S5** (500 MHz, CDCl_3_) SI-71

^1^H NMR Spectrum of **24a** (500 MHz, CDCl_3_) SI-72

^13^C{^1^H} NMR Spectrum of **24a** (126 MHz, CDCl_3_) SI-73

^1^H NMR Spectrum of **24b** (500 MHz, CDCl_3_) SI-74

^13^C{^1^H} NMR Spectrum of **24b** (126 MHz, CDCl_3_) SI-75

^1^H NMR Spectrum of **27** (500 MHz, CDCl_3_) SI-76

^13^C{^1^H} NMR Spectrum of **27** (126 MHz, CDCl_3_) SI-77

^1^H NMR Spectrum of **28** (500 MHz, CDCl_3_) SI-78

^13^C{^1^H} NMR Spectrum of **28** (126 MHz, CDCl_3_) SI-79


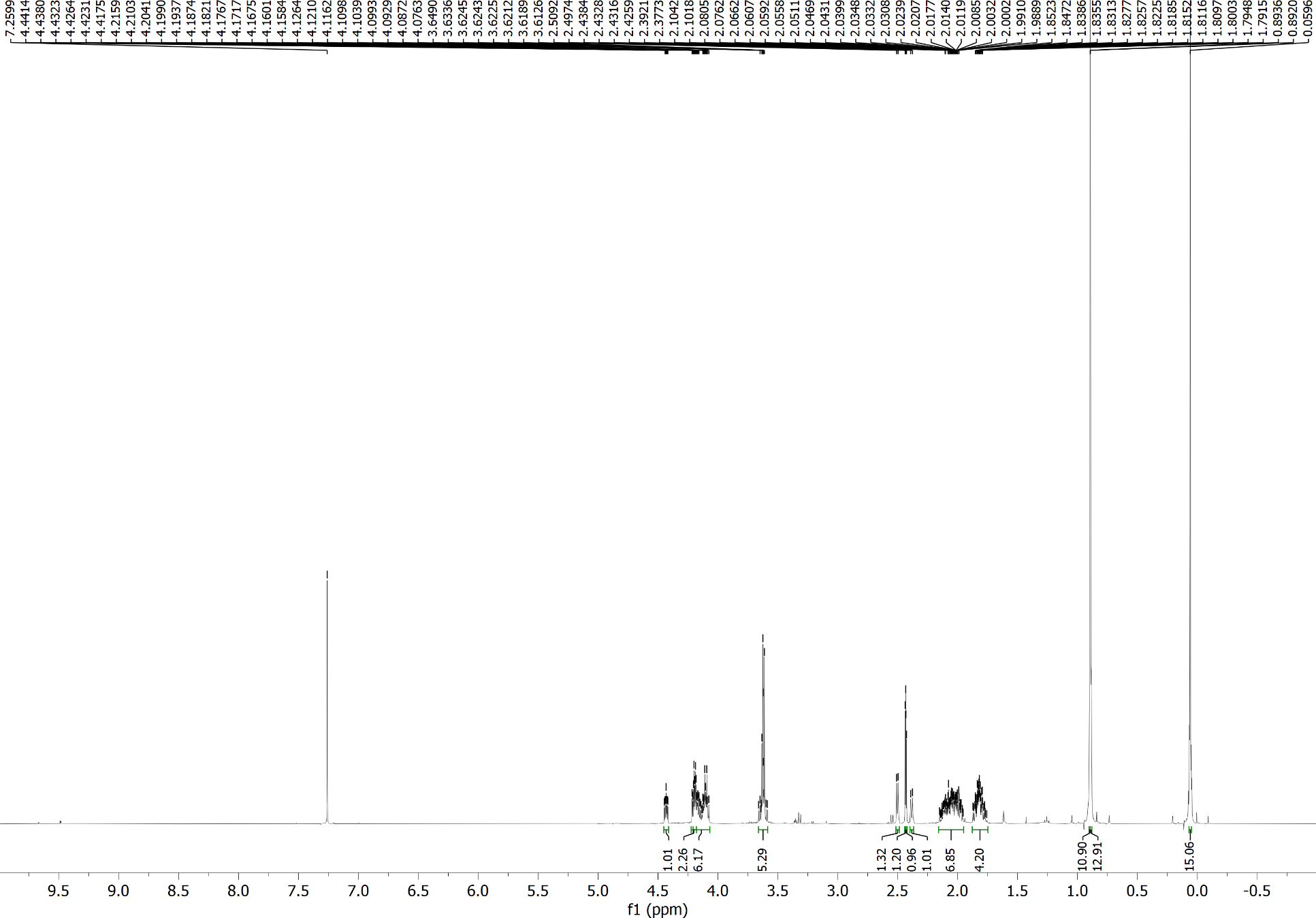


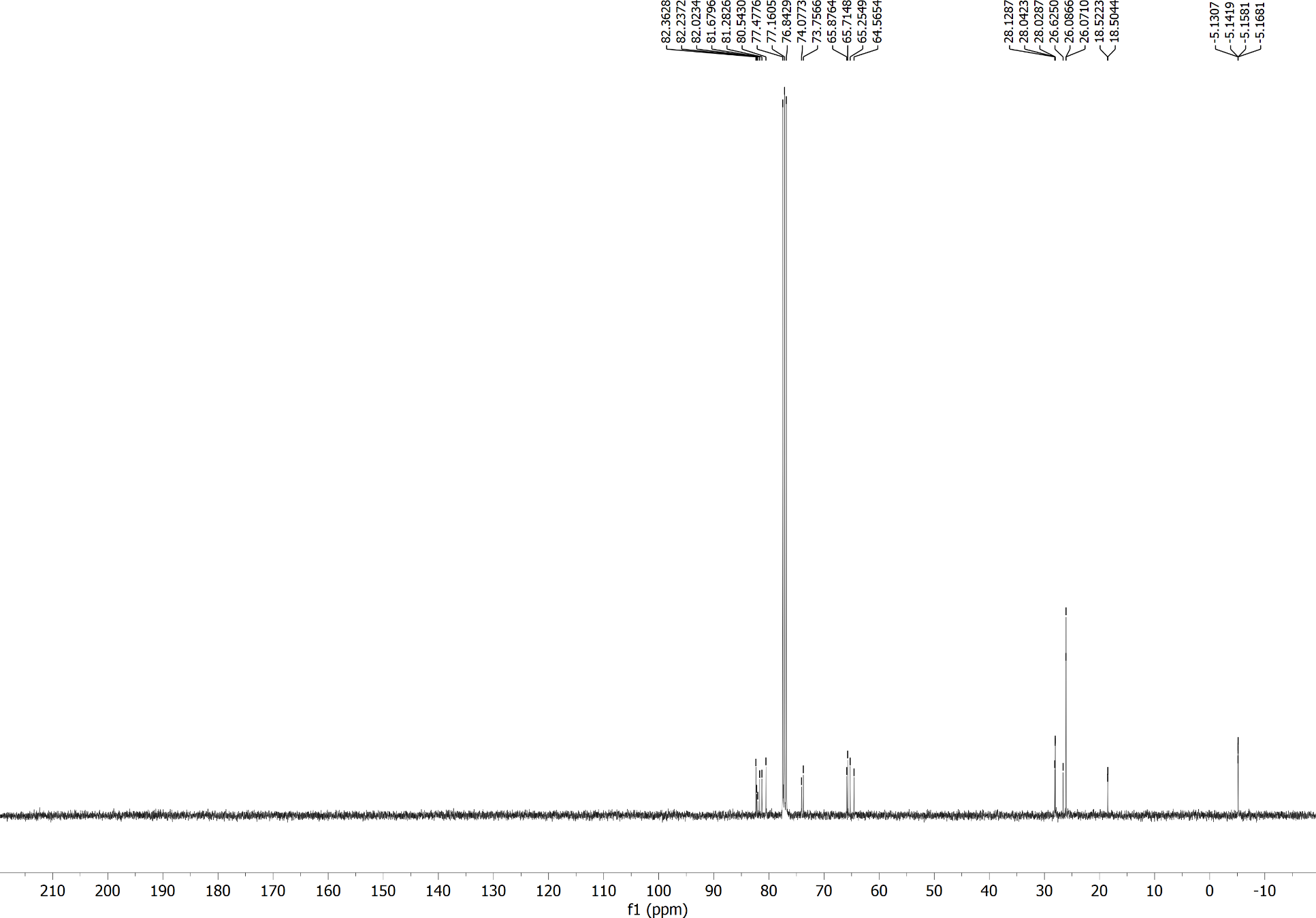


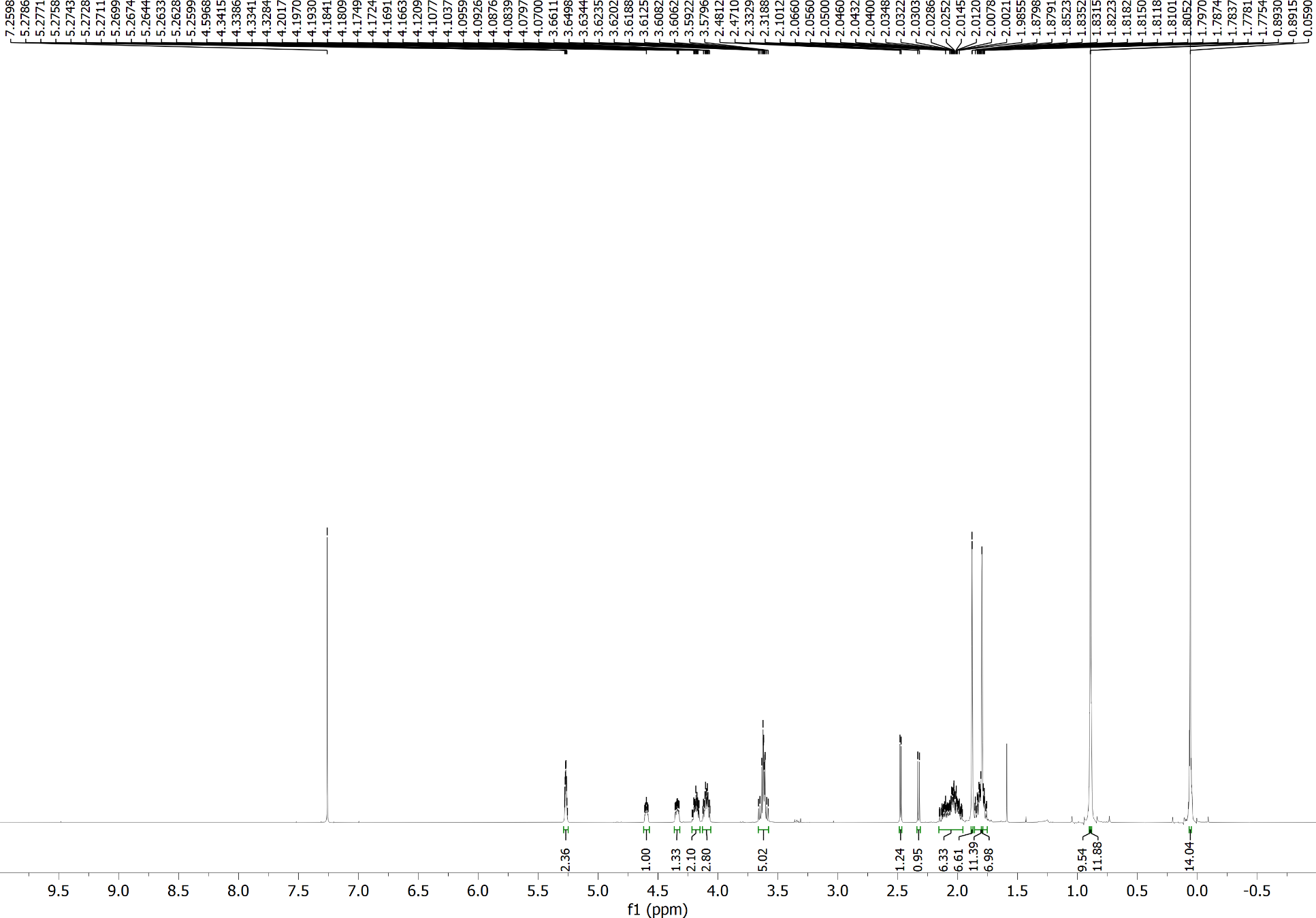


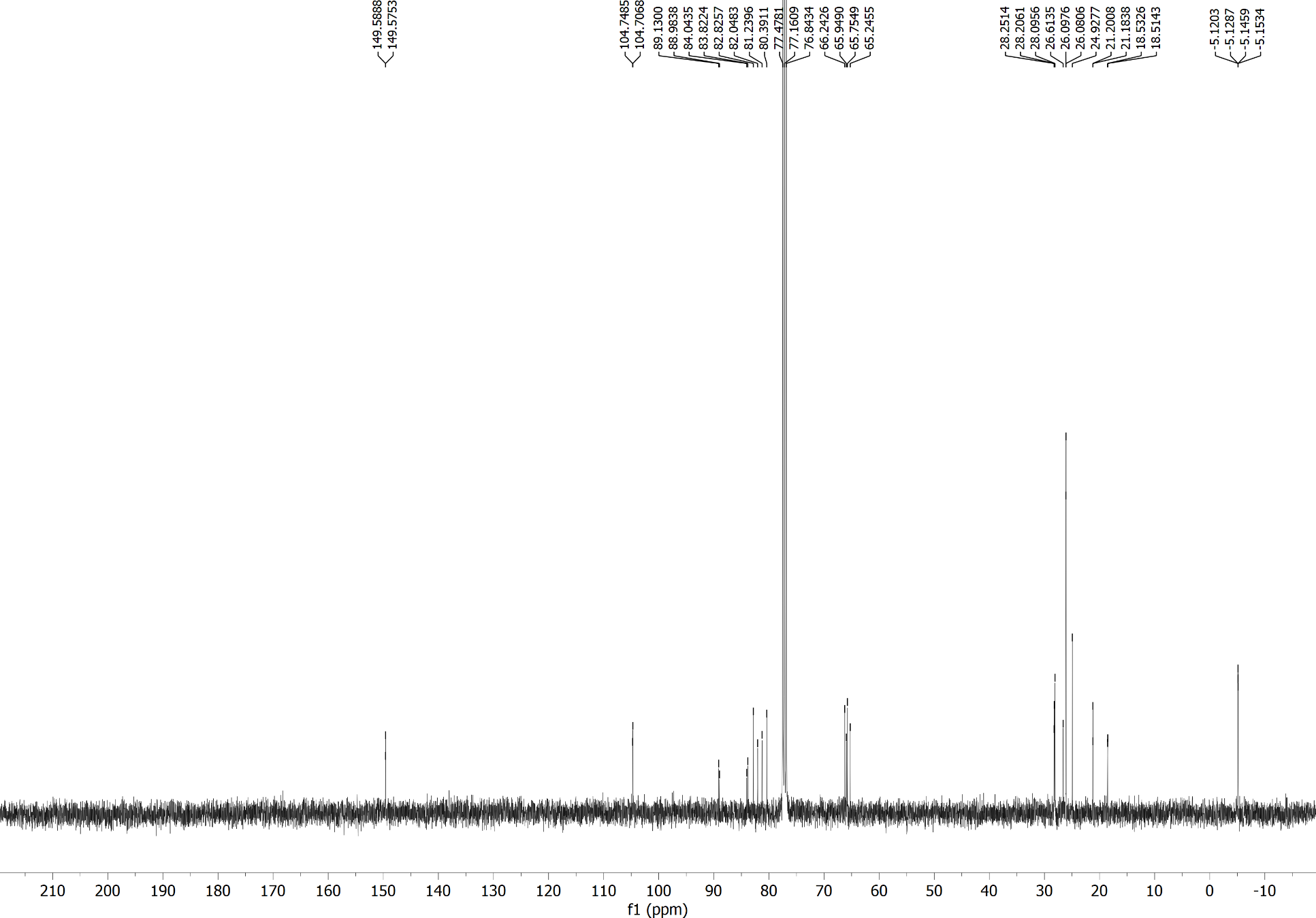


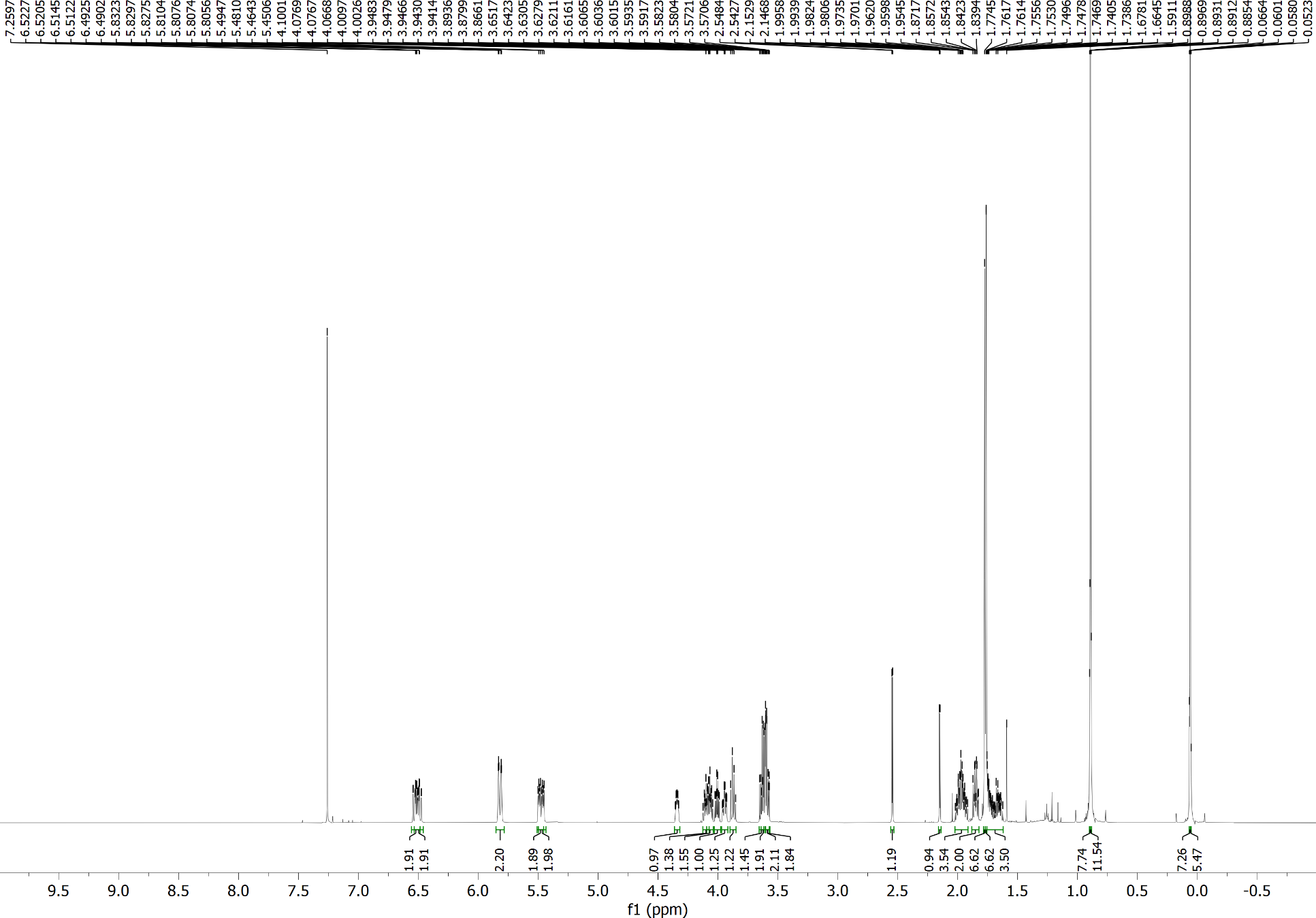


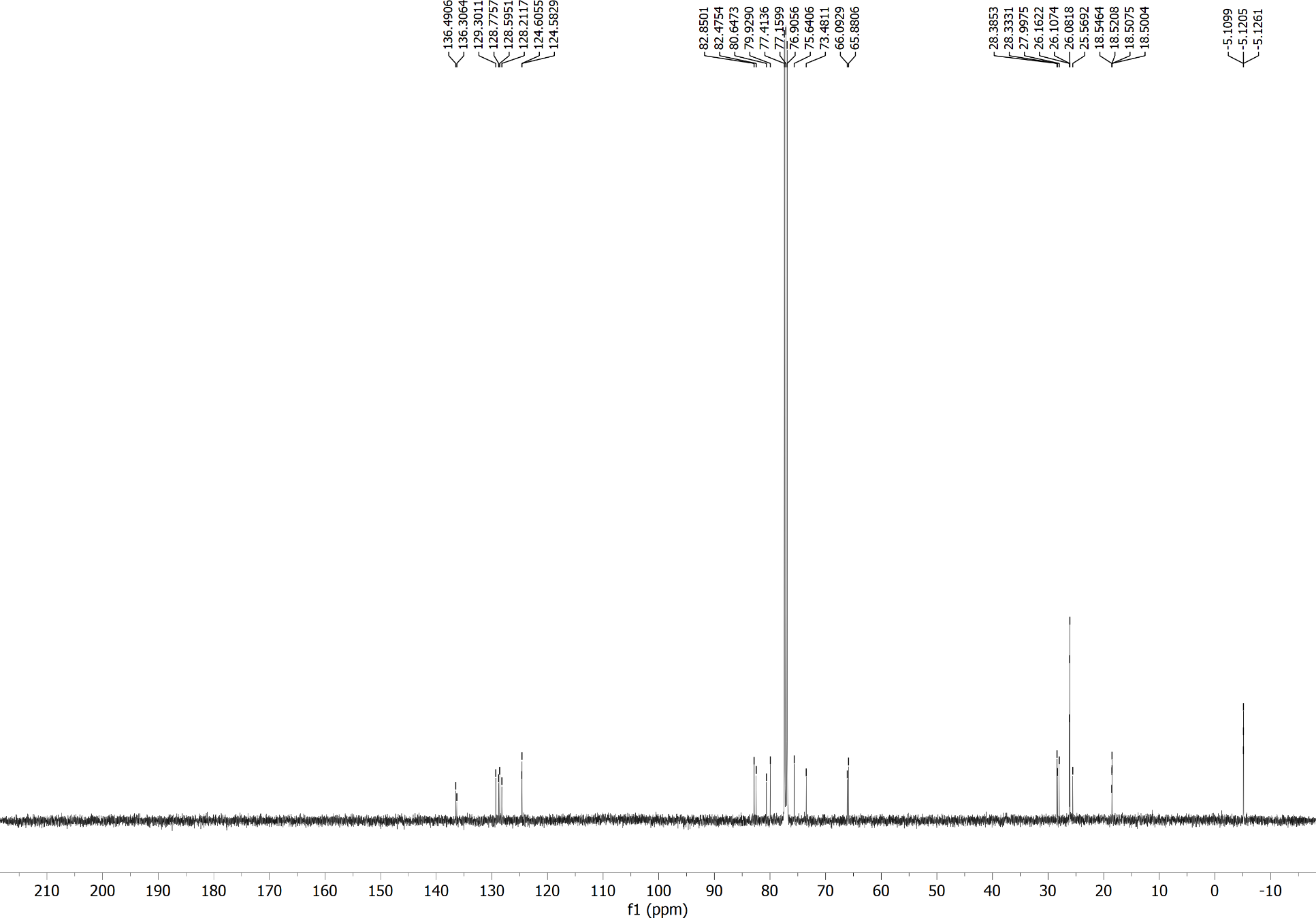


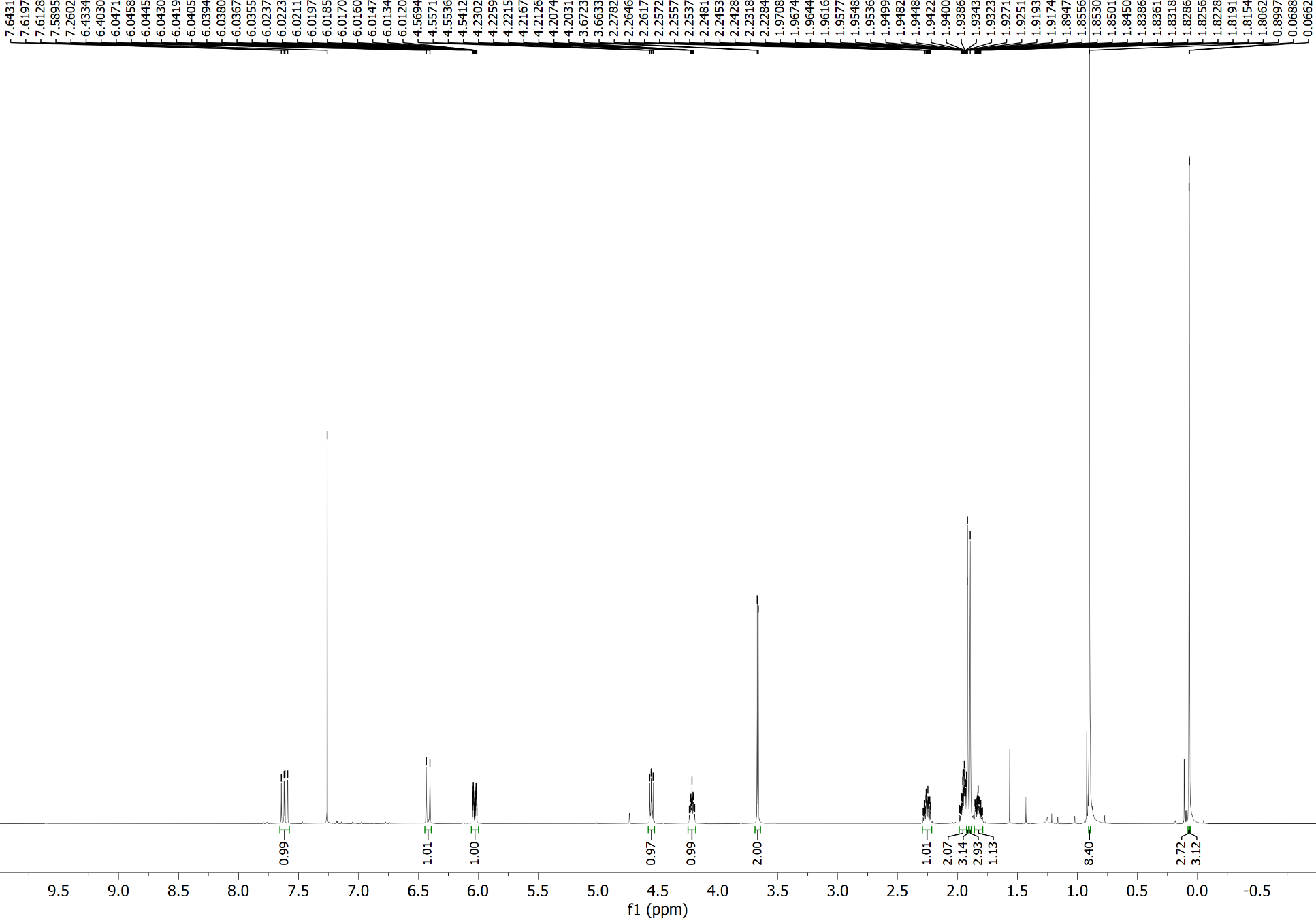


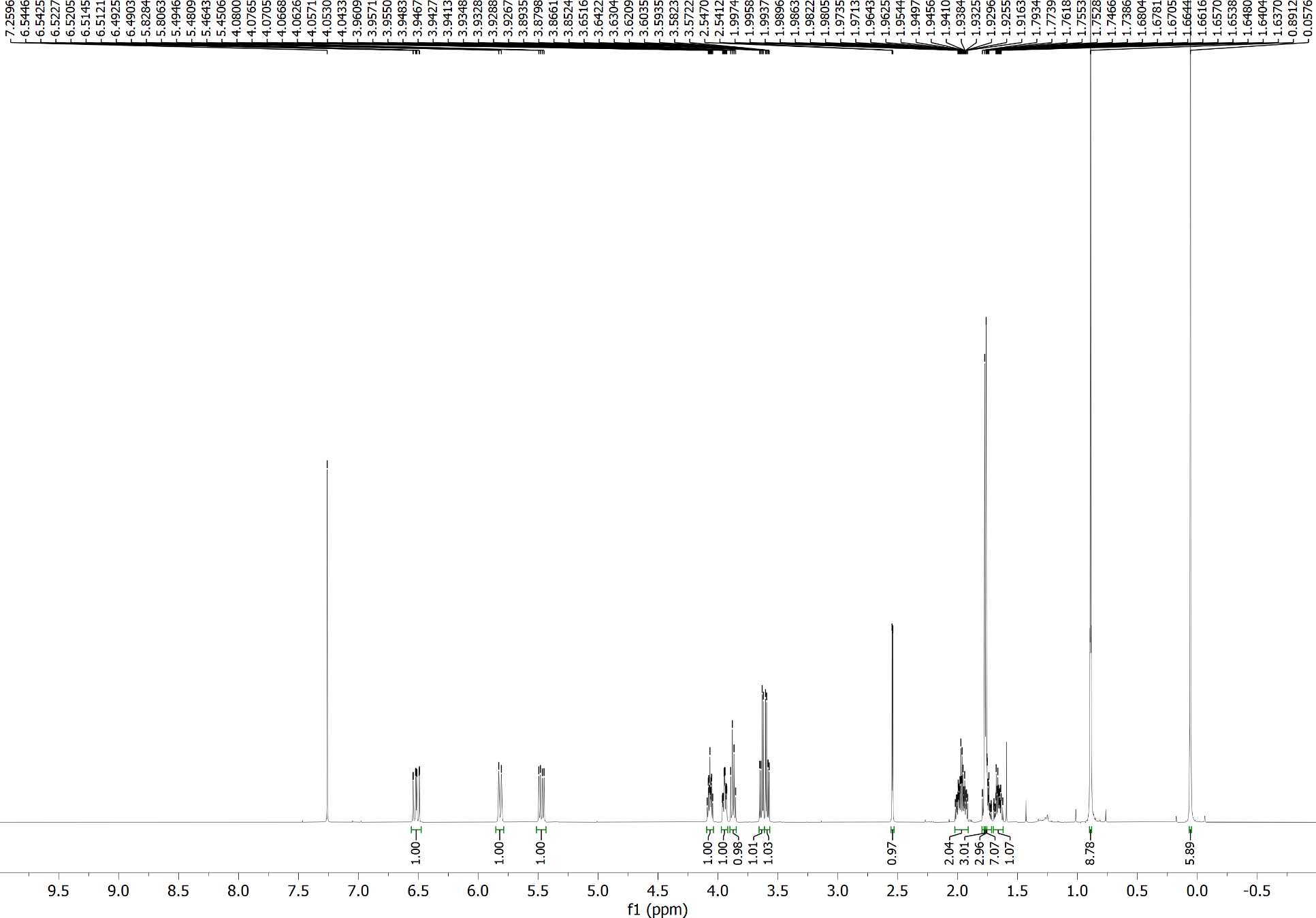


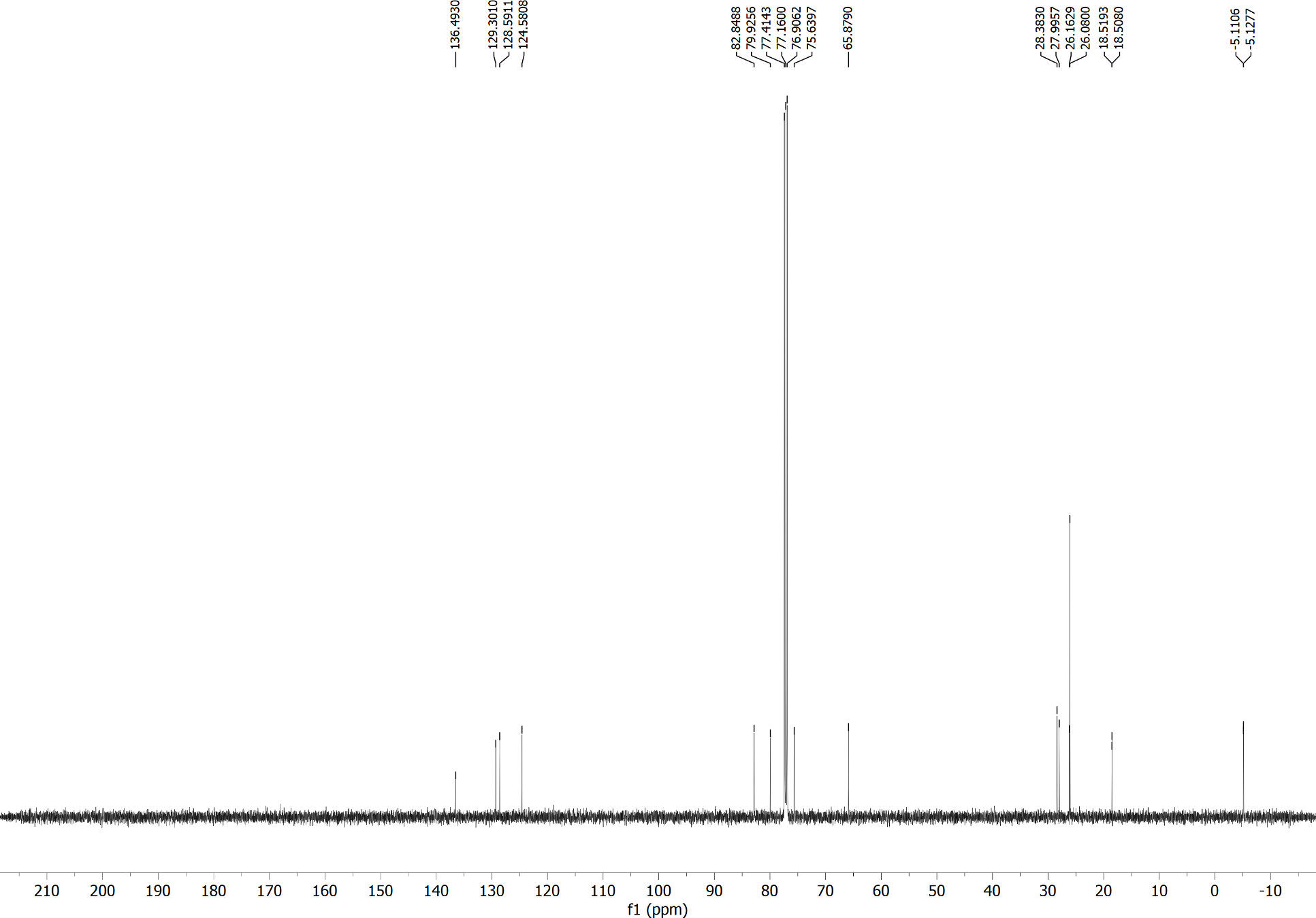


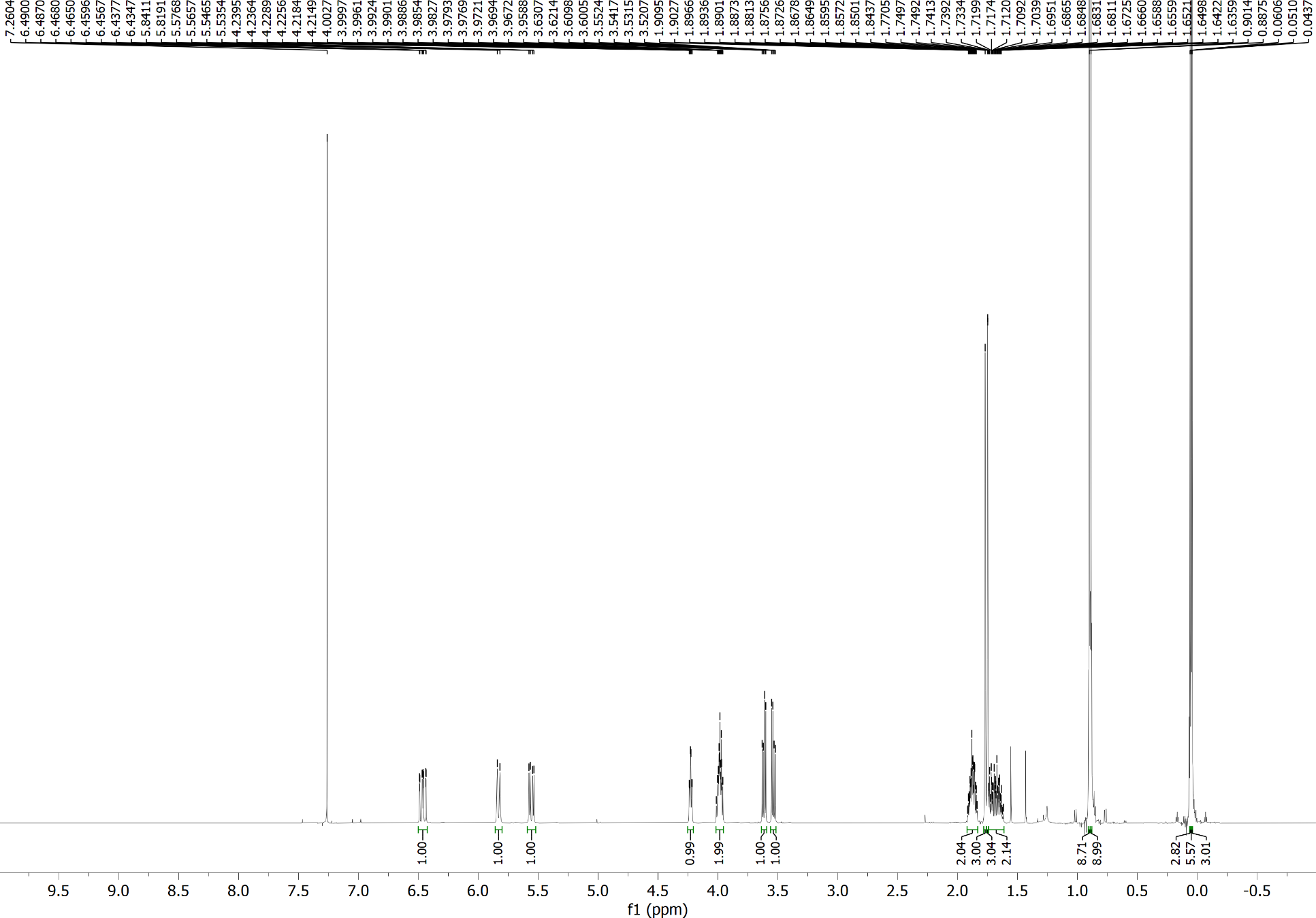


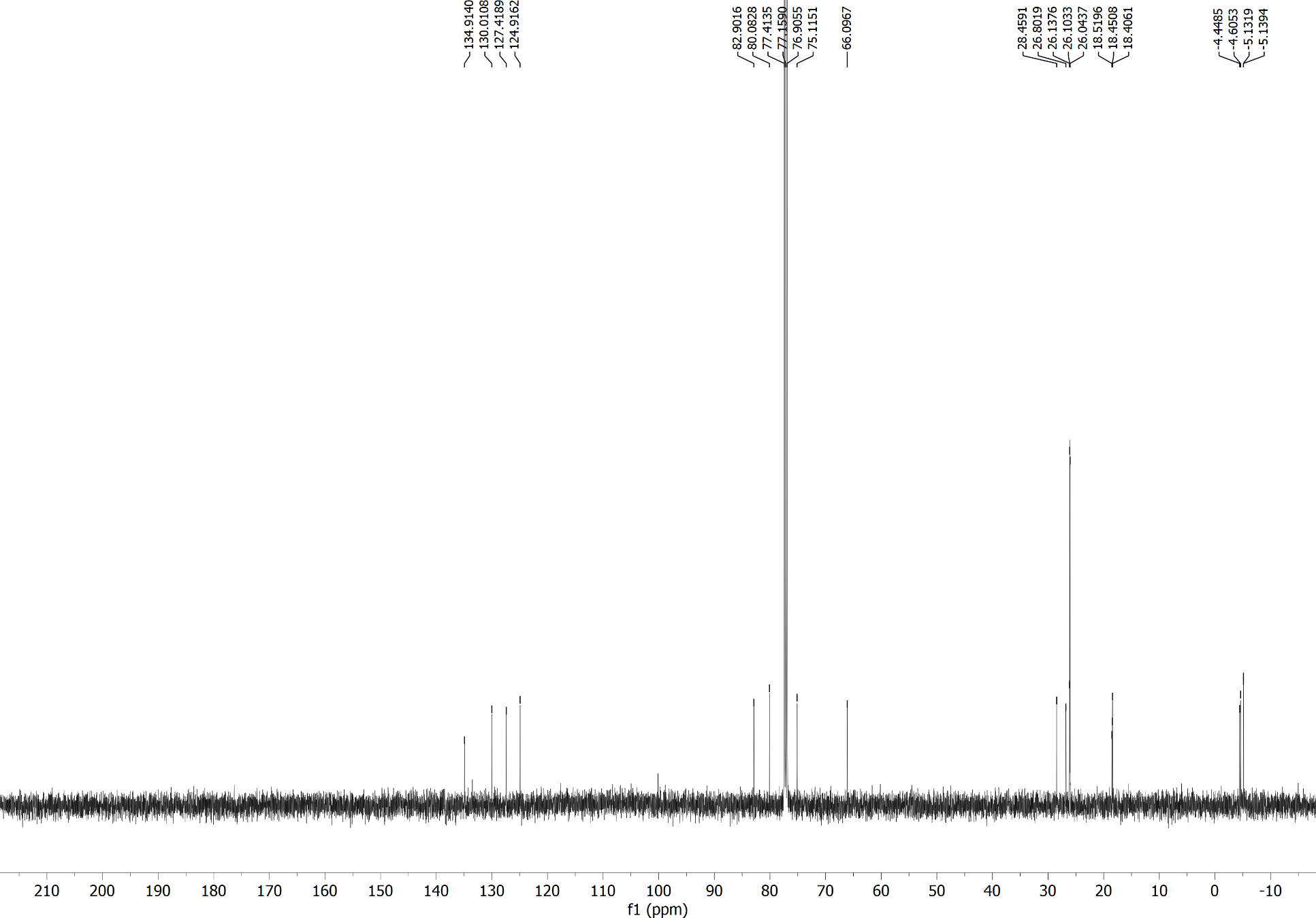


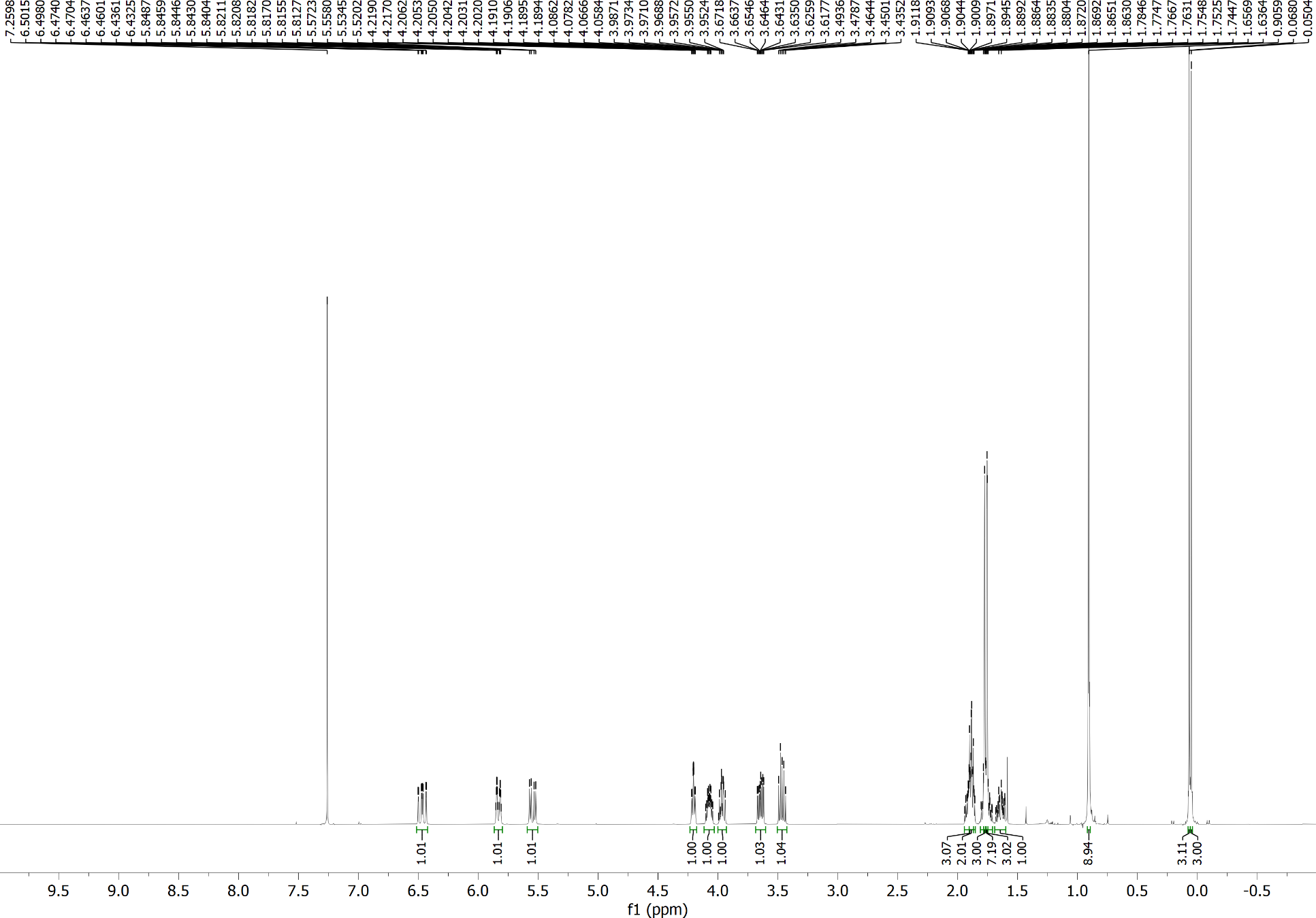


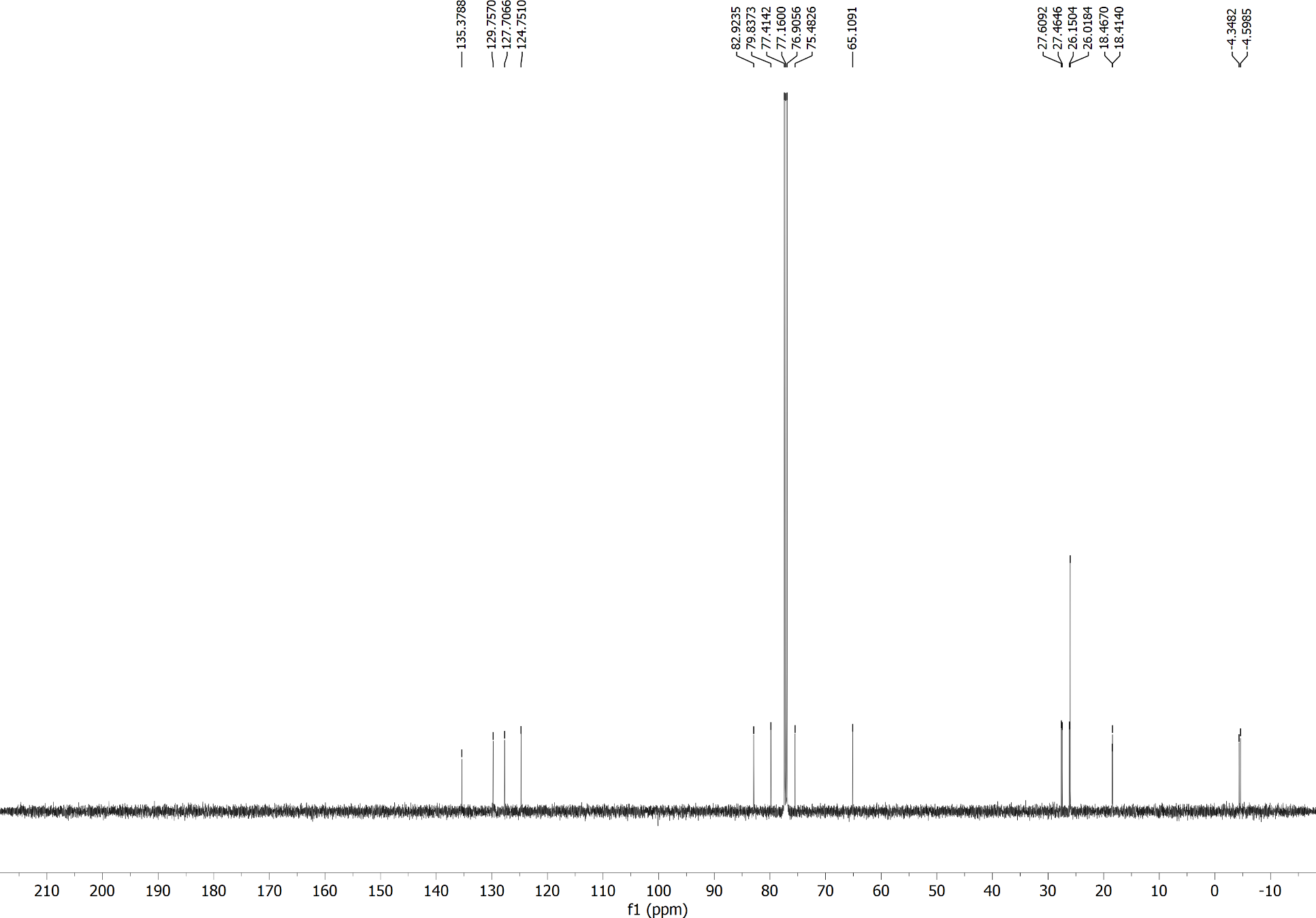


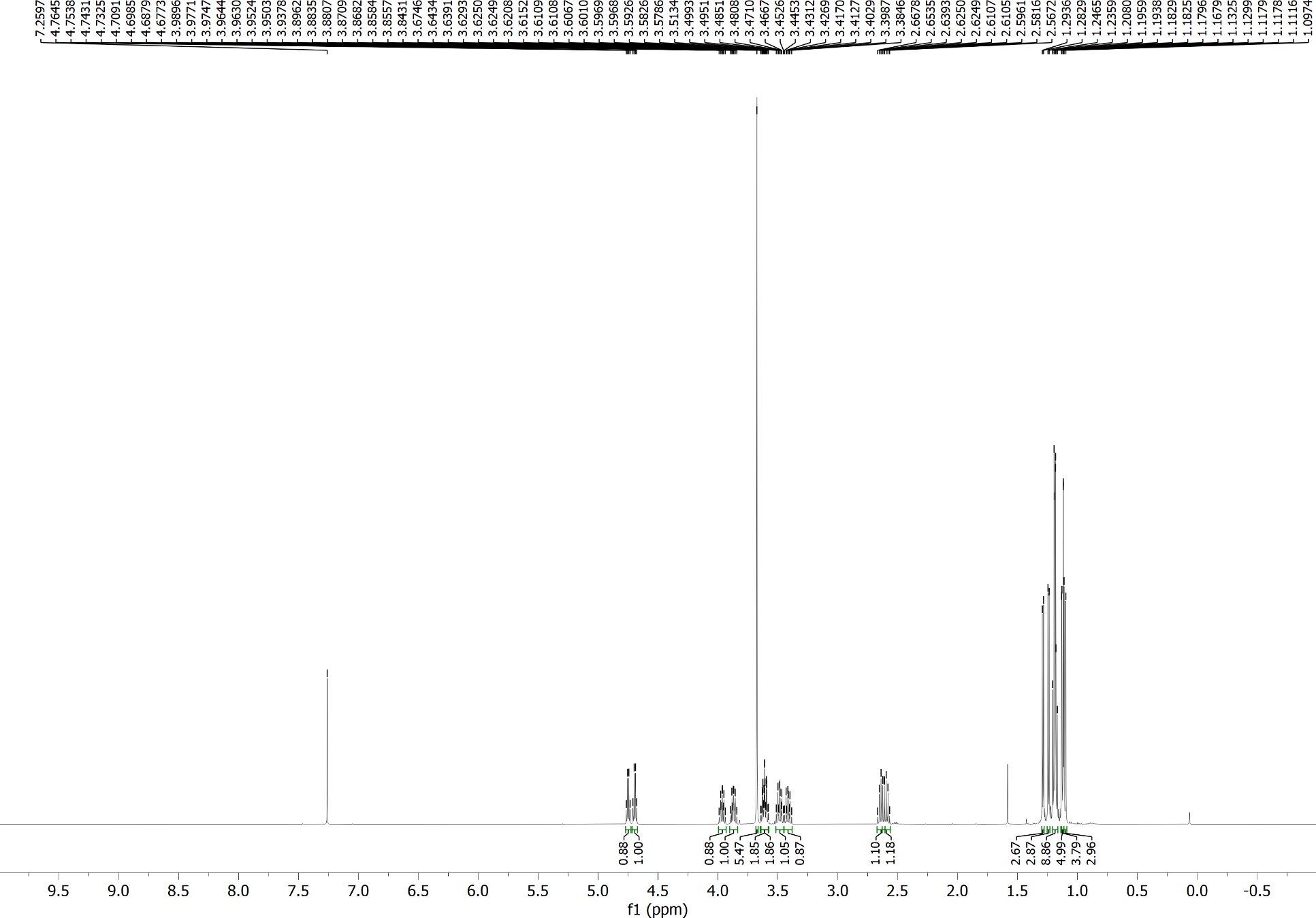


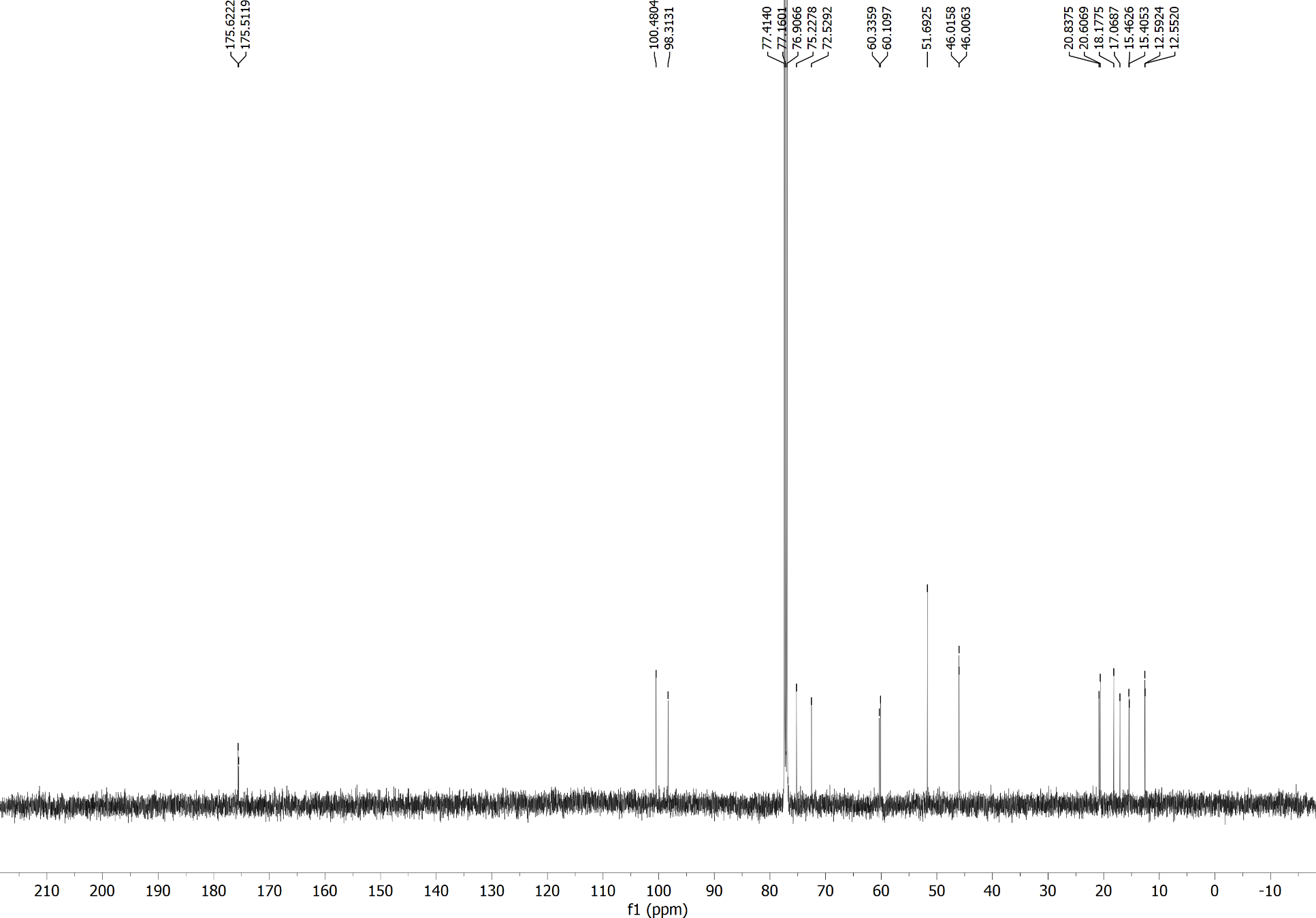


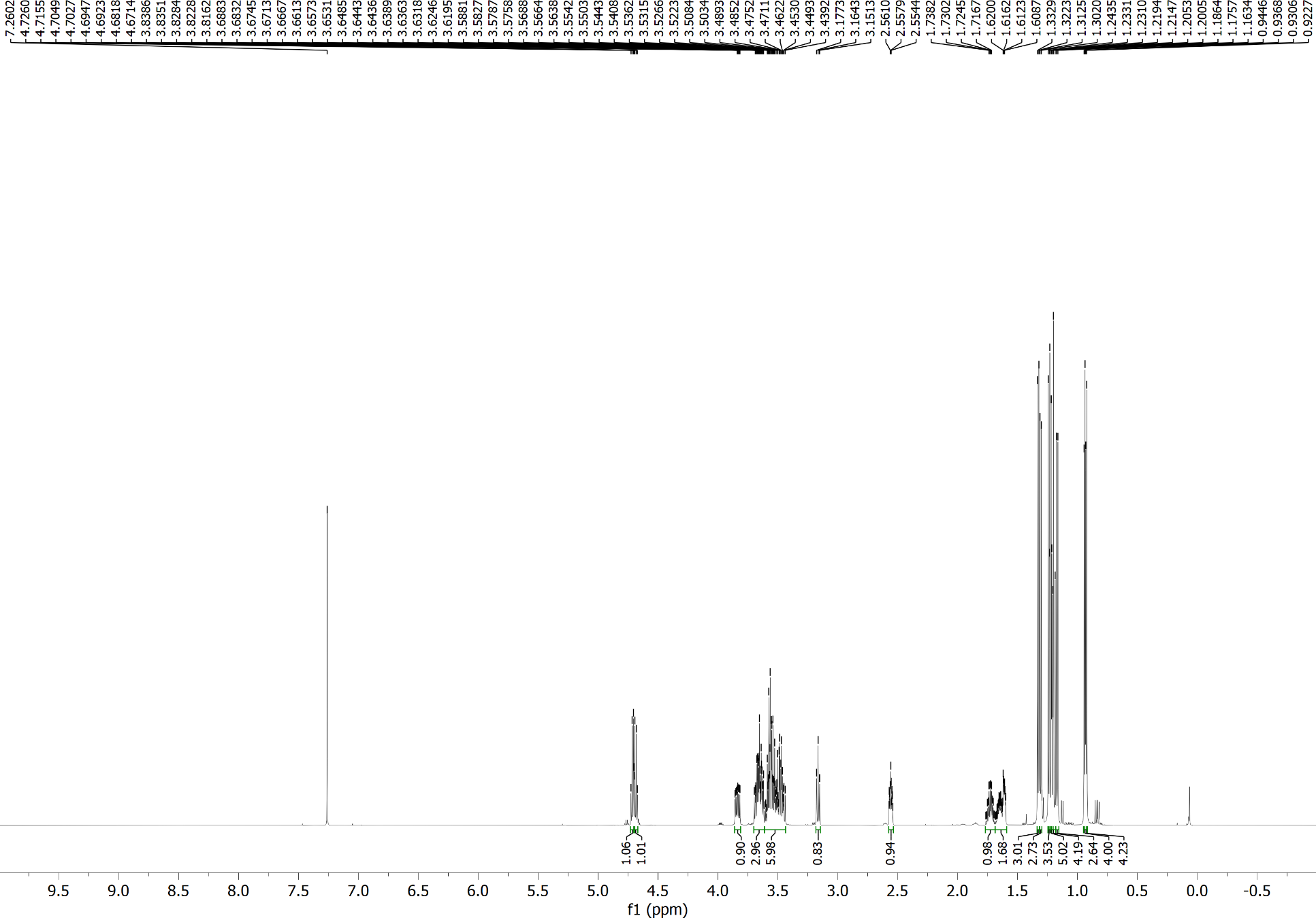


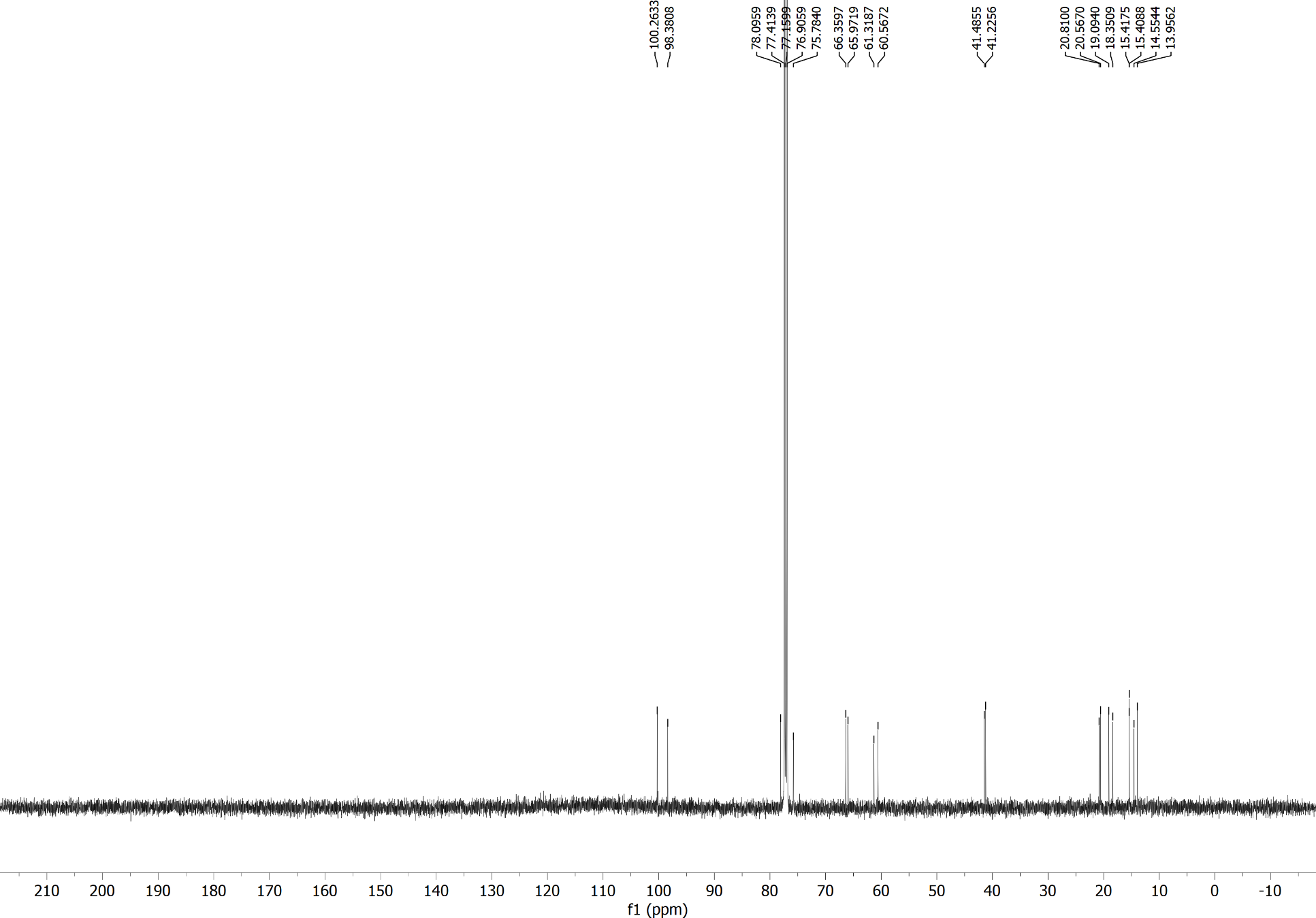


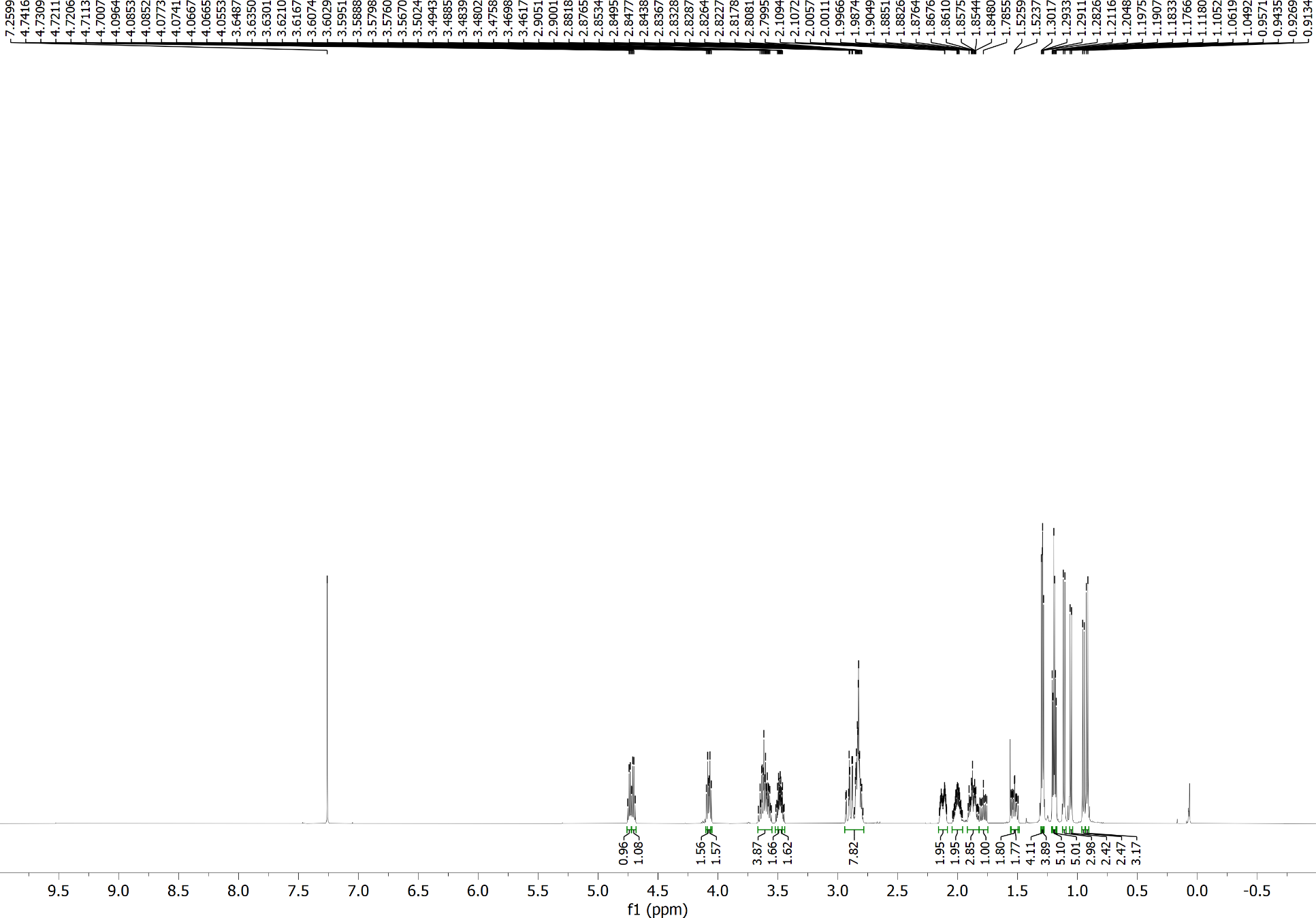


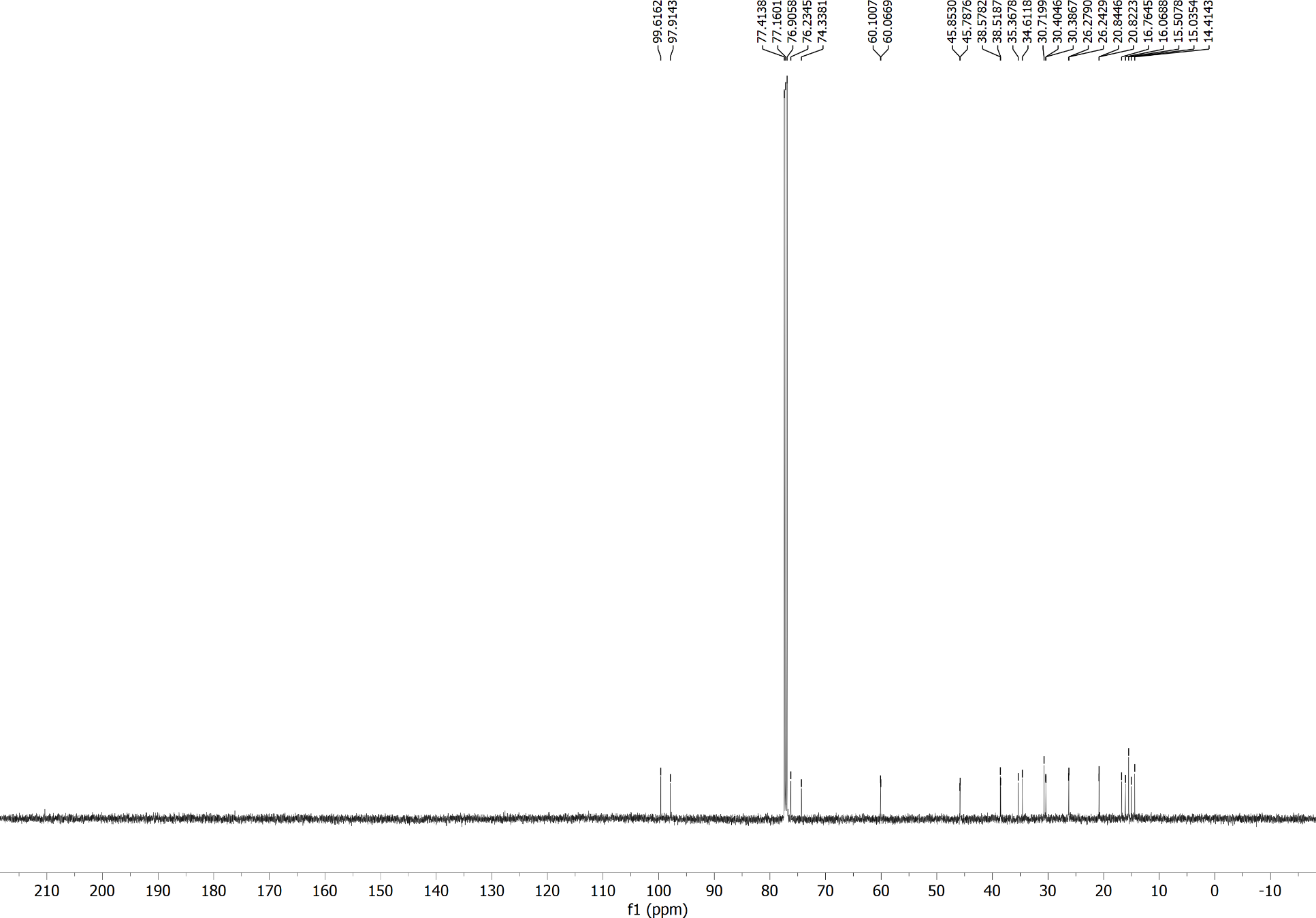


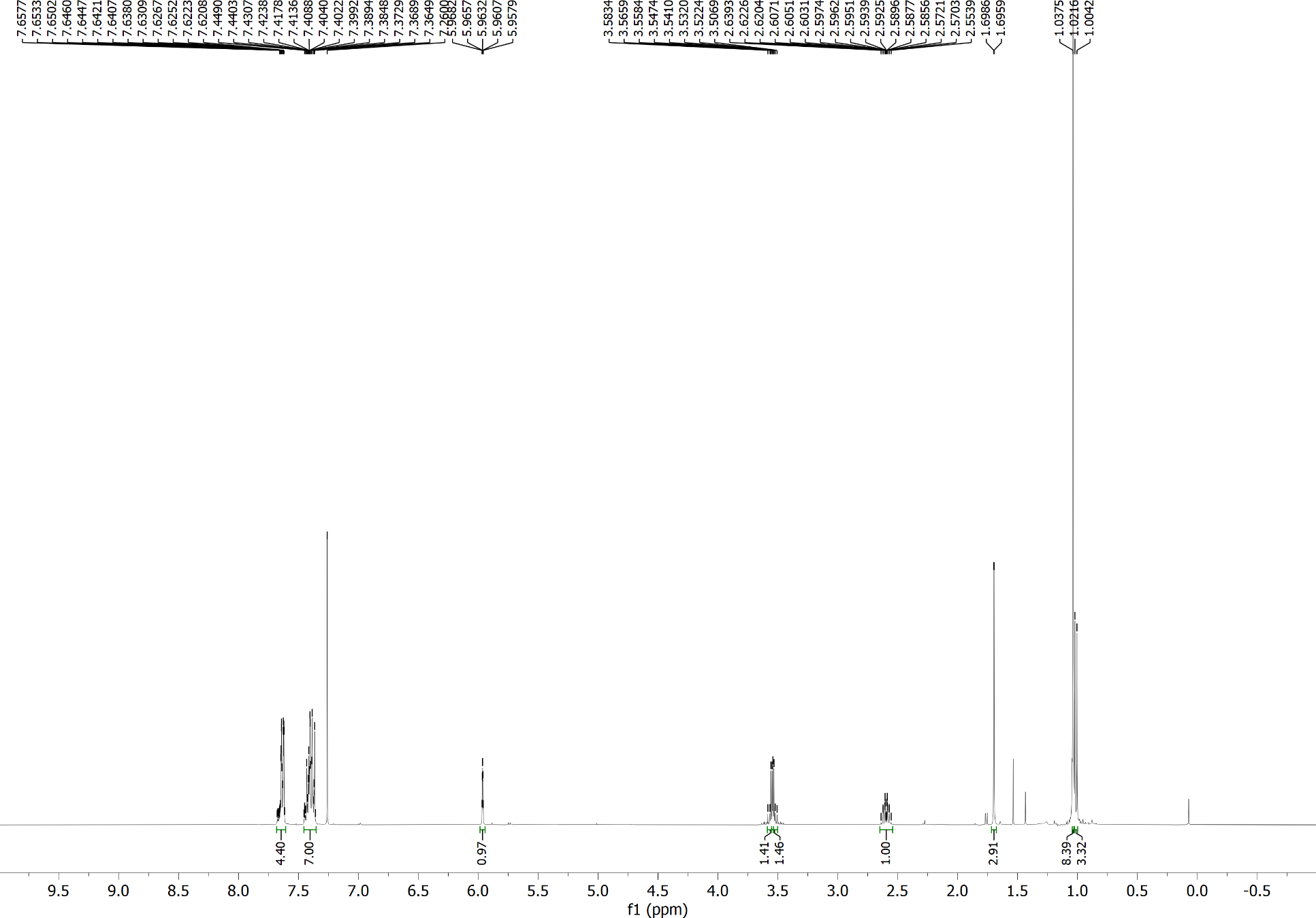


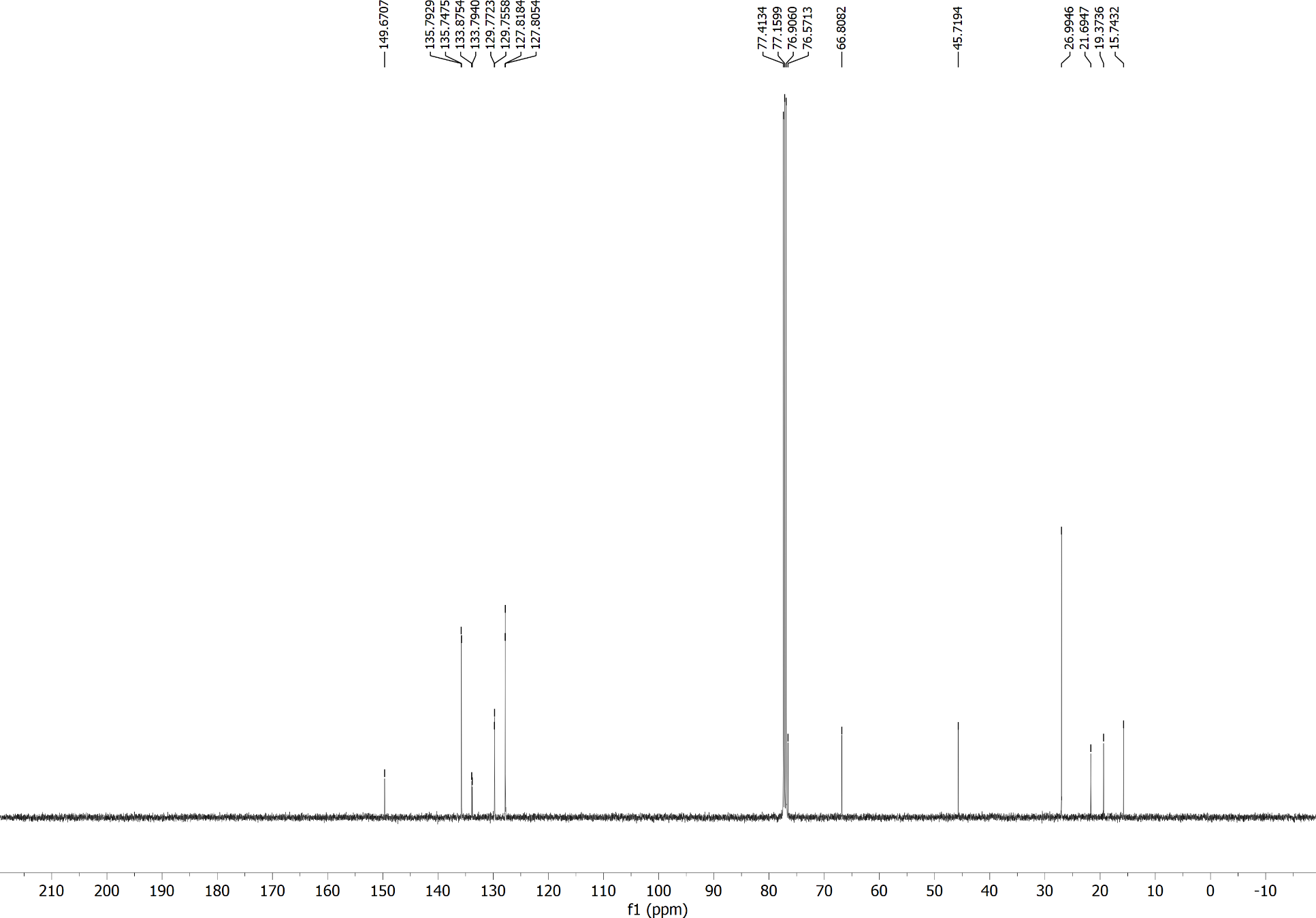


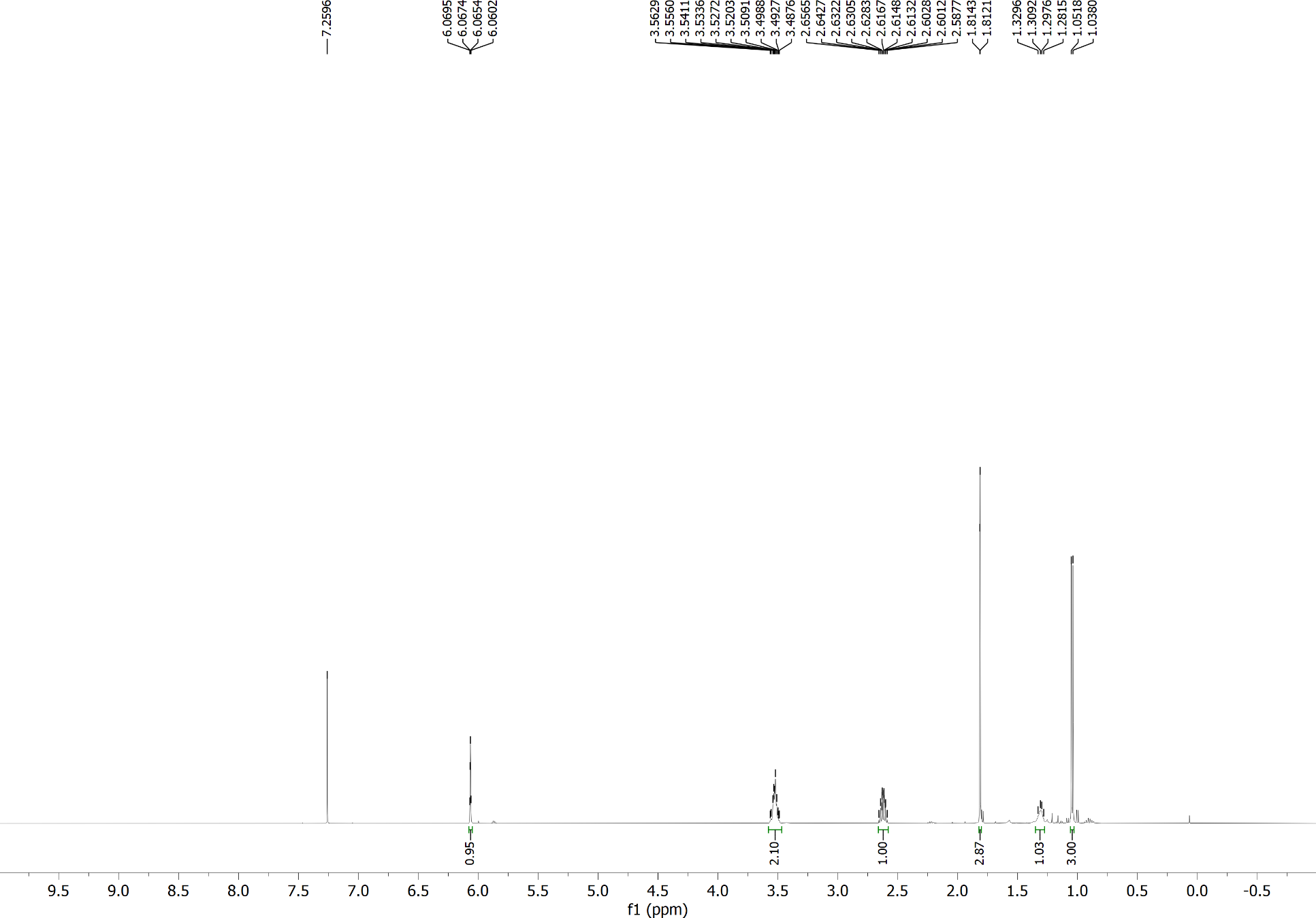


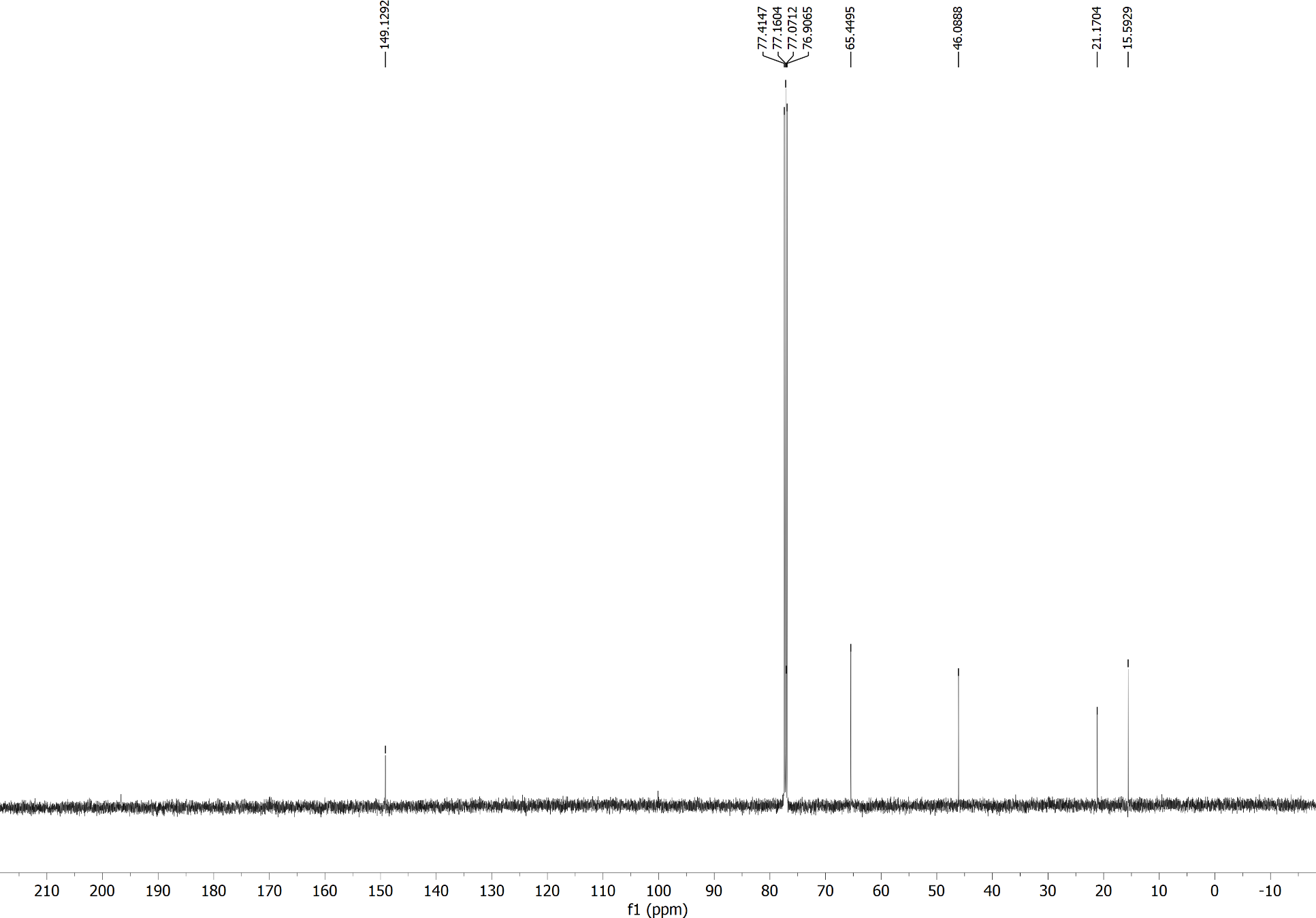


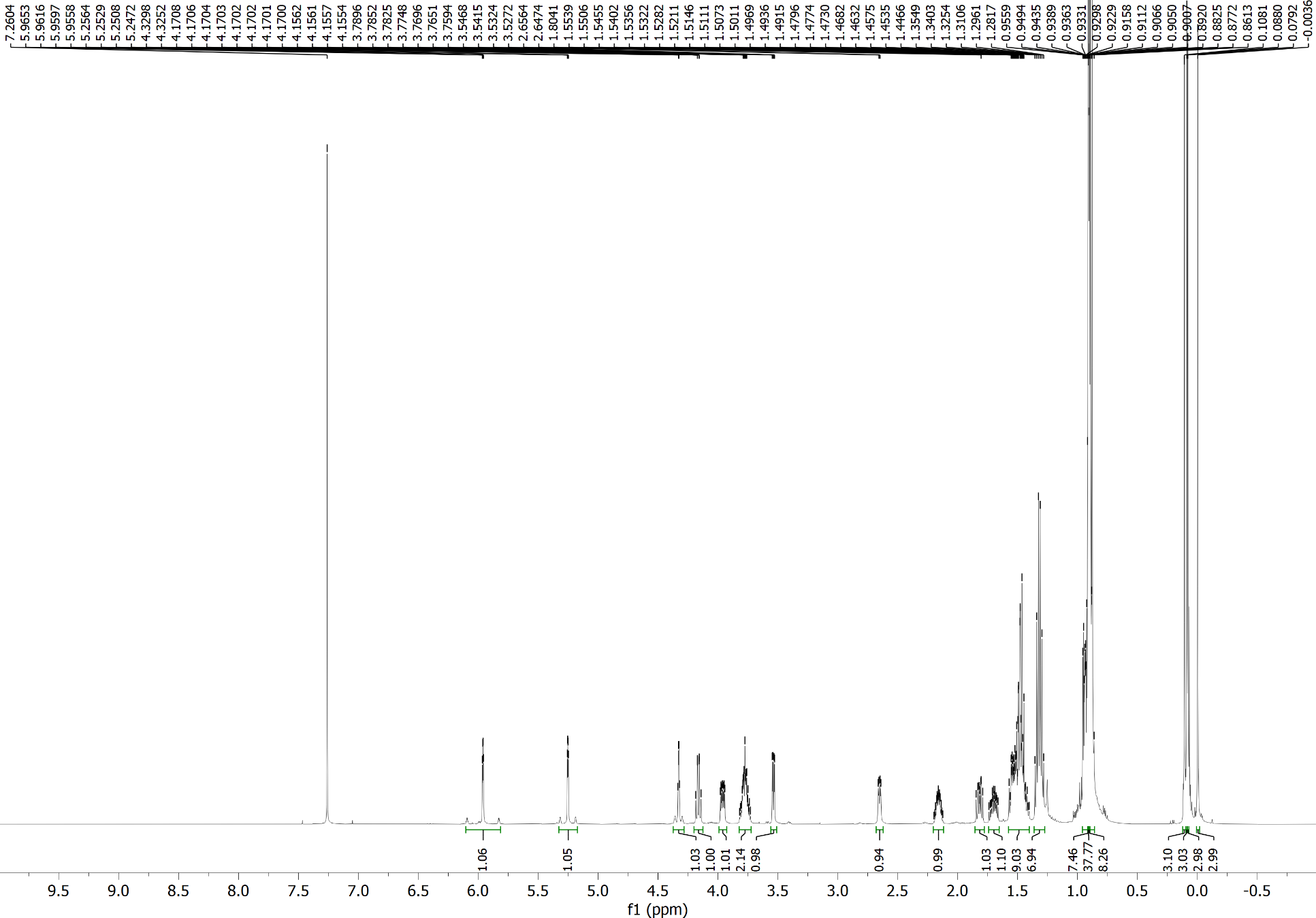


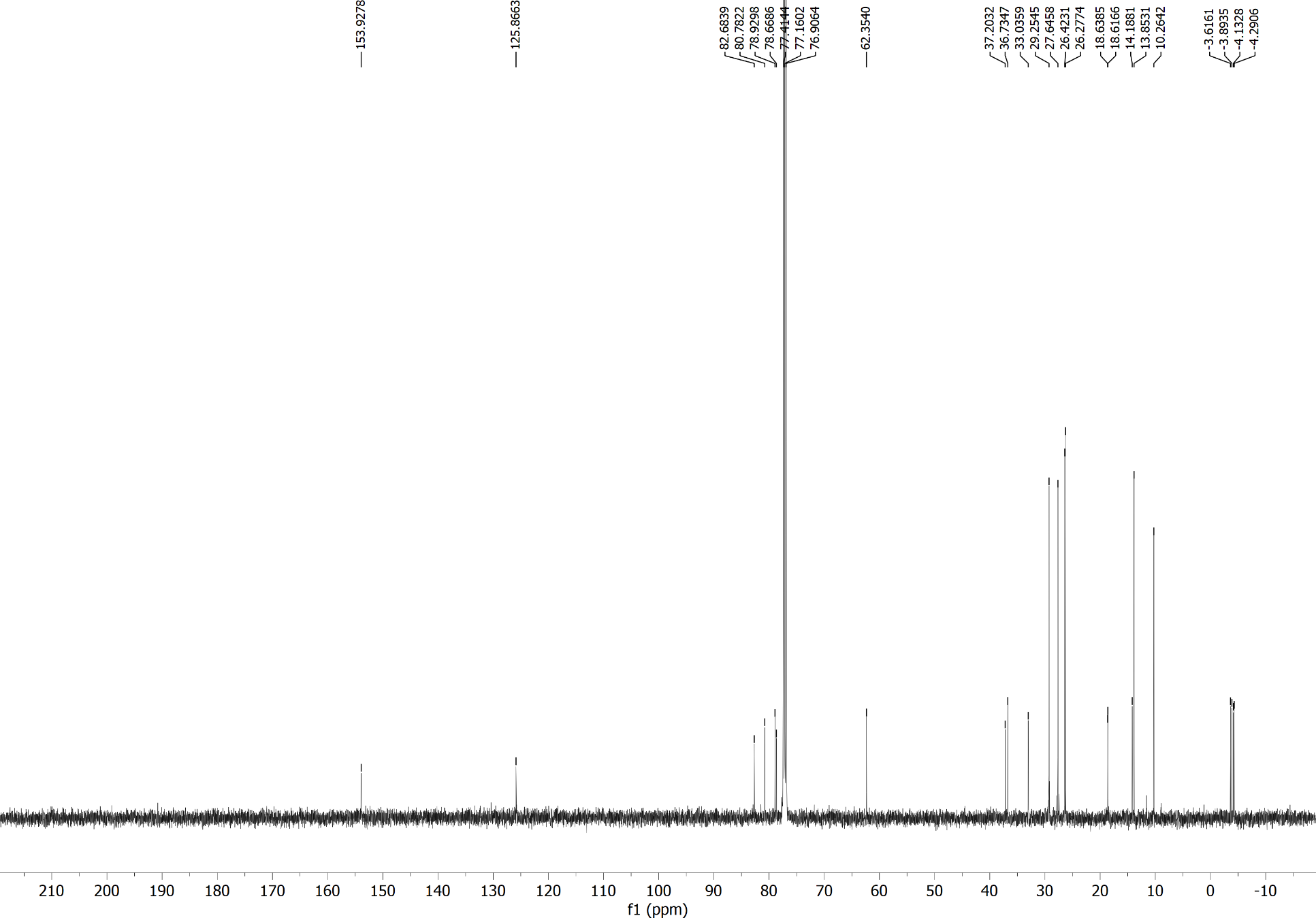


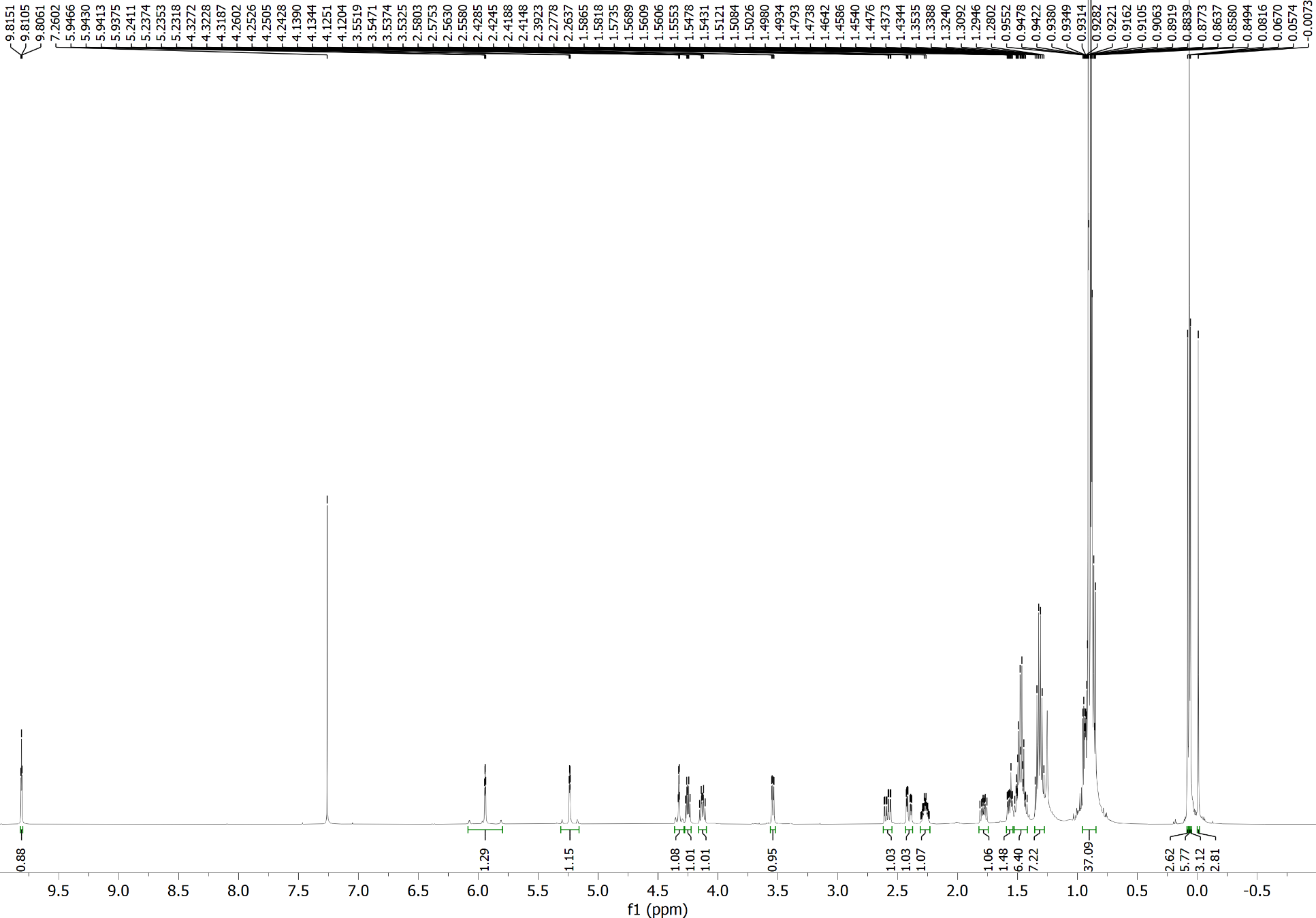


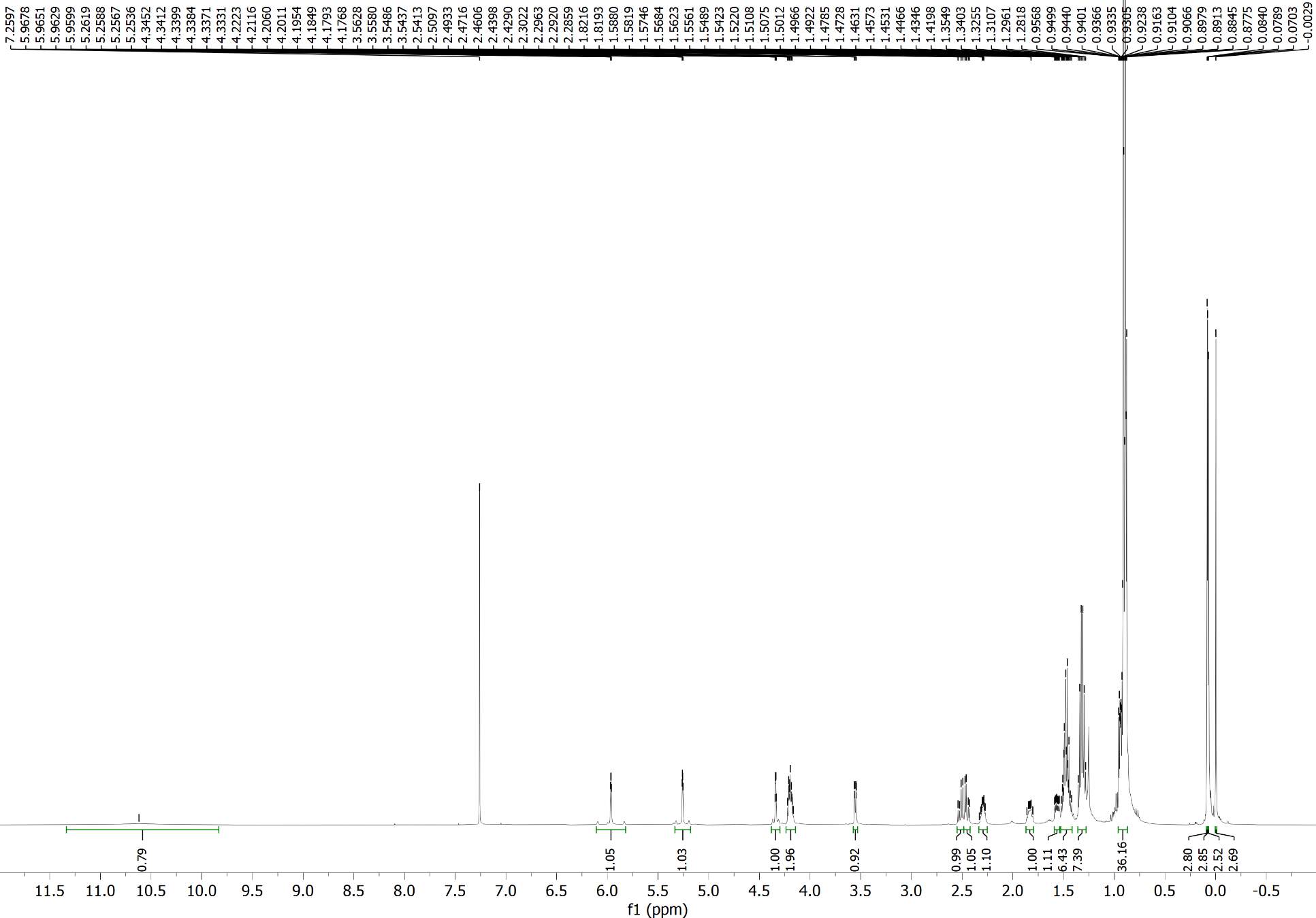


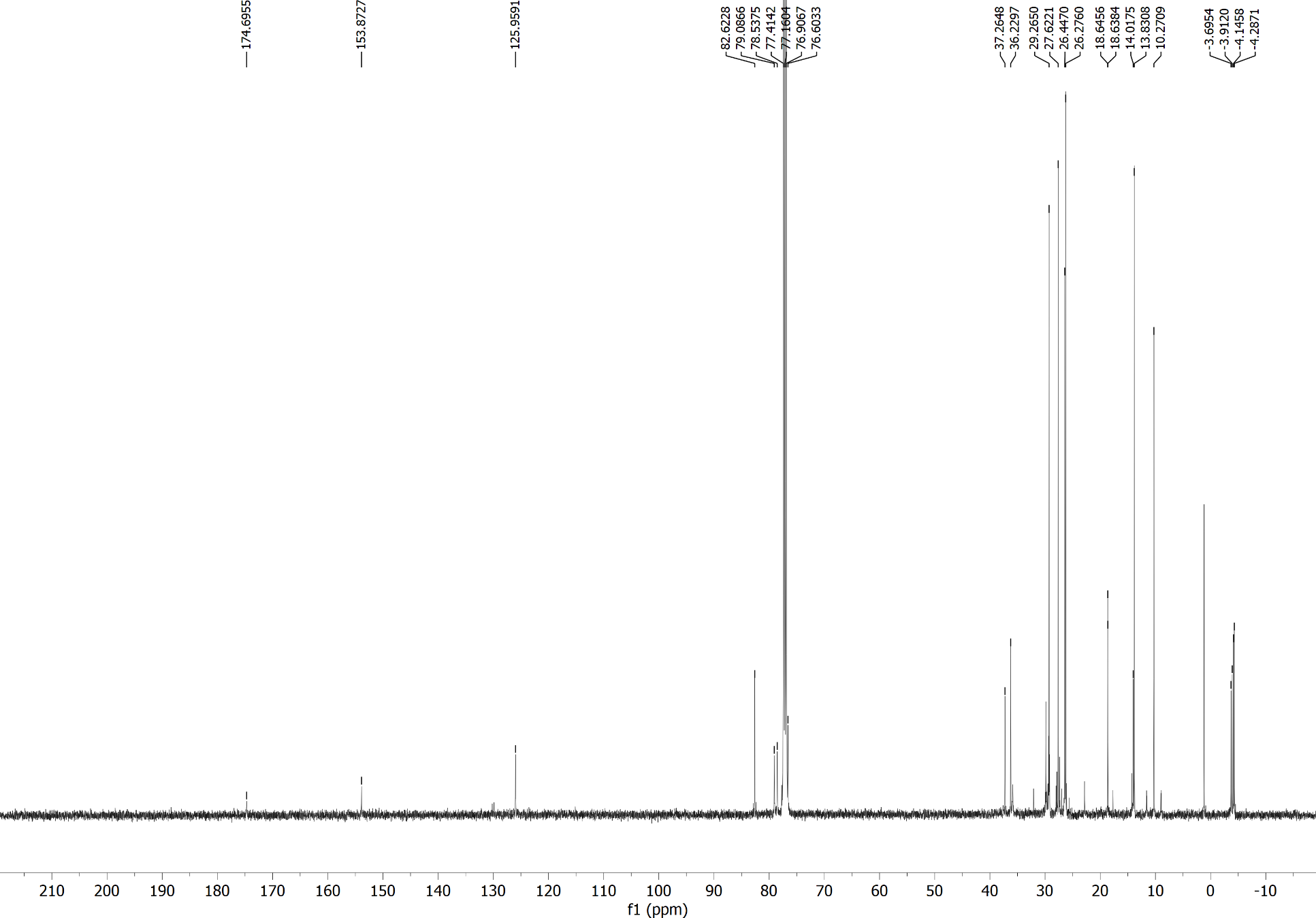


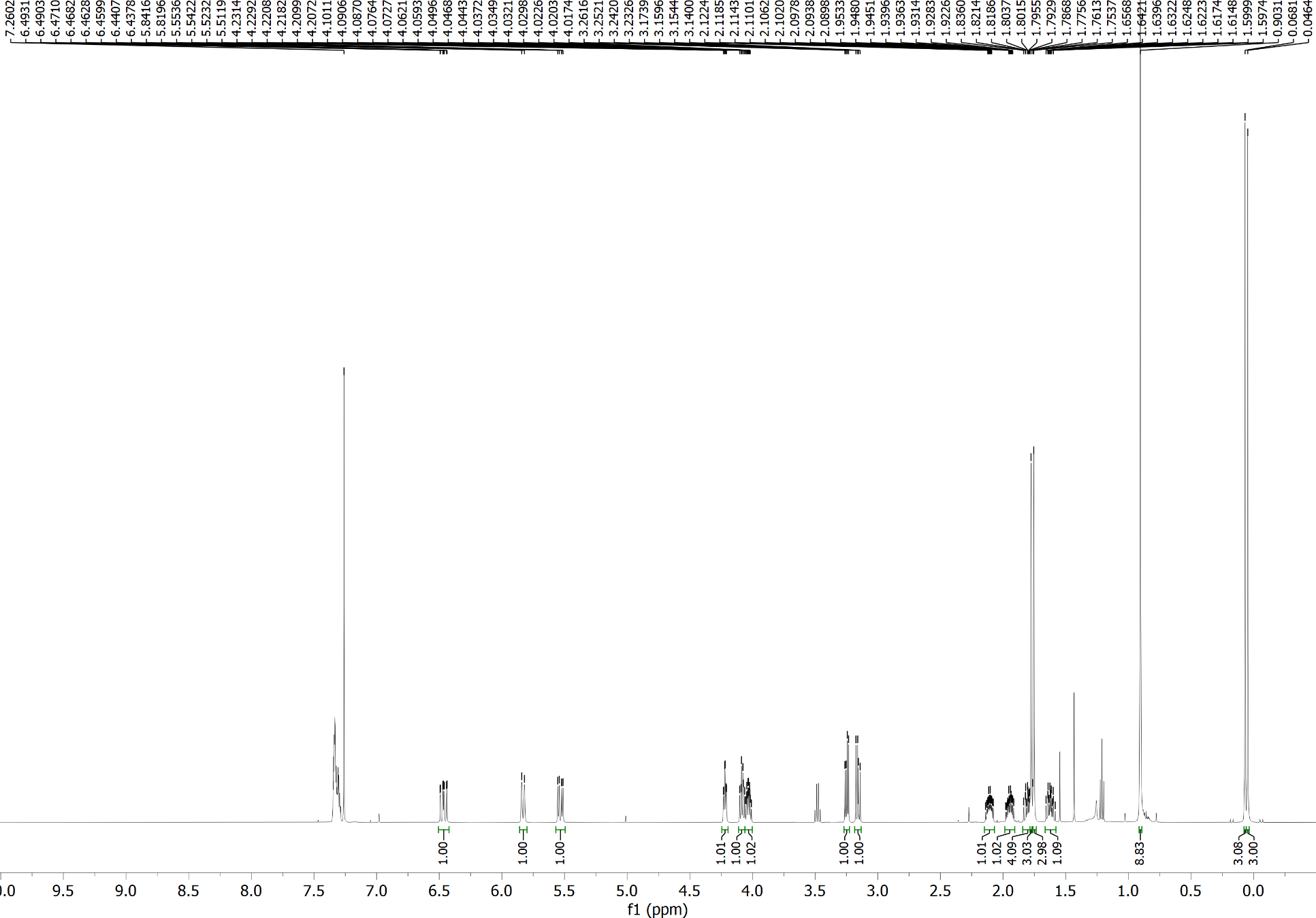


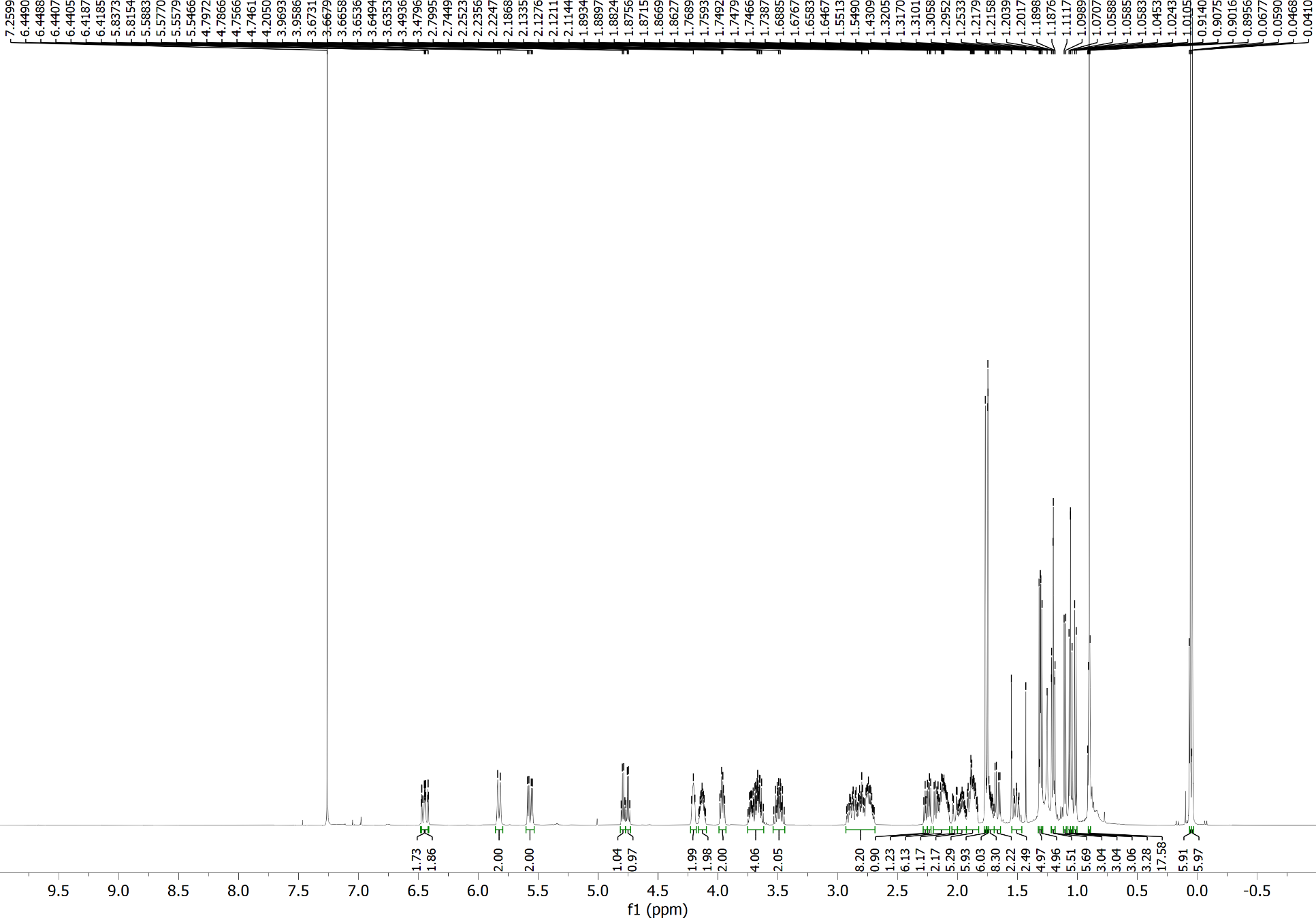


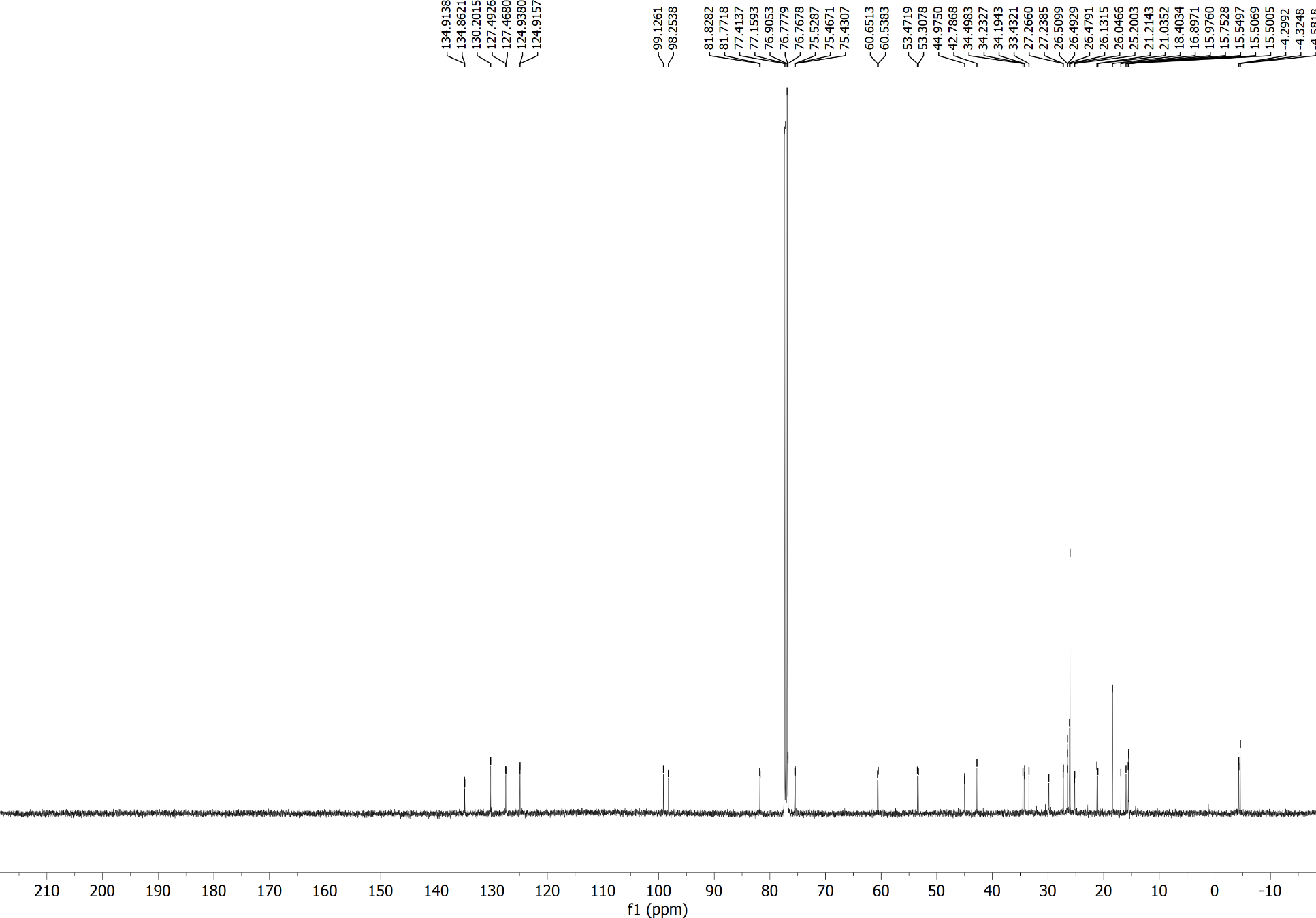


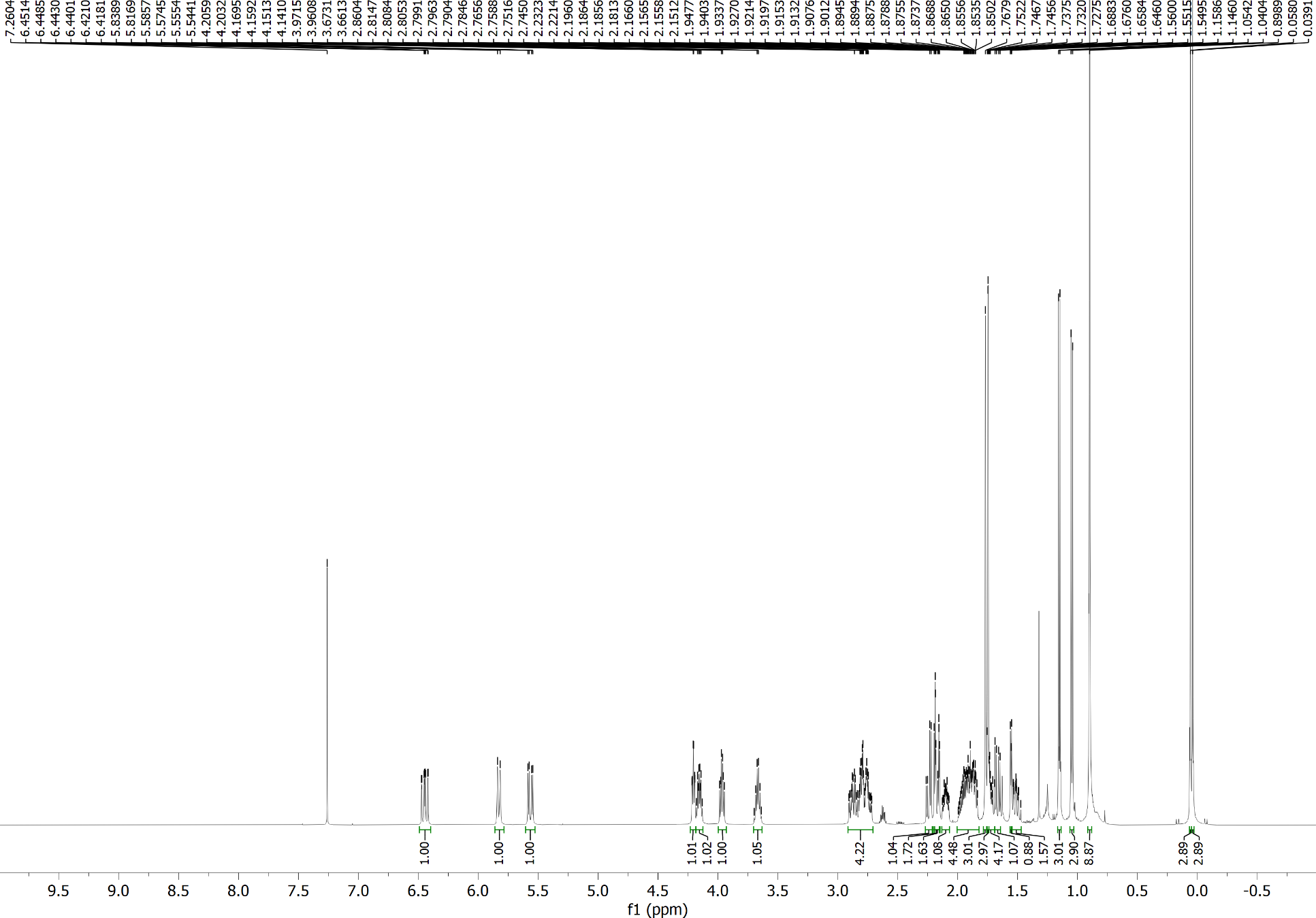


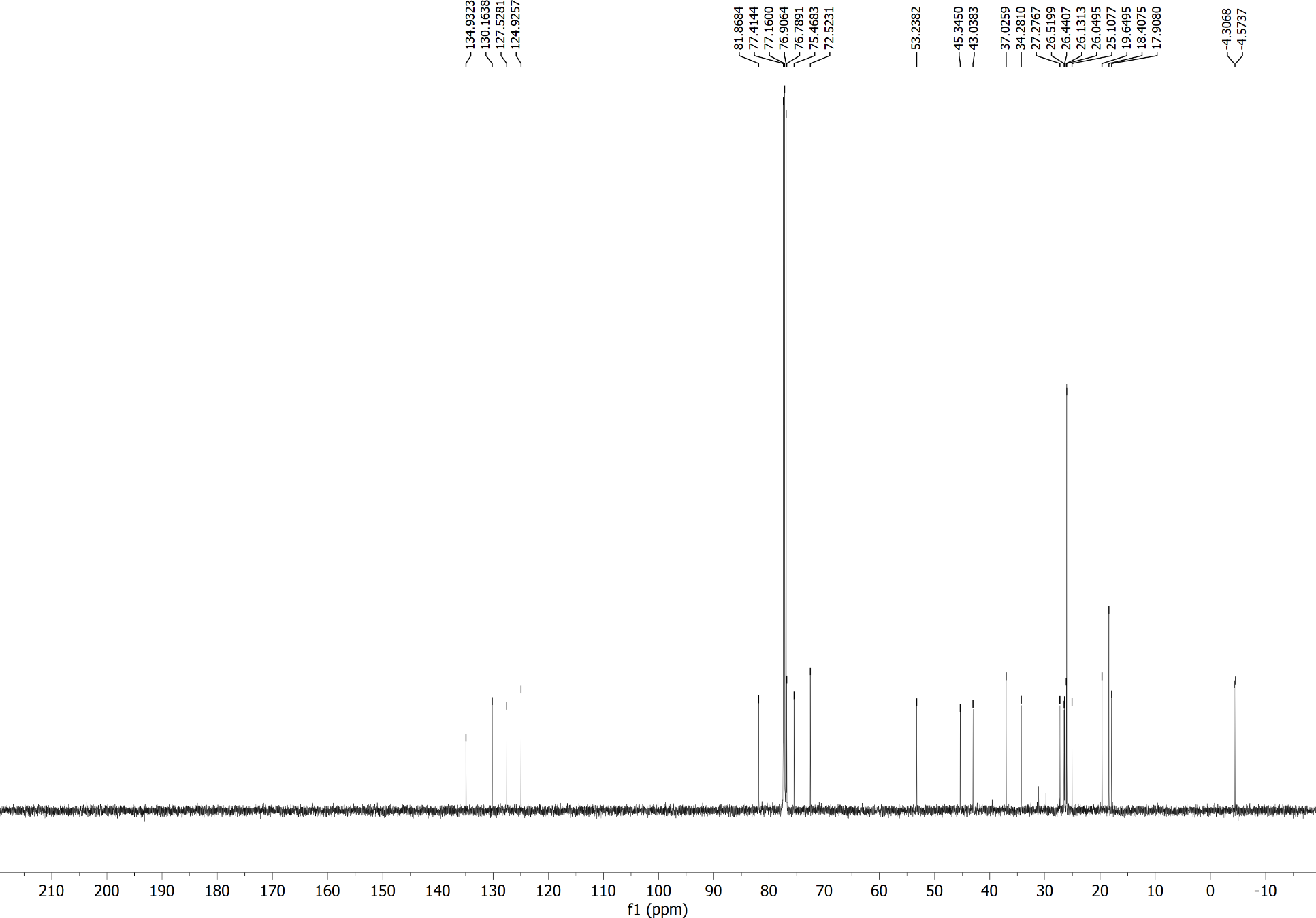


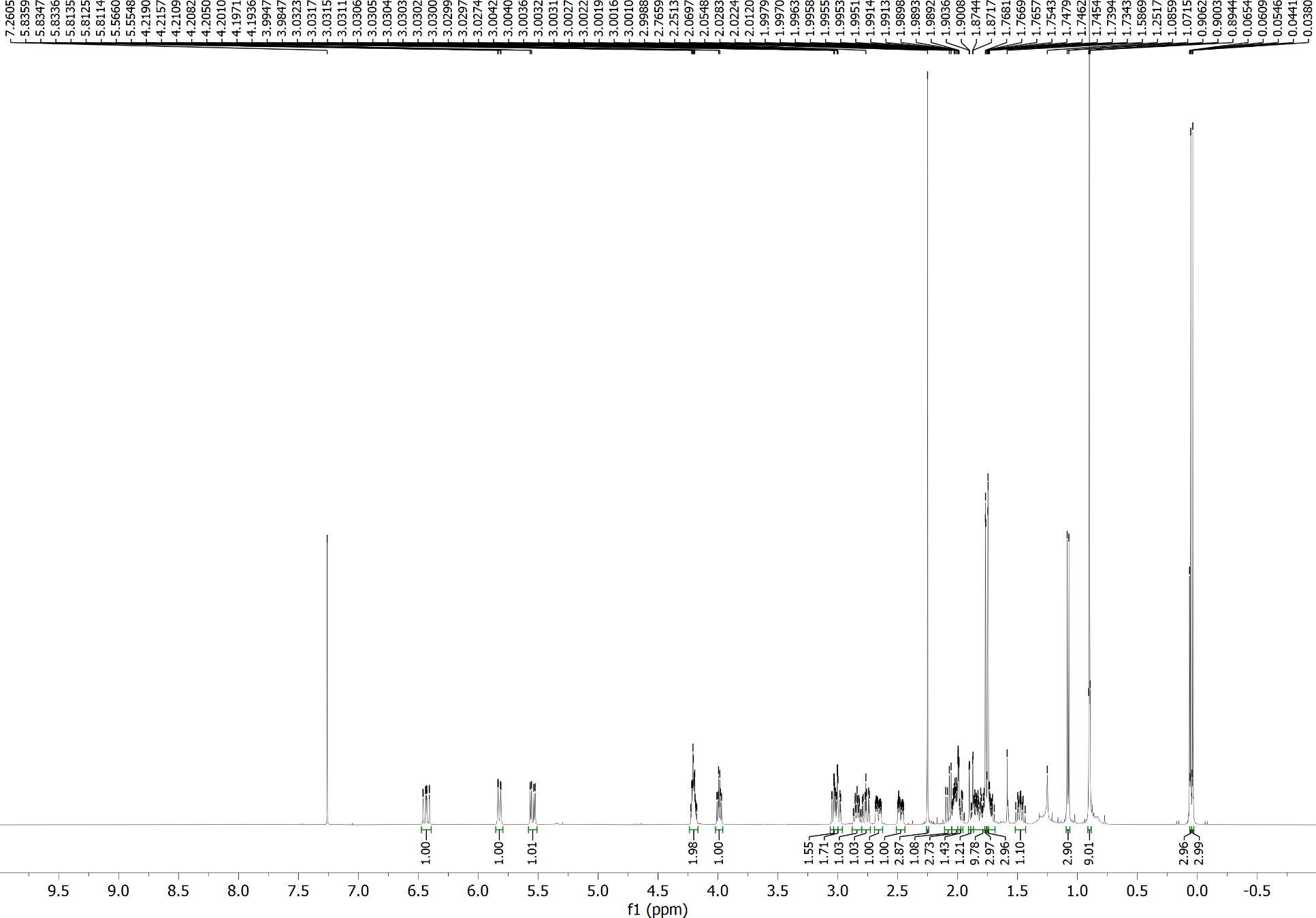


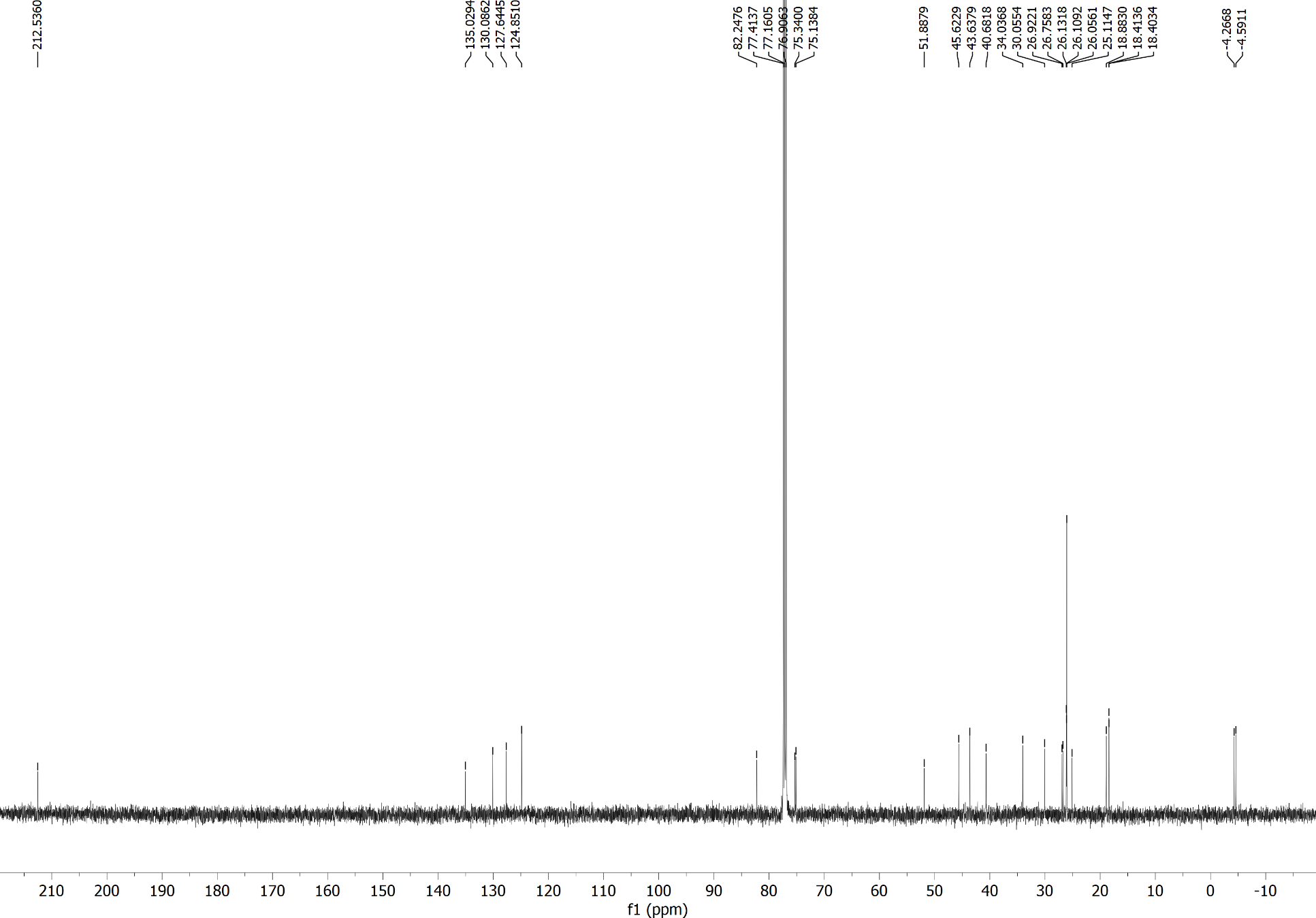


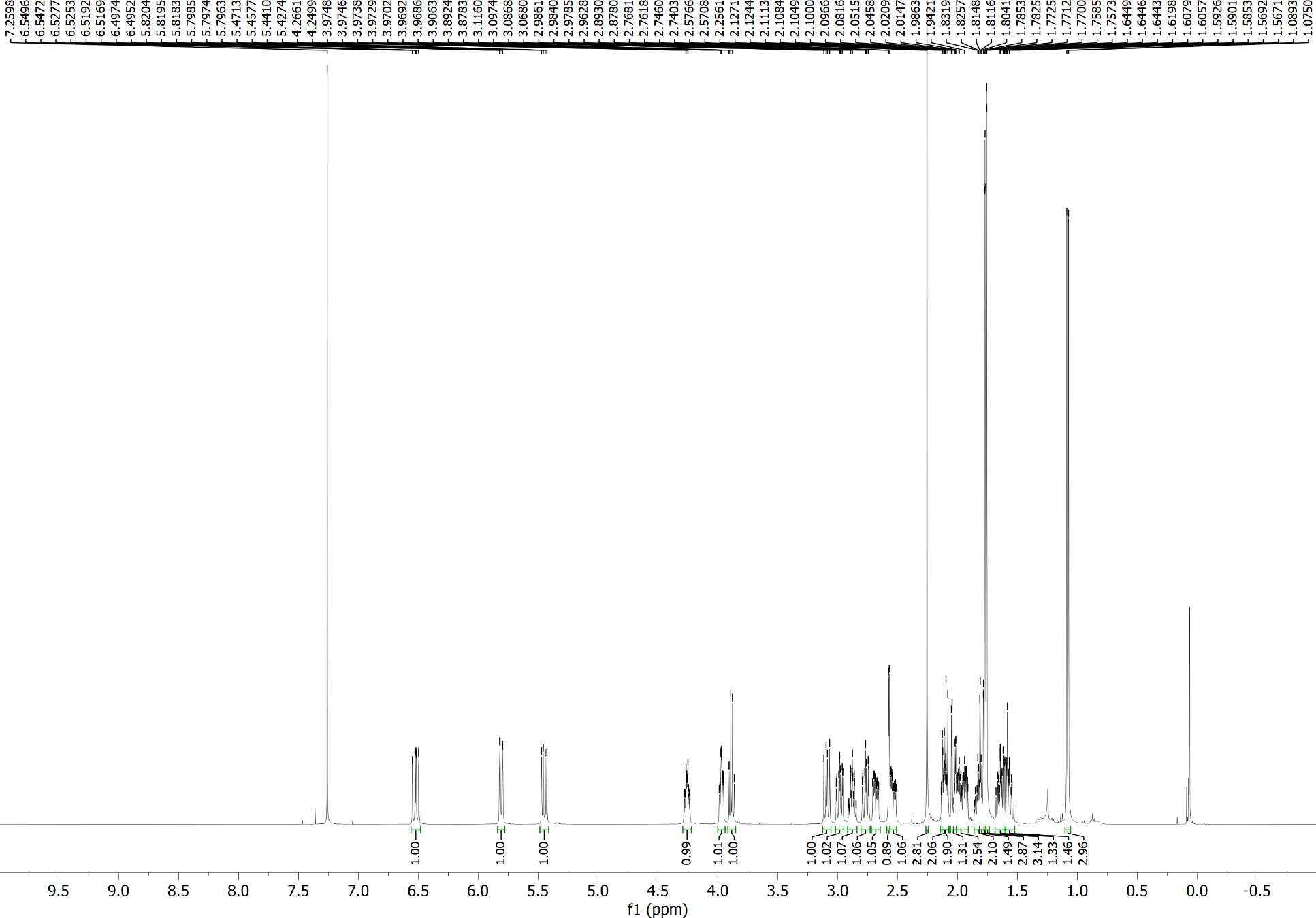


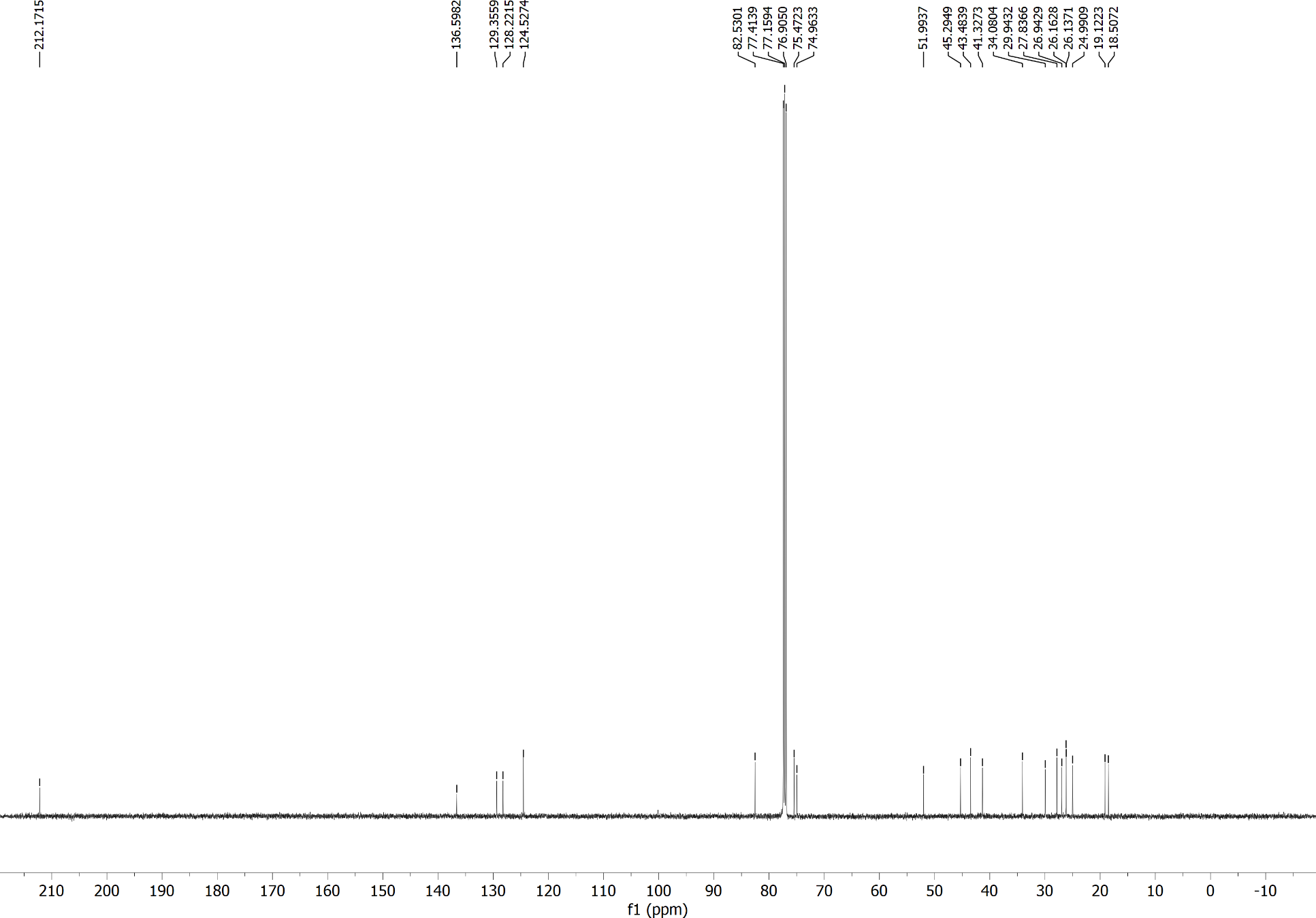


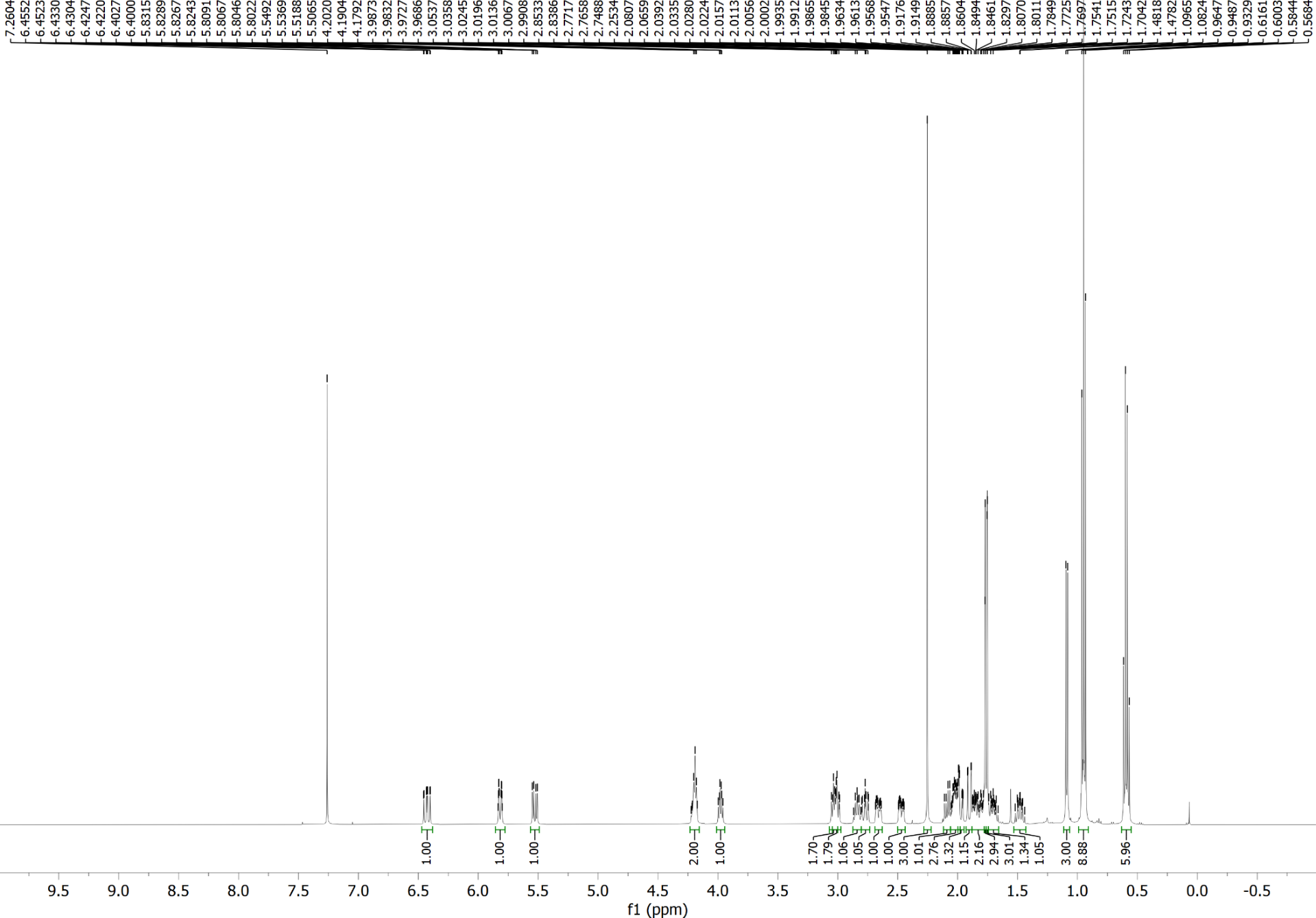


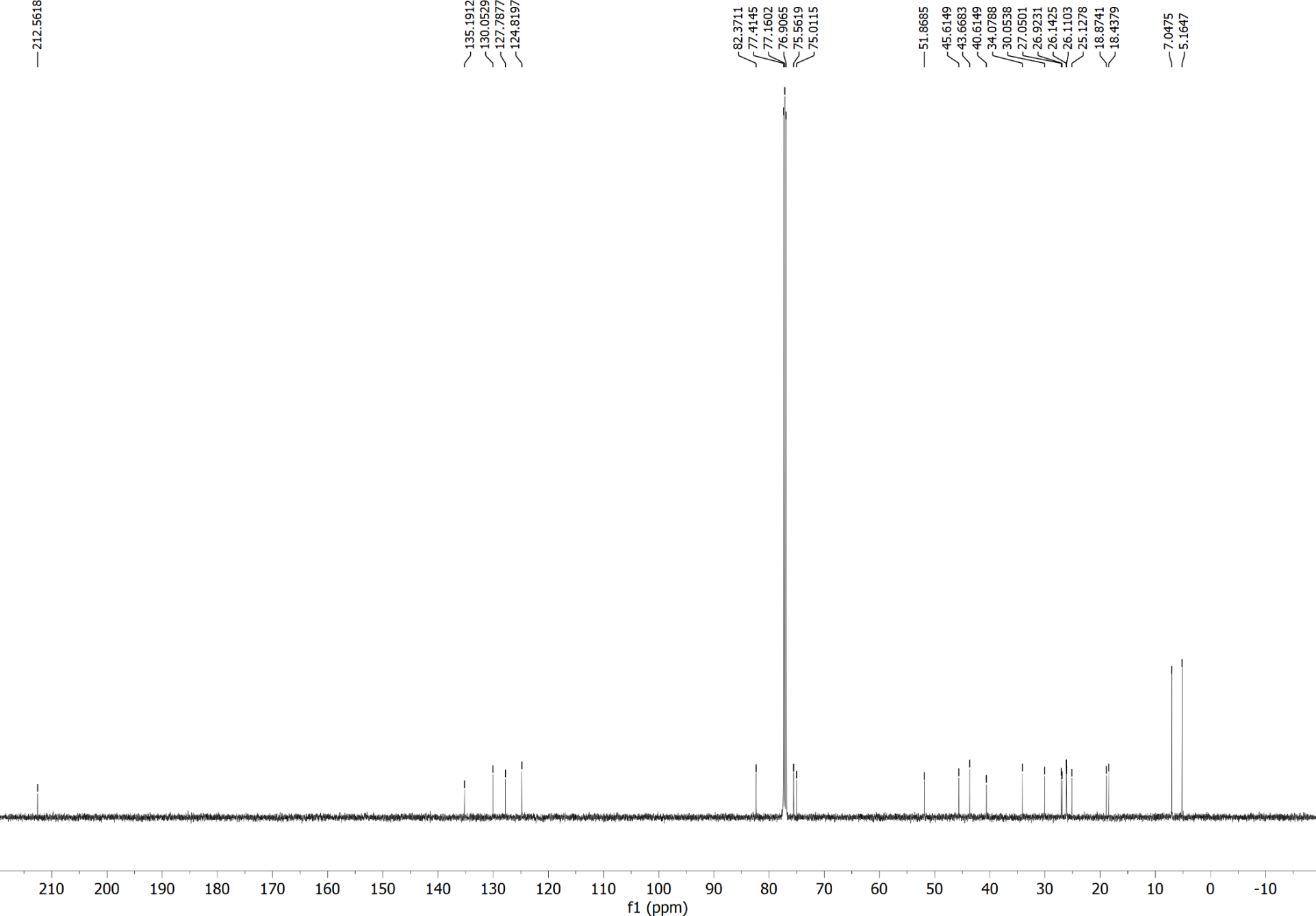


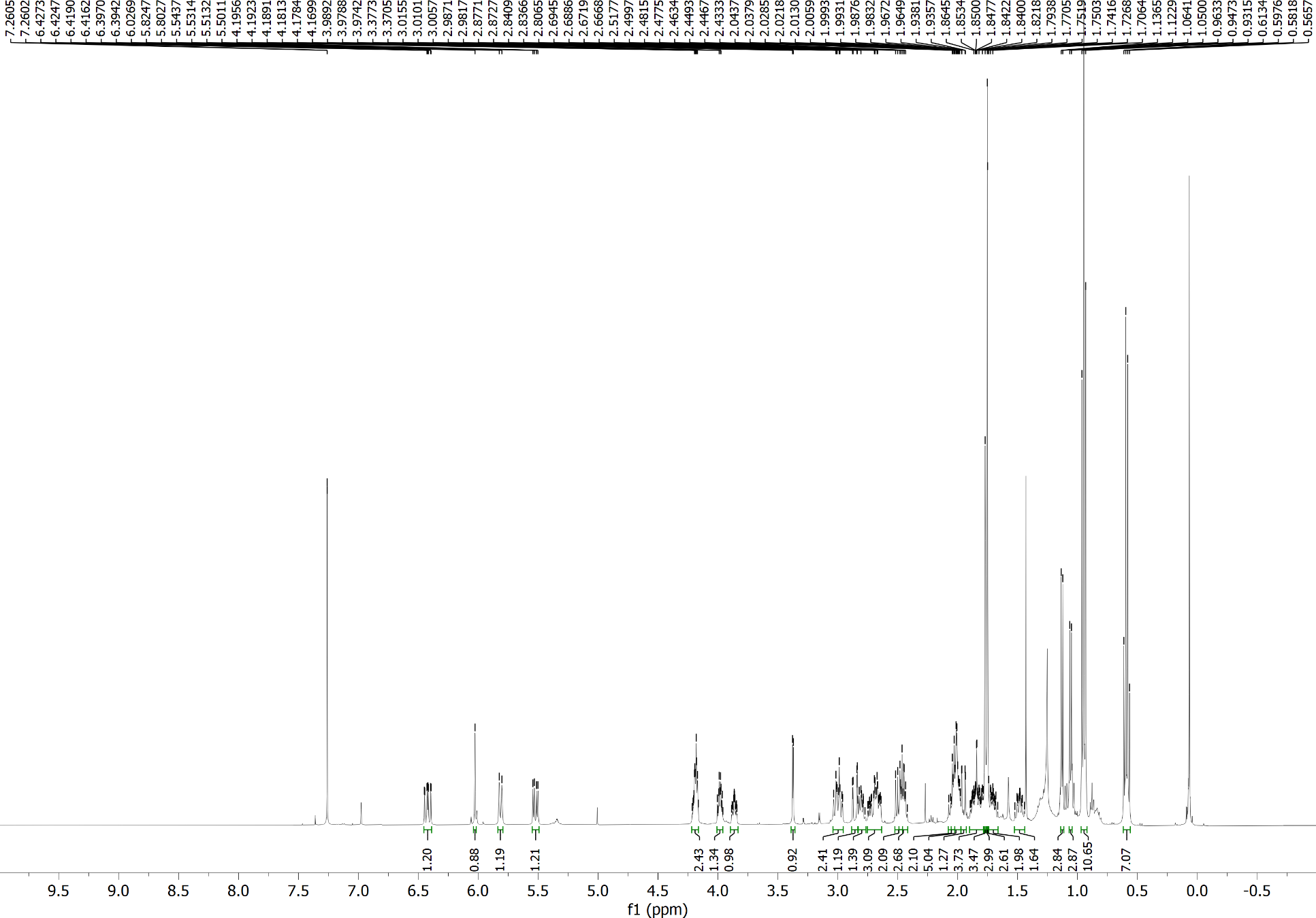


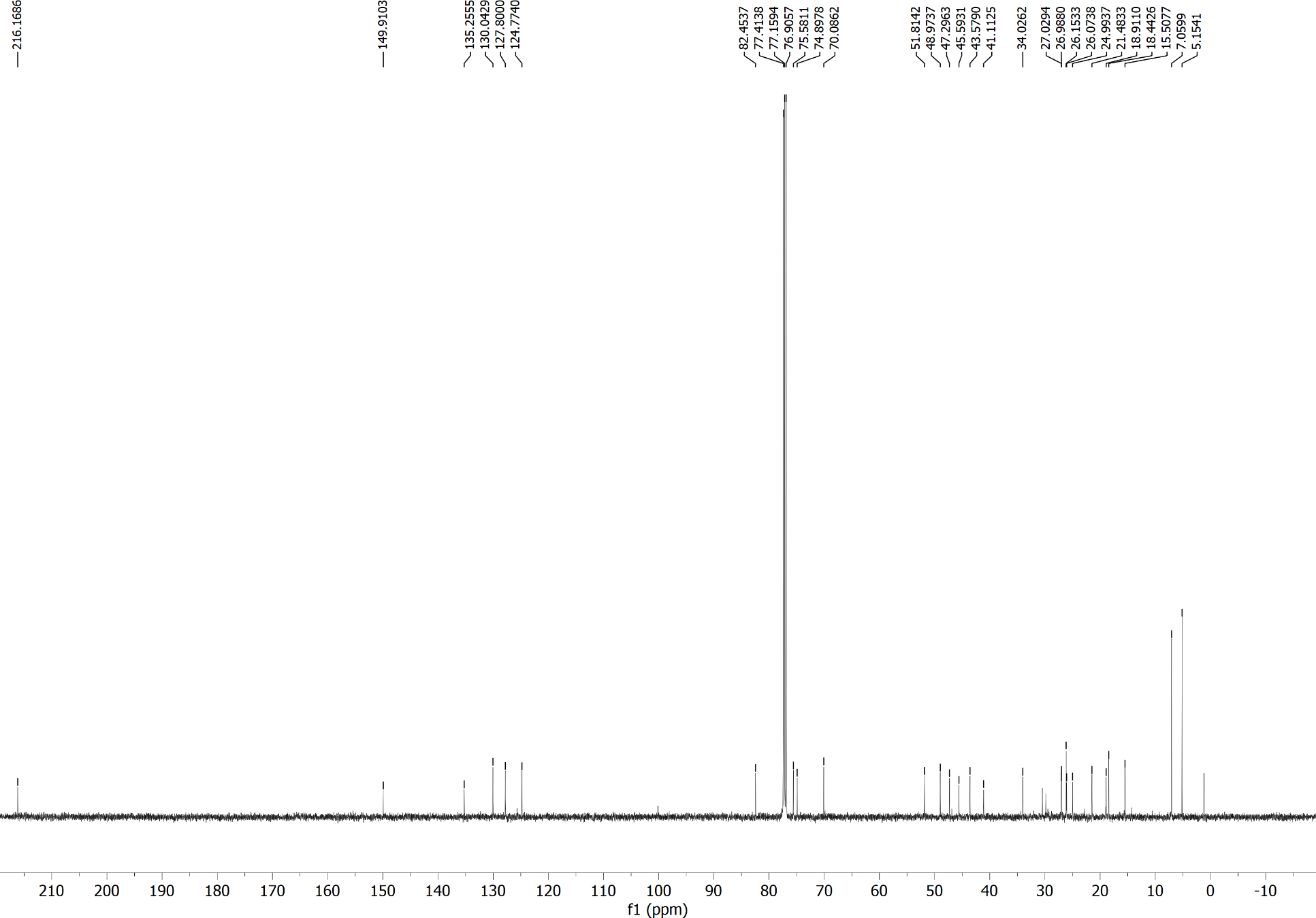


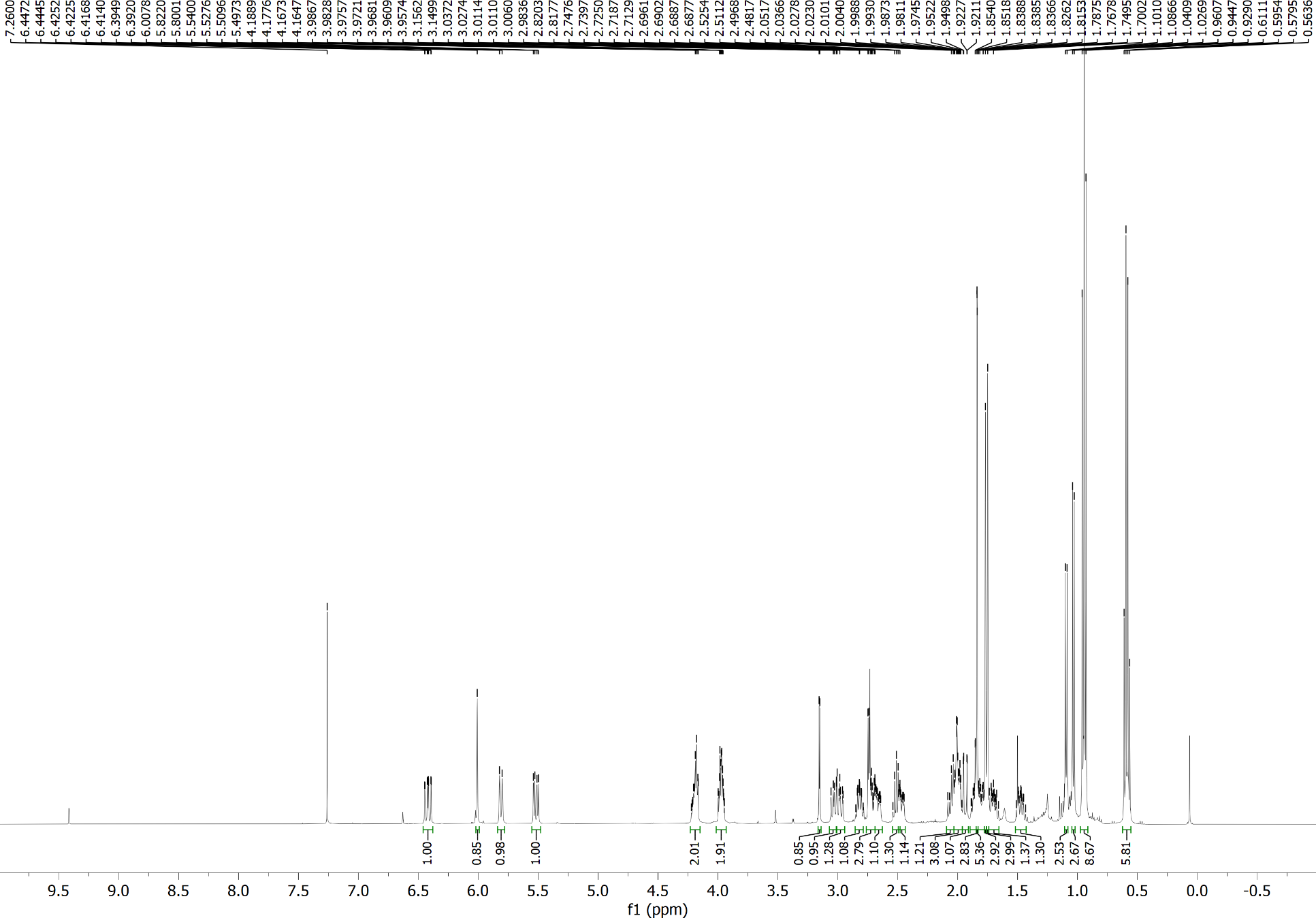


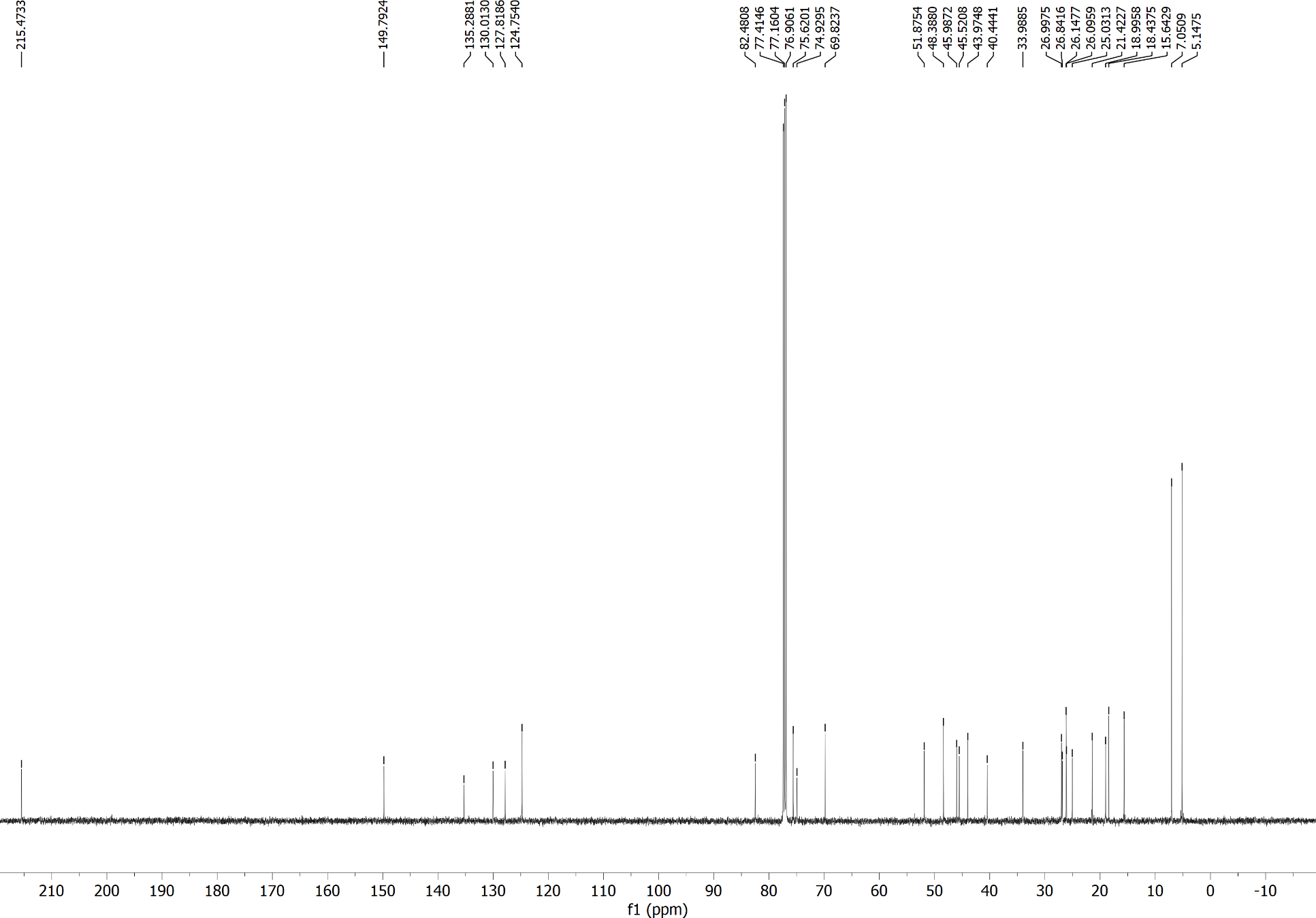


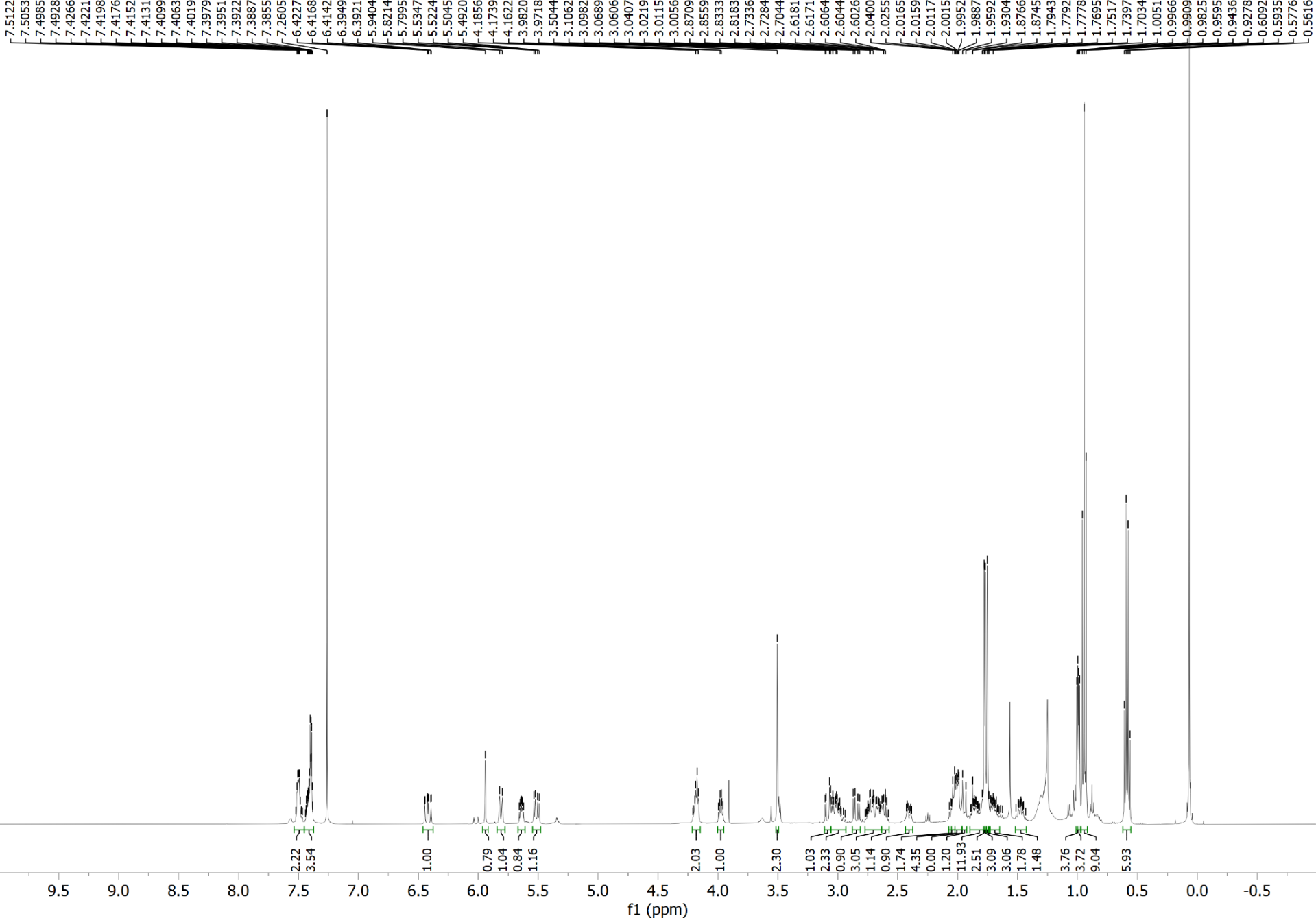


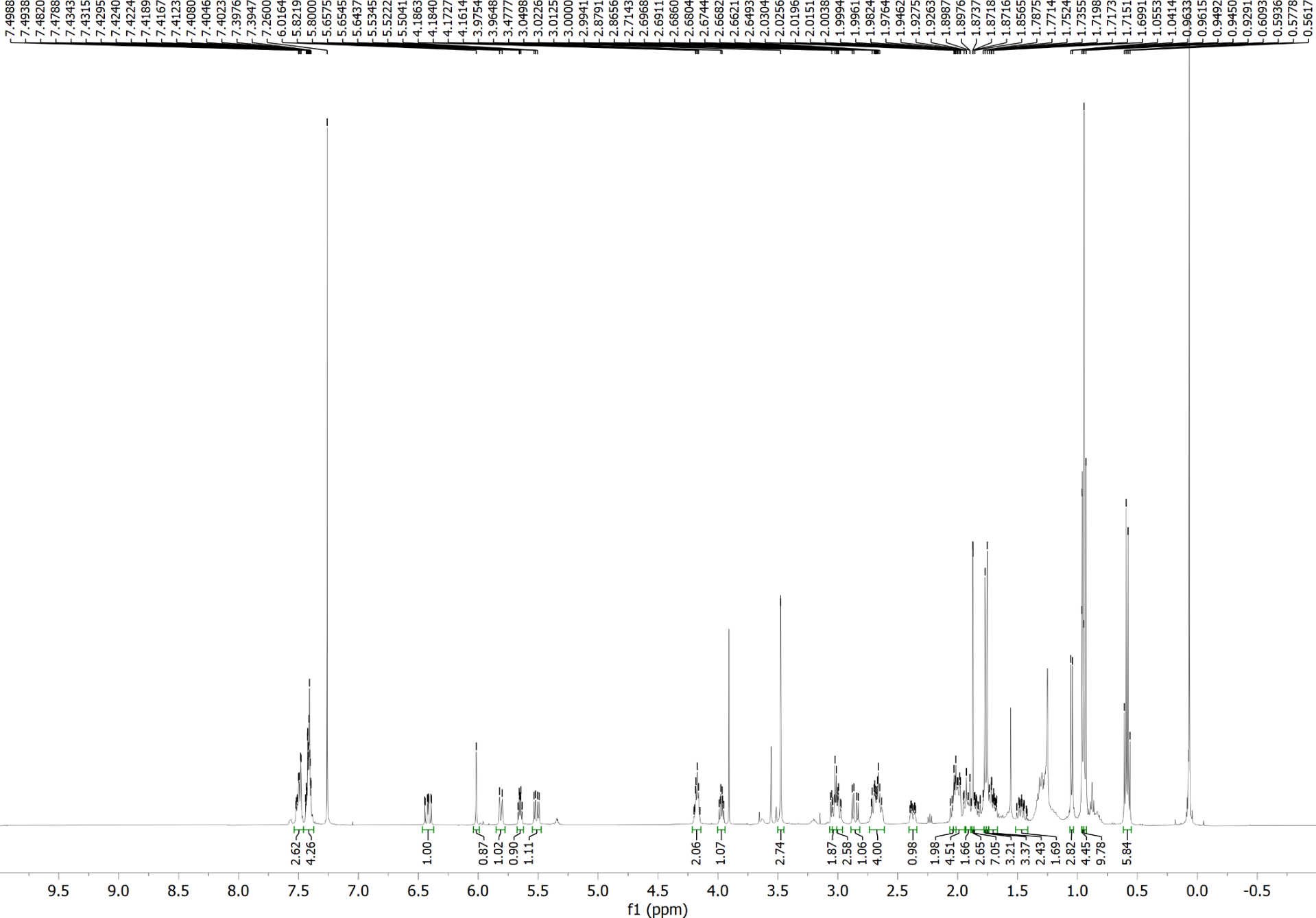


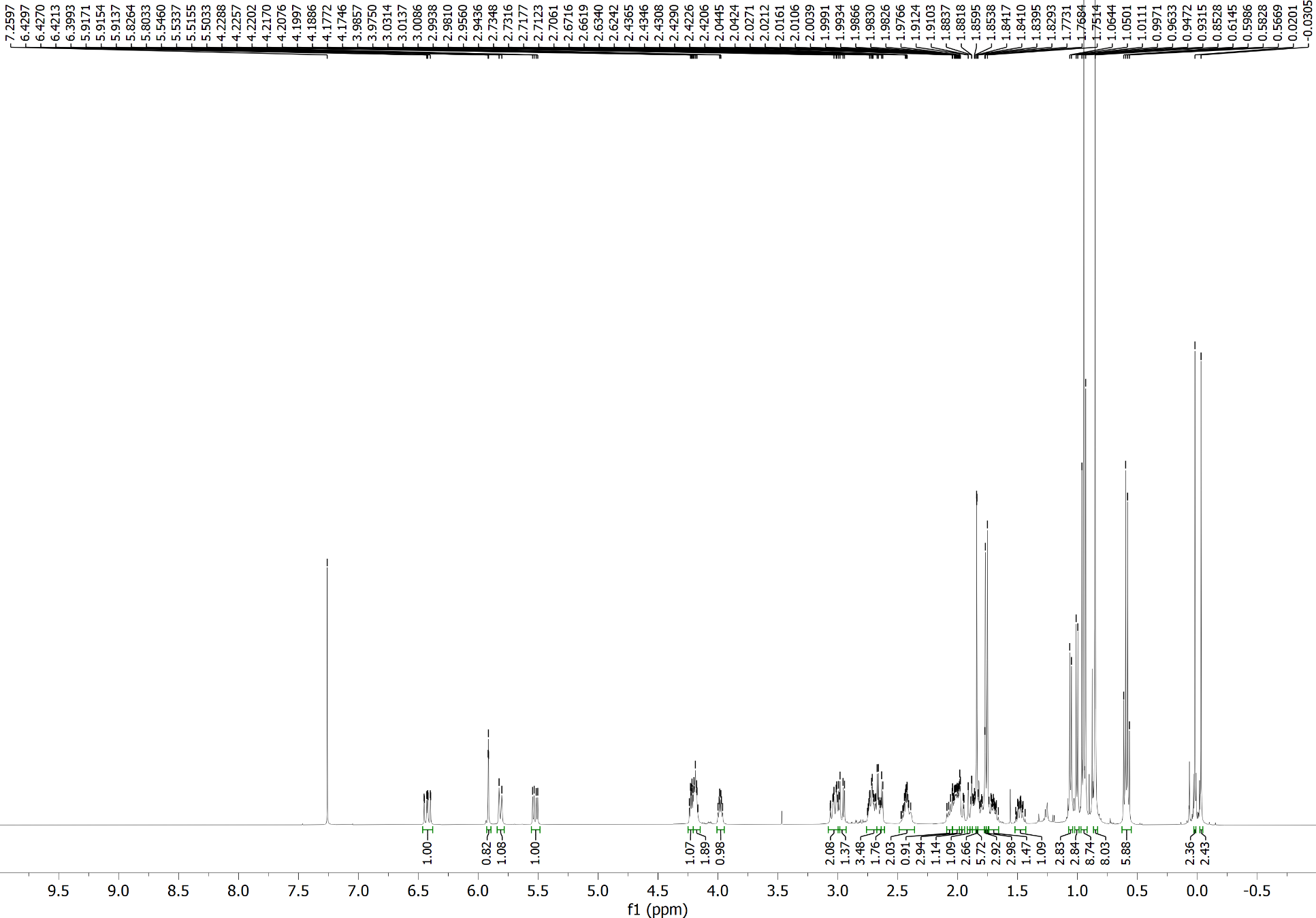


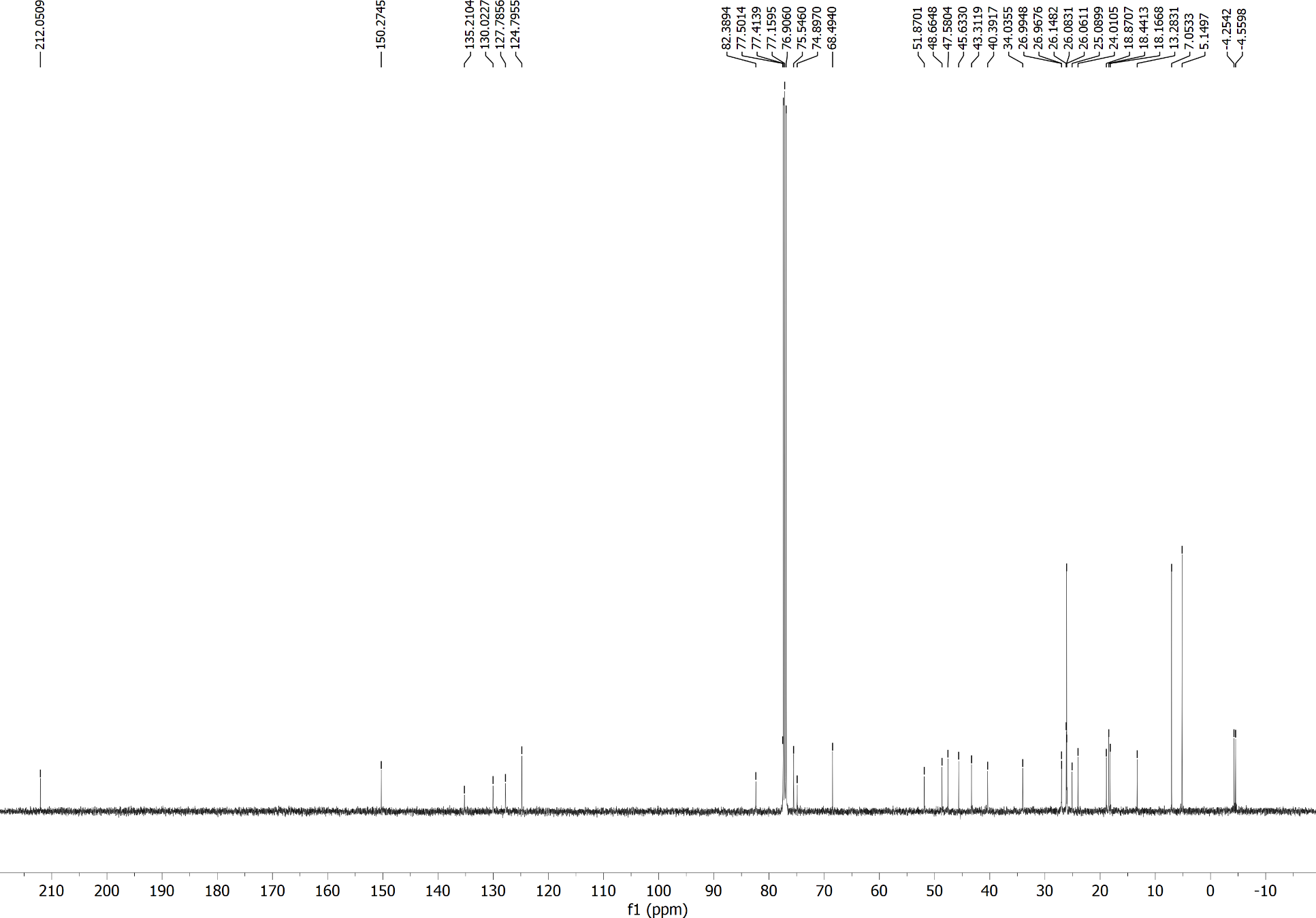


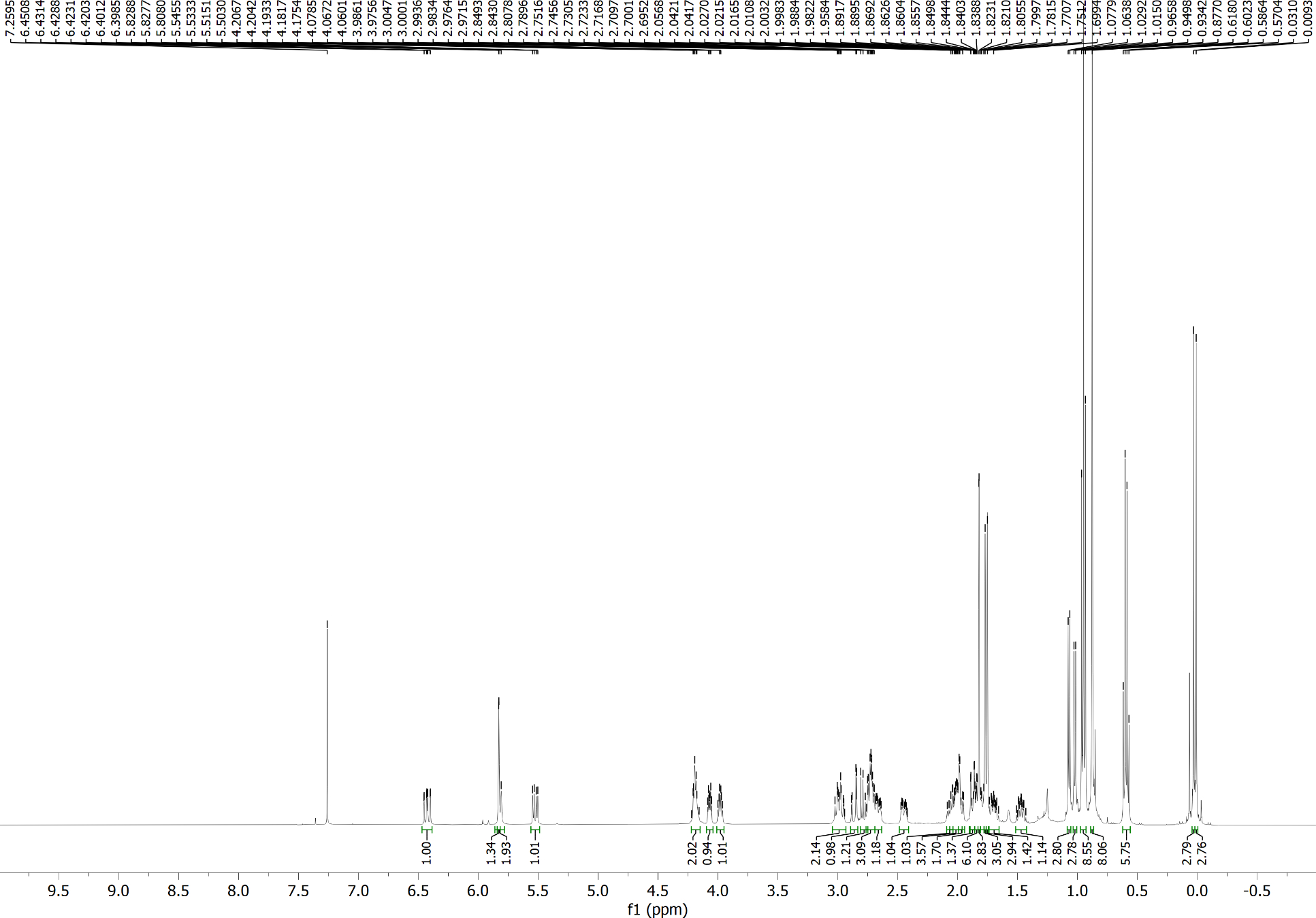


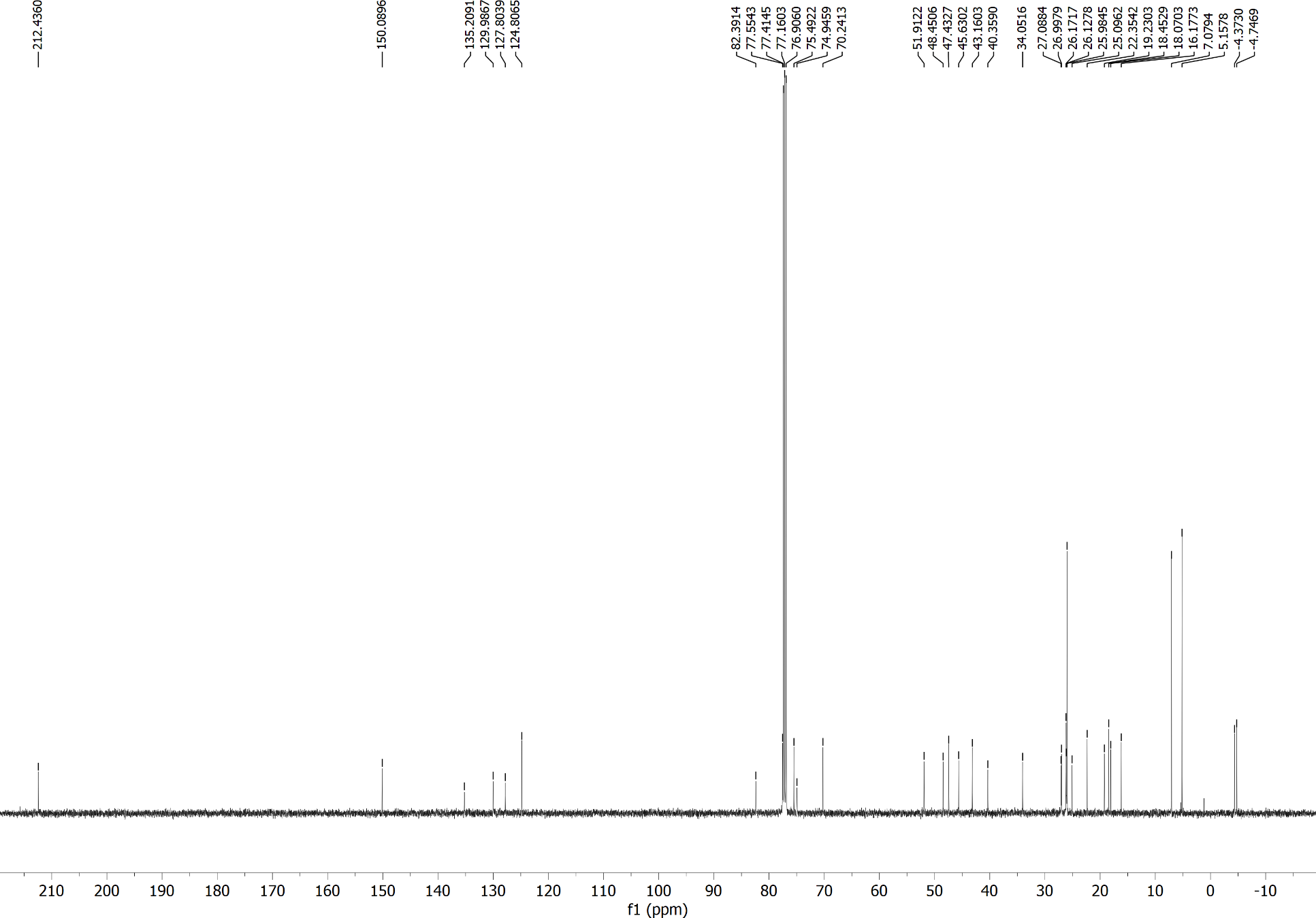


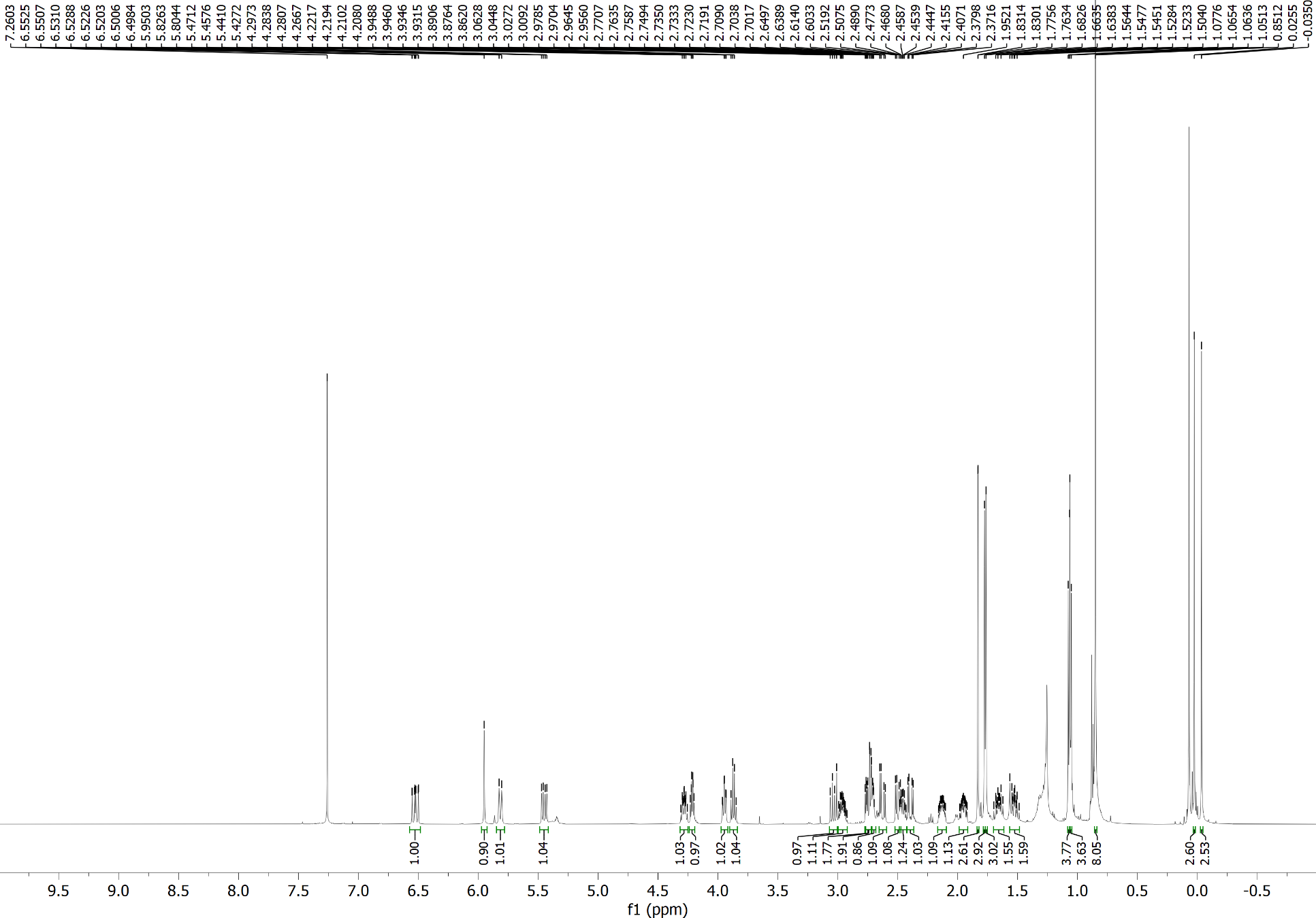


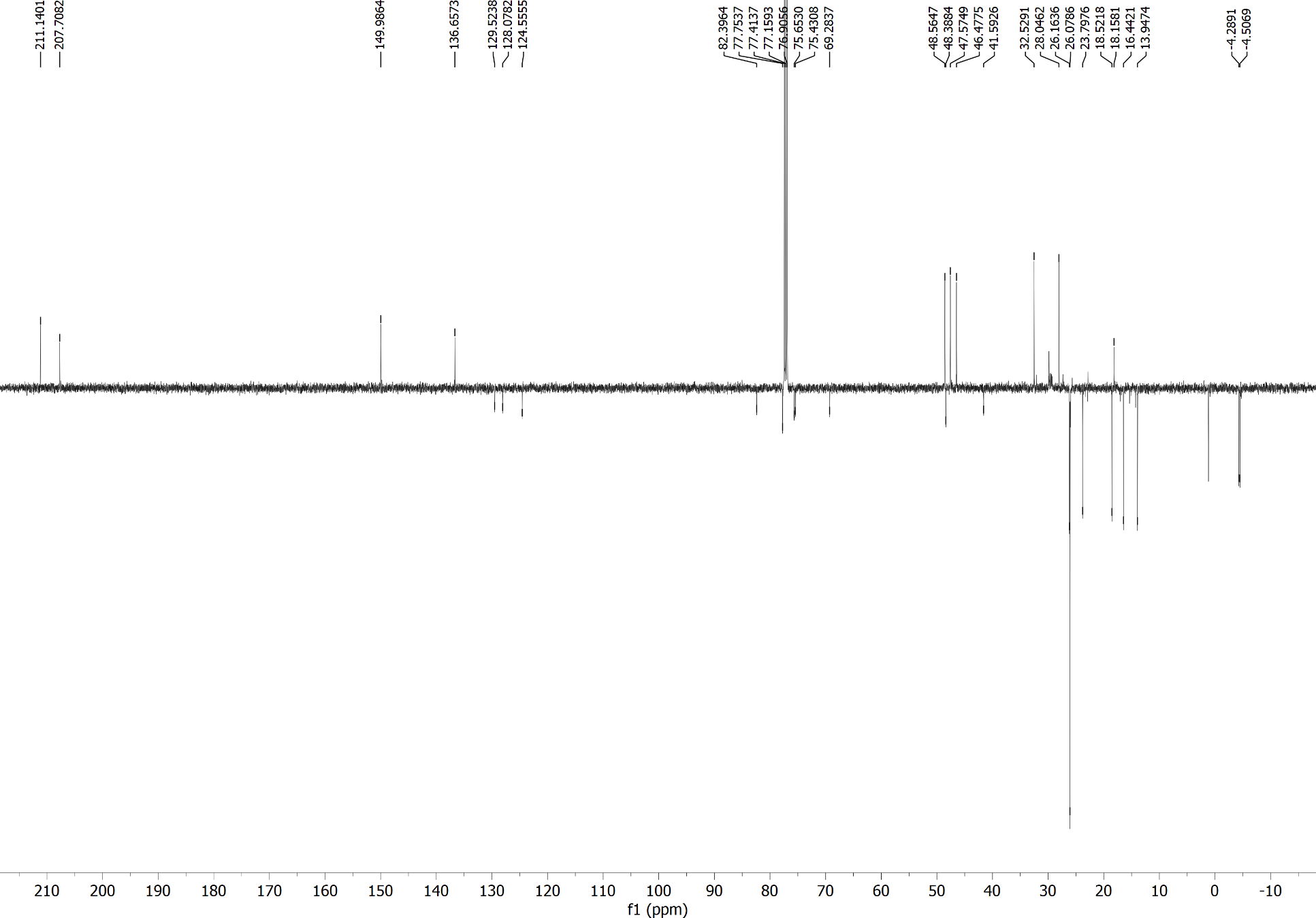


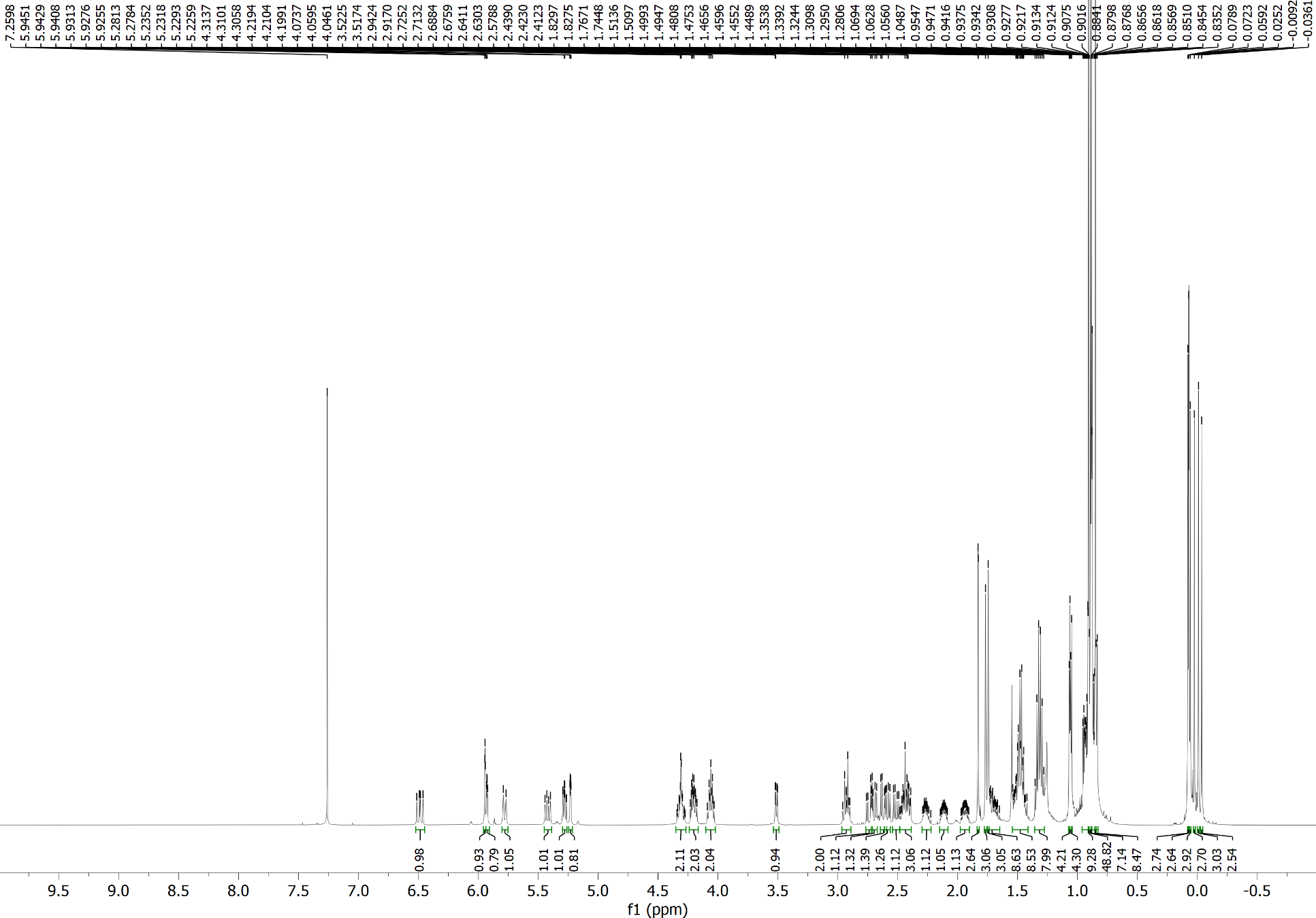


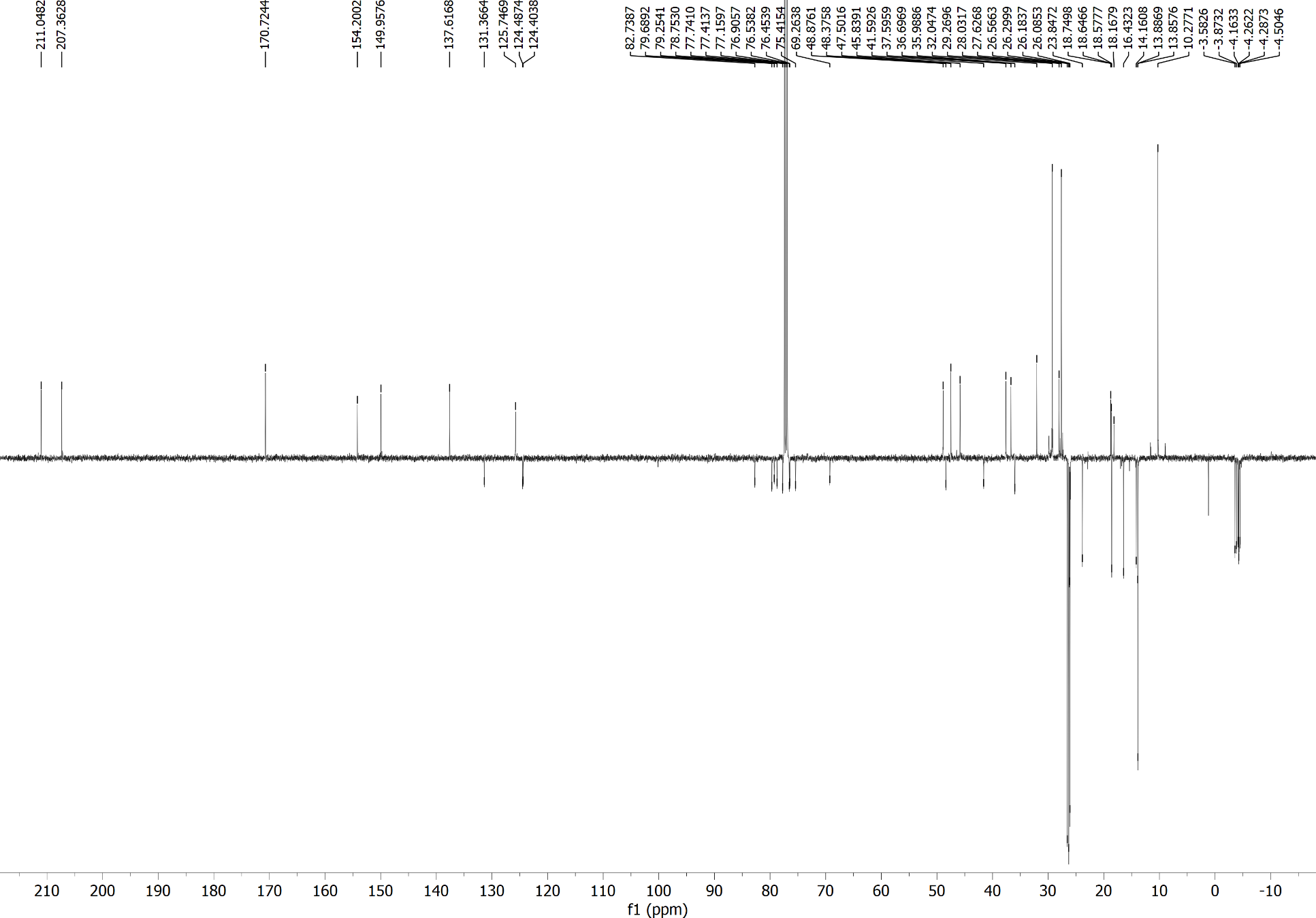

Supplement: Supplementary file 1 — ol2c03045_si_001.docx [file ol2c03045_si_001.docx]
